# Inference of lineage hierarchies, growth, and drug response mechanisms in cancer cell populations without tracking

**DOI:** 10.1016/j.crmeth.2026.101566

**Published:** 2026-08-17

**Authors:** Andrea Piras, Federica Galvagno, Letizia Pizzini, Matteo Nunziante, Sabrina J. Fletcher, Elena Grassi, Andrea Bertotti, Luca Primo, Antonio Celani, Alberto Puliafito

**Affiliations:** 1 INOC – Istituto Nazionale Oncologico Candiolo, IRCCS, Candiolo (TO), Italy; 2 Department of Oncology, University of Turin, Candiolo (TO), Italy; 3 The Abdus Salam International Centre for Theoretical Physics ICTP, Trieste, Italy

**Keywords:** cancer, phenotypic plasticity, inference, population dynamics, branching processes, lineage hierarchies, drug tolerance, Bayesian inference, Monte Carlo expectation maximization, patient-derived tumoroids

## Abstract

Lineage hierarchies and plasticity regulate development and tissue homeostasis, while diverted lineage dynamics and aberrant phenotypic plasticity are among the causes of incomplete drug response and resistance in cancer. Knowing the dynamics of phenotypically heterogeneous populations is therefore central to understanding growth regulation principles and to rationally design therapeutic approaches anticipating drug-tolerant states. While lineage inference can be addressed by barcoding technologies, these approaches often yield average clonal behaviors that neglect the underlying phenotypic plasticity of individual cells. Directly observing single-ancestor pedigrees in multi-type populations remains an experimental challenge. To address these difficulties, we developed a method to infer active phenotypic transitions in a multi-type tumor or clone and to quantify them, solely relying on counting cell-type abundances. We demonstrate the effectiveness of our approach to address cancer phenotypic heterogeneity and drug tolerance *in silico*. We then perform experiments on cancer cell populations and infer growth mechanisms and transition probabilities.

## Highlights

• A stochastic branching process infers cell-state transitions without lineage tracking
• Cell state switches are inferred from counts in multiple data types
• The method can resolve drug-induced transitions that sustain tolerant cancer states
• Inference reconstructs LGR5/KRT20 dynamics in patient-derived colorectal tumoroids

## Motivation

Understanding how phenotypic heterogeneity and lineage plasticity are dynamically regulated is critical for uncovering the mechanisms underlying incomplete drug responses and therapy resistance in cancer. Lineage-tracing approaches, such as cellular barcoding, effectively capture average clonal behaviors but often fail to resolve the stochastic single-cell state transitions that drive population-level adaptation. Conversely, directly tracking individual cell lineages across heterogeneous cellular environments remains experimentally challenging. Here, we present a model-based statistical inference framework grounded in multi-type branching processes that reconstructs lineage hierarchies and state-transition probabilities from static snapshots of clonal population composition, enabling the characterization of cellular state dynamics and rare subpopulations without continuous ancestry and cell-type tracking.

## Graphical abstract

**Figure:**
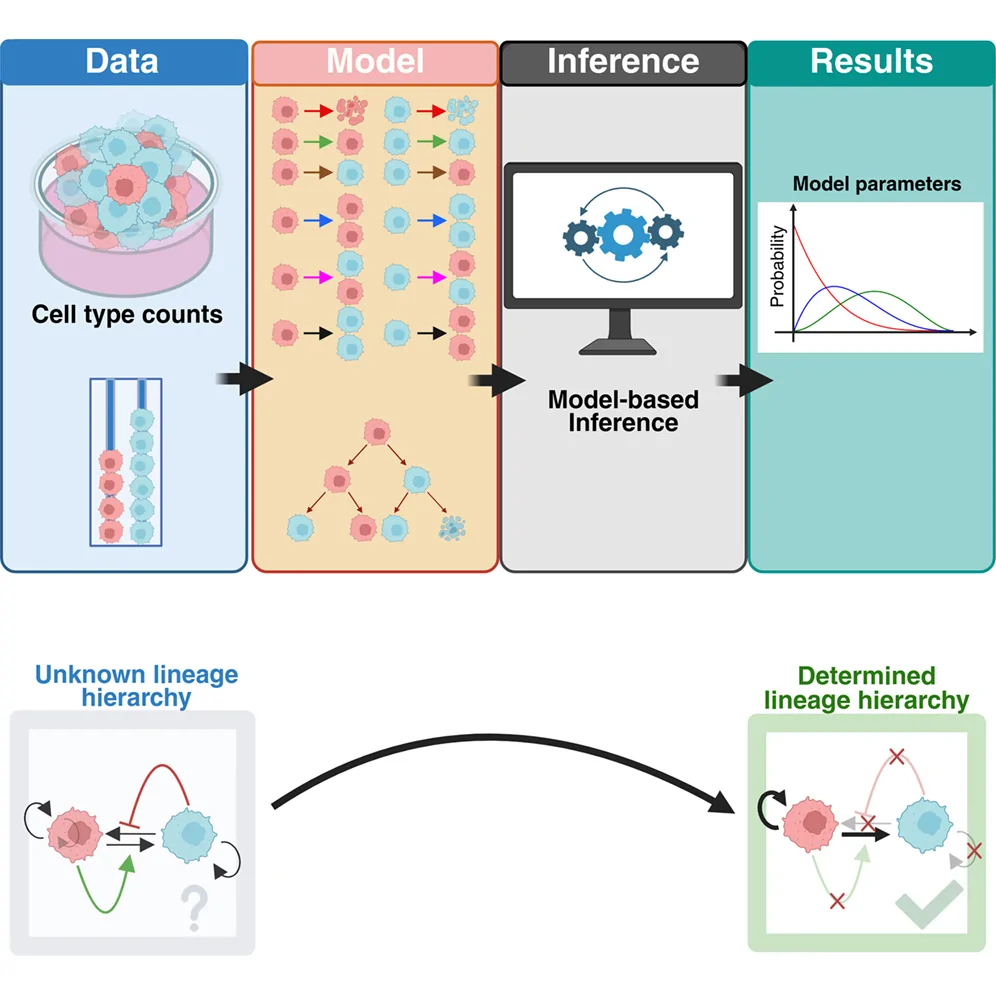

## Teaser

Piras et al. present a statistical framework to infer lineage hierarchies and phenotypic transition probabilities in heterogeneous cell populations without tracking individual cells. Using multi-type Bienaymé-Galton-Watson branching processes, the method reconstructs growth, plasticity, drug-response mechanisms, and LGR5/KRT20 dynamics in colorectal cancer tumoroids from cell-state abundance data.

## Introduction

Tissue development and homeostasis in metazoans rely on the stability of phenotypic identity. In embryonic and tissue development, tightly regulated phenotypic switches are at the basis of the establishment of lineages, and hence cellular functions, while plasticity is commonly associated with tissue repair upon insult.

In cancer cells, acquired cell plasticity can result in a rewiring of normal lineage hierarchies.^1^^,^^2^^,^^3^^,^^4^^,^^5^^,^^6^^,^^7^^,^^8^^,^^9^^,^^10^ Importantly, cancer cell plasticity has also been shown to contribute to therapeutic tolerance and to the emergence of secondary resistance.^11^^,^^12^^,^^13^ Therefore, it is crucial to relate phenotypic transitions in untreated vs. treated tumors in order to reconstruct the effect of the therapy on lineage hierarchies and its impact on drug treatment.

In practice, characterizing phenotypic switches for a set of cell types within a lineage involves determining all possible transitions, i.e., cell differentiation, self-renewal capabilities, controlled cell death, and asymmetric or symmetric cell division. Characterizing the behavior of a particular cell type *in vivo* requires a number of complex experimental approaches involving clonal analysis and lineage tracing, i.e., observing transitions in real time and/or experimentally tracking the progeny of one (or a few) cells in order to determine the phenotypic traits and phenotypic state or type of the descendants.^14^^,^^15^^,^^16^^,^^17^^,^^18^^,^^19^^,^^20^^,^^21^^,^^22^^,^^23^ These approaches however are experimentally challenging even when applied to developmental processes which are biologically well characterized and become often prohibitive when applied to cancer.

Observing phenotypic transitions in complex biological models is indeed difficult, especially when single-cell level observations are required, due to the necessity of genetically engineering multiple reporters and to follow the dynamics over time. Dynamical measurements are possible by combining the use of fluorescent reporters and that of live microscopy, albeit with a relatively limited throughput.^24^^,^^25^^,^^26^ Dependence on the progression stage of the tumor, patient variability and unknown and potentially changing genetic or transcriptional markers make these experimental approaches particularly complex. On the molecular side, the advent of single-cell technologies has significantly increased the ability to investigate phenotypic heterogeneity in tissues and several techniques allow us to infer phenotypic trajectories in single-cell datasets (see for example Deconinck et al.^27^) as well as to spatially resolve phenotypic heterogeneity within a tumor. While these techniques can reconstruct a pseudo-temporal order or even estimate probabilities of phenotypic transitions,^28^ they do not have the capability to dissect differentiation mechanisms. For all these reasons, effective quantitative techniques to quantify phenotypic transitional probabilities and that can bypass experimental difficulties are needed.

Classical control theory and continuous model approaches have been applied to infer growth mechanisms in the developing crypt and intestinal tissues.^29^^,^^30^ Theoretical approaches to decipher the design principles of biological tissue growth have also been followed, combining growth regulation models and lineage dynamics control,^31^^,^^32^^,^^33^ and suggesting therapeutic strategies to counteract cell plasticity under drug treatment.^34^ Specifically, several approaches have been dedicated to lineage reconstruction starting from the observed statistics or dynamics.^35^^,^^36^^,^^37^^,^^38^^,^^39^^,^^40^^,^^41^^,^^42^^,^^43^^,^^44^^,^^45^^,^^46^^,^^47^ Inference of progenitor types and dynamics can, for example, be performed without witnessing single-cell-level transitions,^48^ just by inferring rates from the asymptotic size distribution of clones derived from single cells *in vivo*. Many of these approaches, however, rely on knowing cell histories by tracking, i.e., knowing the complete lineage tree—or pedigree—at all times, or the direct observation of transitions, or alternatively, require to know in advance what transitions are taking place in the experimental model or tissue. Furthermore, additional difficulties are represented by the fact that inferring from small clones or tissue is potentially sensitive to stochasticity and noise, and that transitional probabilities could potentially be changing over time, calling for new approaches based on different theoretical frameworks.

Here, we propose a suite of inference methods to obtain single-cell transition probabilities from experimental data of multi-type tumors by assuming only knowledge of the number of cells in each cell type, without tracking of sort or the need of knowing the pedigree. Our method bypasses the need to directly witness the transitions experimentally and relies solely on the ability of identifying and counting how many cells are found in a particular transcriptional state or type. The proposed approach consists of defining all possible transitions with no prior knowledge and quantitatively inferring the transition probabilities compatible with the experimental observations. This allows one to exclude absent transitions when inferred probability is zero.

We present both Bayesian and non-Bayesian, analytical and numerical approaches based on the well-known Bienaymé-Galton-Watson (BGW) stochastic branching processes.^49^^,^^50^^,^^51^ Such a choice naturally allows one to take into account phenotypic switches as well as cell deaths, self-renewal, symmetric/asymmetric divisions and extinction for each clone or cell population. We show that we can infer lineage hierarchies in growing multi-type tumors (such as patient-derived tumoroids) with no prior information. We demonstrate that our approach can unveil the phenotypic mechanism of action of a drug in a multi-type tumor with previously unknown plastic transitions fueling drug-tolerant states. Finally, we apply our inference methods on experimental data of cancer cell populations, hematopoietic stem and progenitor compartment, and patient-derived tumoroids demonstrating the efficacy of our approach.

## Results

### Inference of phenotypic transitions in a BGW branching process

We sought to infer population dynamics in a multi-type tumor where in principle many different phenotypic transitions are possible. For each cell, these are cell death, inactivity, and self-renewal and then all possible phenotypic switches from one type to another, symmetric and asymmetric cell division into any other type or types (Figure 1A). We here neglect genetic heterogeneity and focus on phenotypic heterogeneity only. We also refer the reader to the glossary of terms used throughout the paper (available in Table S1) in order to clarify definitions and assumptions. Details on the mathematical model allowing to infer transitions are given in the STAR Methods section. Given a set of observations W representing the experimental data, we consider several scenarios that our approaches can handle (Figure 1B). The case of cell tracking is represented by observations where cell identity and transitions are known at all times, typical of live microscopy (Figure 1D). This datasets therefore consists in several repeated series where single cells give rise to different trees that are completely known. This is equivalent to say that the full pedigree is available. A second type of observations is represented by consecutive measurements without cell tracking (Figure 1E). Here, we suppose we can count cells of any type at all times without however knowing relations and transitions between them. A third data type is represented by connected endpoint measurements, where only the initial and final states are known, as in a fixed endpoint snapshot (Figure 1F). Last, we consider the case where we have snapshots obtained at different times, which however started from different unrelated ancestors (Figure 1G). For a more complete set of examples of what kind of biological data our approach might cover, we refer the reader to the STAR Methods.

**Figure 1:**
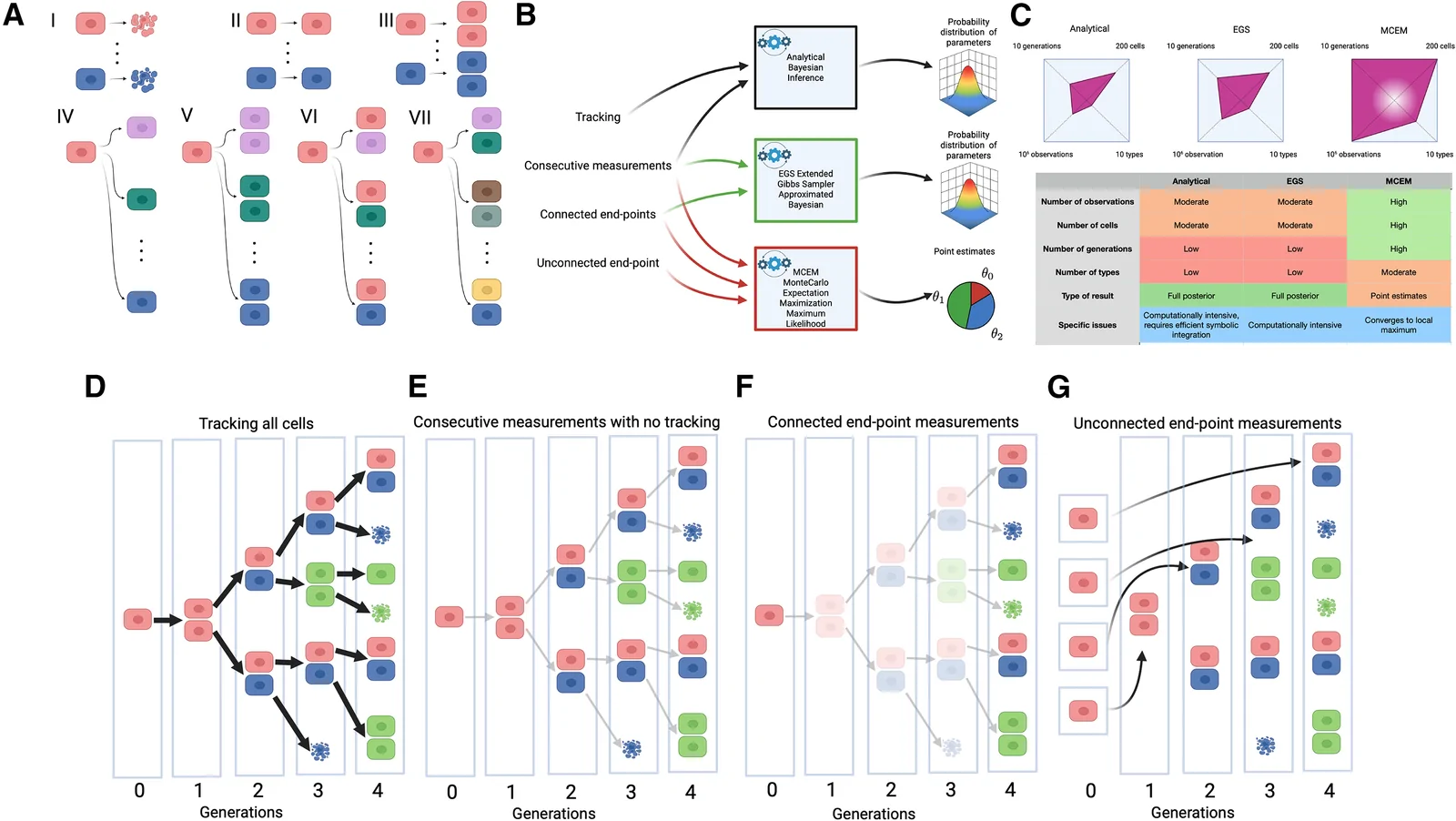
Bienaymé-Galton-Watson branching processes and inference for cell populations (A) Illustration of possible transitions in BGW cell population model. (I–III) Transitions corresponding to cell death, inactivity, and self-renewal respectively for all types. (IV) Differentiation into another phenotype. (V) Symmetric cell division where the cell type of the offspring is distinct from that of the ancestor. (VI andVII) Asymmetric cell division in the two different cases where one of the daughter cells is homotypic with the mother cell (VI) and where both daughters are different from the mother (VII). (B) Types of data input W considered throughout the paper and corresponding output for each distinct method developed here. (C) Phase portrait for the different methods. Top: qualitative phase portrait of each of the methods presented in the manuscript, to highlight the different regions of applicability of those methods. The shaded region at the center of the MCEM portrait indicates the fact that MCEM typically requires a moderate amount of data in order to work properly. Bottom: qualitative comparison between the different methods and specificity of each. (D–G) Types of data W we consider throughout the paper. Schematic created with BioRender.com.

We aim at estimating the probability of all transitions in the cases presented previously, particularly when tracking is not available. To this end, we developed a number of different approaches based on the known BGW stochastic branching process. In order to handle experimentally relevant situations, we followed three different strategies (Figure 1B). In the first (the analytical method) and second (the extended Gibbs sampler [EGS]) methods, we employed Bayesian inference, in order to estimate the posterior probability of the phenotypic transitions by knowing the probability of observing a particular observation as a function of the parameters. Details are given in the STAR Methods section. For datasets with a large number of cells, observations, or cell types, Bayesian inference becomes computationally challenging and we resorted to a third computational approach called Monte Carlo expectation maximization (MCEM). While computationally more efficient, this is a maximum likelihood approach and can only output point estimates for the transition probabilities, rather than entire distributions. A description and comparison of the features of each of these approaches is given in Figures 1B and 1C, while the type of data we can handle is described in detail in Figures 1D–1G and further explained in the STAR Methods.

### Inferring transition probabilities in cancer populations *in silico*

In order to show the effectiveness of our approaches we generated numerically simulated data as in Figures 1D–1G, representing several biologically relevant examples. We then employed our inference method to identify which transitions generated the data and their probability and compared the ground-truth parameters with the results of the inference. Detailed examples with 1 cell type and 3 cell types are presented in the STAR Methods.

#### The growth of a two-type cell population

We begin with the simplest case of unconstrained growth of two distinct non-interacting cell types. A sketch of the simulated model is shown in Figure 2A. This population structure applies to several different biological contexts and corresponds to the case where stem cells or progenitors self-renew and give rise to differentiated cells which can only be shed^14^^,^^16^^,^^29^^,^^52^ or die off. We assume that the initial population consists of a single cell of type 1 (progenitor) in order to mimic the higher clonogenic potential and the typical situation encountered in cell culture or in clonal development. Note that inference is performed with no prior knowledge, meaning that, for example, differentiated cells are allowed in principle to become stem cells. A set of typical realizations of the numerically simulated model can be seen in Figure 2B.

**Figure 2:**
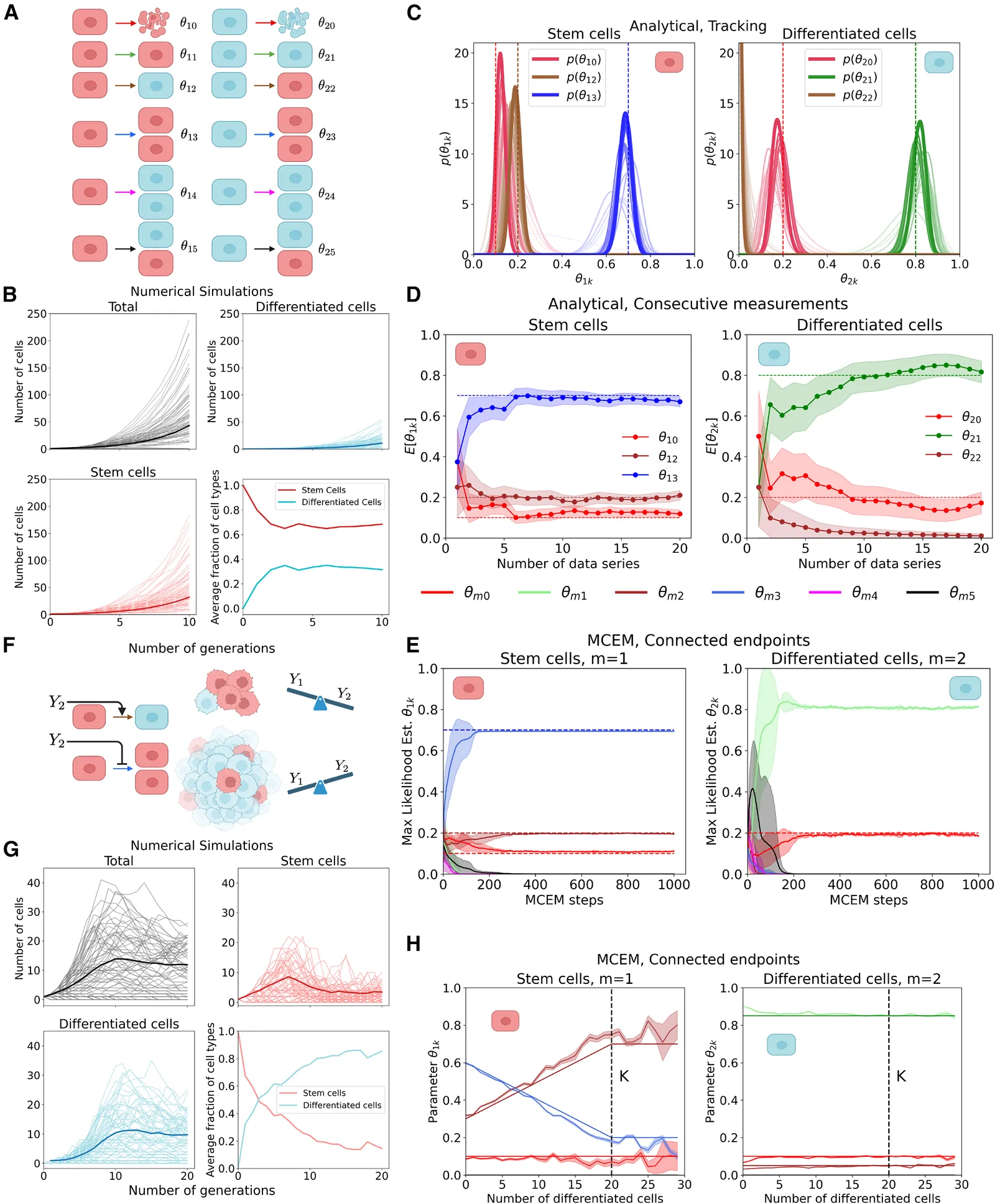
Growth of two-type populations (A) Sketch of the full model and parameters used for inference. (B) Results of numerical simulations of a two-type BGW branching process with the number of stem cells/progenitors and differentiated cells over the course of generations. The thin light hues refer to single realizations, while thick darker line is the average. The 120 sequences shown were generated starting from 1 cell and lasting 10 generations. (C and D) Inference with analytical method using a reduced model with true parameter values ${\vec{\theta }}_{1}^{\text{true}}=\left(\theta_{10}=0.1,\theta_{12}=0.2,\theta_{13}=0.7\right)$ and ${\vec{\theta }}_{2}^{\text{true}}=\left(\theta_{20}=0.2,\theta_{21}=0.8,\theta_{22}=0\right)$. Note that parameter *θ*_22_ is included in inference but set to zero in data. Bayesian inference of parameters with cell tracking is shown in (C). In this dataset the likelihoods were $L_{1}=\theta_{10}^{32}\theta_{12}^{50}\theta_{13}^{182}$ and $L_{2}=\theta_{20}^{28}\theta_{21}^{132}$. Inference with analytical method when tracking information is not available is shown in (D). Shades represent standard deviation over multiple restarts. (E) MCEM estimates with the full model as in (A). End-point measurements after 6 generations were used as data. Dashed lines represent the true value for each parameter, indicated by a different color, as detailed in the color legend. Shades represent standard deviation over multiple restarts. (F) Extension of the model as in (A) with illustration of the role of feedback on the different transitions. Model parameters used to generate data were ${\vec{\theta }}_{1}^{\text{true}}\left(0\right)=\left(\theta_{10}=0.1,\theta_{12}=0.3,\theta_{13}=0.6\right)$, *K* = 20, *θ*_13_(*K*) = 0.2 and ${\vec{\theta }}_{2}^{\text{true}}=\left(\theta_{20}=0.1,\theta_{21}=0.85,\theta_{22}=0.05\right)$. True values for all values of *Y*_2_ are shown in (H). Parameters not appearing on the plots are set to 0 in the simulations but left free in inference. (G) Numerical simulation of the process in (F): (black) total population size; (red) stem cell/progenitor compartment; (cyan) differentiated compartment. Lower right plot shows the average fraction of stem cells/progenitor (red) to differentiated cells (cyan) over the generations, showing the two distinct regimes of growth. (H) Results of inference from data shown in (F) and (G) by using MCEM on stem cell/progenitor parameter sets (left) and differentiated parameter sets (right). Parameters not appearing on the plot are 0 in the simulations but left free in inference. Shades represent standard deviation over multiple restarts. Schematic created with BioRender.com.

#### Results with cell tracking

As shown in the single-cell-type case (see STAR Methods), tracking reduces to keeping track of the cumulative counts of all observed transitions, which allows the analytical posterior distribution to be obtained directly. Figure 2C shows the results obtained inferring data obtained with numerical simulations of 1 single progenitor growing for 4 generations and giving rise to structured aggregates. Note that due to computational limitations, we were only able to use a reduced model from Figure 2A, including only a subset of parameters (*θ*_10_,*θ*_12_,*θ*_13_,*θ*_20_,*θ*_21_,*θ*_22_). As evident from the plot, the marginal distributions of the inferred values are consistent with the parameter values used to simulated data and converge to the ground truth values when increasing the number of processed data series.

#### Consecutive measurements with no tracking

We then inferred parameters by employing both the analytical method and the EGS assuming no tracking information is available. The feasibility of this approach is demonstrated in Figure 2D, where Bayesian inference is applied recursively. Indeed, our results show that, while data accumulates, the estimates of the parameters converge to the true values. Our analysis also reveals that the level of uncertainty is different in the two parameter sets, due to the relative proportions of the two populations (fewer differentiated cells than progenitors) in each clone and hence in the dataset. It is also worth noting that when prior biological information is unavailable, the full model, which accounts for all possible transitions, must be used. This results in the inference of a relatively large number of parameters, mapping into pronounced variability unless large amount of data is used, which can be a computationally intense task.

#### Endpoint measurements

Provided that enough data are available, this issue can be addressed using the MCEM algorithm. Notably, this approach remains applicable even when the only available information is the count of the number of cells in each sub-population at the end of the experiment, as typically happens when fixing samples. We demonstrate the suitability of this approach by using 10,000 observations representing the size of the two sub-populations after six generations, generated using the previously specified ground-truth parameter values. This time however, thanks to the better computational performances of the MCEM method, we were able to use the complete model as shown in Figure 2A. The results presented in Figure 2E show the convergence of the point estimates obtained with the MCEM method to the true parameter values. We note that our approach can sort out the specific transitions occurring in the dataset by inferring the parameters: death, self-renewal and differentiation of stem cells or progenitors, and death and quiescence of differentiated cells. Therefore such an approach can guide the discovery of lineage hierarchies in a structured population which can then be verified experimentally. It is worth noting that while MCEM is proven to converge, it may only reach a local maximum of the likelihood and not the global maximum. This happens routinely especially in high-dimensional parameter spaces. To effectively reach a global maximum, the algorithm can be restarted multiple times with different initial parameter values. In the absence of the knowledge of the true parameter values, the final likelihood values obtained from different initial parameter configurations can be used to select the parameter set corresponding to the highest likelihood value (see STAR Methods).

### The case of chalones: Two types with feedback

A natural extension of the two-phenotype free growth scenario is the case where two interacting phenotypes coexist. Here, transition probabilities fuel population dynamics and, in addition, one sub-population can also modulate the growth of the other by feeding back into transition probabilities. Biologically this can occur through paracrine or autocrine signals, such as growth factors or chalones. Similar examples are discussed in Itzkovitz et al.^29^ or in Lander et al.^33^, where the balance between asymmetric and symmetric stem cell division is modulated in order to improve developmental times. We note here that cell proliferation can occur through different transitions depending on the value of the corresponding parameters and these events are associated to a different average duration, therefore allowing not only different types to divide with heterogeneous doubling times but also in principle to associate different division events within the same cell type to different average doubling times. To evaluate our ability to infer the feedback mechanisms regulating sub-population dynamics, we selected a specific set of feedback interactions consistent with a scenario where a steady state can be achieved, starting from a single initial stem cell or progenitor. Specifically, we employed the following model for the feedback (Figures 2A–2F):

(Equation 1) $$\left\{\begin{array}{l} \theta_{10}\left(Y_{2}\right)=\theta_{10}\\ \theta_{12}\left(Y_{2}\right)=1-\theta_{10}-\theta_{13}\left(Y_{2}\right)\\ \theta_{13}\left(Y_{2}\right)=(\theta_{13}-\theta_{13}\left(K\right))\left(1-\frac{Y_{2}}{K}\right)(1-H\left(Y_{2}-K\right))\\ +\theta_{13}\left(K\right) \end{array}\right)\;\left\{\begin{array}{l} \theta_{20}\left(Y_{2}\right)=\theta_{20}\\ \theta_{21}\left(Y_{2}\right)=\theta_{21}\\ \theta_{22}\left(Y_{2}\right)=\theta_{22} \end{array}\right)\text{,}$$

where parameters that are not specified are assumed to be zero, *H*(*Y*_2_-*K*) is the Heaviside function (*H*(*x*) = 1 when *x* ≥ 0 and *H*(*x*) = 0 elsewhere), and *θ*_13_≥*θ*_13_(*K*).

In this model, progenitors (*m* = 1, Figure 2H continuous lines) can proliferate, differentiate, or die. While the death probability remains constant, both proliferation and differentiation probabilities depend on the population size of differentiated cells. As the latter population grows in each tissue unit, or tumoroid, progenitors increasingly favor differentiation over self-renewal up to a certain stable situation reached around a critical differentiated cell population *K*. Note that differentiated cells can (albeit with a small probability) de-differentiate. A notable consequence is that differentiated cells can replenish the progenitor population in cases where the progenitor compartment has been depleted or eliminated, such as after injury, treatment, or targeted ablation.^8^^,^^53^^,^^54^^,^^55^^,^^56^ Data generated with this model exhibit bounded overall growth in both populations but show two different regimes: one where stem cells prevalently self-renew and therefore prevail over differentiated cells, and one later regime where a constant ratio between the two types is maintained, in favor of differentiated cells. Results of the simulation are shown in Figure 2G. This situation can be tackled with the MCEM approach. The data containing the number of cells for each phenotype at each generation are used as input for the MCEM algorithm with the aim of inferring the population structure and the relations among the different compartments, i.e., the feedback mechanisms. We note that without any prior knowledge, one should perform inference for each (*Y*_1_,*Y*_2_) pair individually and verify *a posteriori* whether parameters are constant along one direction (in this case *Y*_1_). This approach can be followed using MCEM but requires a large amount of data for each pair in order to obtain reliable estimates. Alternatively, one can hypothesize a dependence only on *Y*_2_ or introduce a prior knowledge suggesting such a dependency. In this case, one can directly group the data of the type (*Y*_1_,*Y*_2_ = *y*_2_) and infer the parameters as a function of *Y*_2_. The comparison between the true and inferred parameter values in Figure 2H illustrates this latter approach and demonstrates the capability of our method to uncover the underlying feedback mechanisms. It is also worth noting that inference quality degrades over the number of differentiated cells. Analogously to the previous case, this is due to the number of observations being more and more rare for large *Y*_2_, determining a consequently larger standard deviations of the inferred parameters.

### Inferring transitions under drug treatments

Another biologically relevant context for our approach is that of inferring lineage hierarchies in a population that is under drug treatment. In the context of cancer therapy (and in other pathological contexts as well), it is indeed relevant to know how effective is a given drug treatment on cell populations and on lineage dynamics. Within multi-type populations, beyond standard cell-level effects, this can be impacting on differentiation probabilities or self-renewal probabilities or yet target populations to different extents. In particular, in the context of tumor phenotypic plasticity, it becomes relevant to monitor whether a drug treatment is inducing the emergence of new drug-tolerant phenotypes which might fuel relapse or resistance. Within the framework of our work, this problem can be reworded into altered transition probabilities, even in the absence of clear hypotheses or prior knowledge. We considered the case of a 3 cell-types tumor (Figures 3A–3C), where a population of cancer stem cells can self-renew, differentiate or reversibly transition into a slow cycling state.^7^^,^^9^^,^^10^^,^^57^ Here, differentiated cells are post-mitotic and are only progressively dying out. We imagine the effect of the drug to be impacting on the death of differentiated cells and cancer stem cells while having only minor effect on the plastic—drug-tolerant—slow cycling cells. Under treatment, the overall population shows a significant shrinking, but a small, drug-tolerant, population of cells is left behind and is reminiscent of minimal residual disease.^58^^,^^59^ This situation is clinically relevant, as emerging populations represent a promising target for tumors showing incomplete drug response.

**Figure 3:**
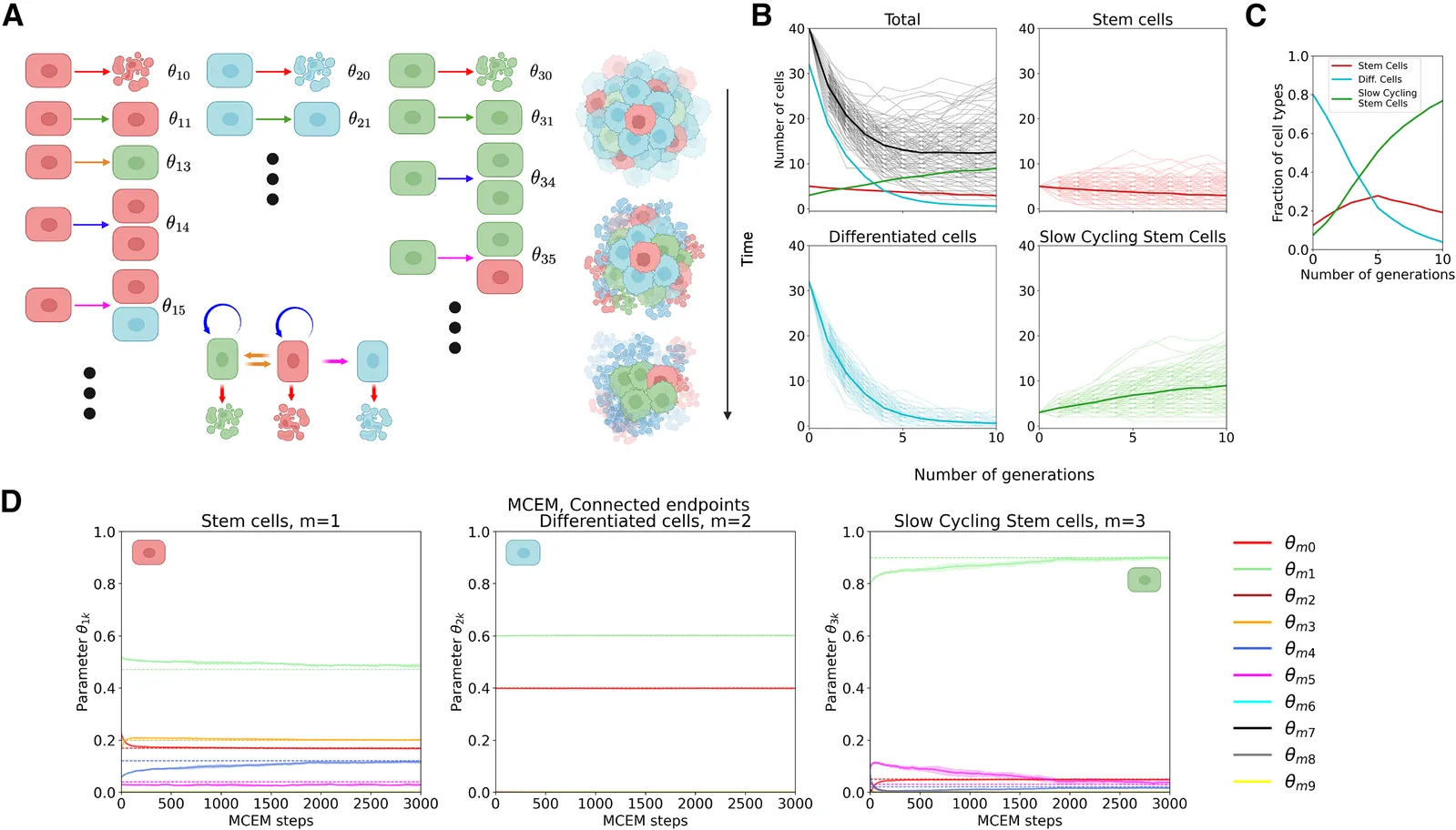
Inferring the effect of a drug on cell populations (A) Sketch of the model used to illustrate inference in a multi-type treated plastic tumor, made out of 3 phenotypes with stem (red, *m* = 1) and differentiated (cyan, *m* = 2) cells along with a minor population of cells (green, *m* = 3) constituting a population of reserve slow cycling cells that can repopulate the tumor. The parameters used are: ${\vec{\theta }}_{1}^{\text{true}}=\left(0.17,0.47,0,0.2,0.12,0.04,0,0,0,0\right)$; ${\vec{\theta }}_{2}^{\text{true}}=\left(0.4,0.6,0,0,0,0,0,0,0,0\right)$; ${\vec{\theta }}_{3}^{\text{true}}=\left(0.05,0.9,0,0,0.02,0.03,0,0,0,0\right)$. Schematic created with BioRender.com. (B) Numerical simulations of a treated multi-type tumor as in (A). In these numerical simulations, the tumor is initially composed of $\vec{Y}\left(0\right)=\left(5,32,3\right)$ cells. (C) Average fraction of cell types over the course of each simulation. (D) Maximum likelihood estimates obtained with inference performed with MCEM on data generated as in (B) for stem (left), differentiated (middle), and slow cycling stem (right) cells. Colors code for different parameters (transitions) as shown in the legend and match the color of the arrows in (A). Shades represent standard deviation over multiple restarts.

The effect of the therapy can be seen inferred with the MCEM method in Figure 3D. Here, we exploit data collected one generation after the beginning of treatment to infer the impact of the drug on phenotypic transitions. It is worth noting that, as one can expect, such an example is computationally quite demanding as the number of parameters to infer is large. In order to obtain starting conditions for MCEM and ensure convergence, we resorted to the method of moments (or moment matching, detailed in the STAR Methods). Our approach shows that one can determine what phenotypic transitions are involved by the drug treatment under consideration. For example, in the studied scenario, this would unveil a direct impact of the drug on the specific transition transforming slow cycling stem cells into stem cells, which can then repopulate the tumor (Figure 3D). Furthermore, once inferred, the parameters can be leveraged to calculate the extinction probability within the BGW formalism, and this can be used to evaluate the ability to eradicate the tumor in a particular therapeutic setting. All in all, the setting presented here shows the power of our approach in identifying potential drug-tolerance mechanisms based on therapy-induced populations and to rationally design population- or transition-targeted pharmacological approaches.

### Inference from experimental data

We then proceeded to apply our inference method to experimental data. In order to demonstrate the applicability of our approach, we took into consideration several representative experimental situations, with single or multiple types.

#### Unconstrained cancer cell growth

First, we seeded the cancer cell line MG-63 at low density in order to observe the offspring of single cells for 96 h and infer growth parameters. To this aim, cells were transfected with a lentivirus carrying H2B-iRFP670 transgene (Figure 4A). The data onto which we performed inference are therefore a collection of trajectories starting from 1 or 2 cells and followed over 96 h as shown in Figure 4B. The average doubling time was estimated as *τ*_2_=(23.1 ± 2.4) hours and clonal progenies were found to be substantially correlated for several generations, as shown by the distribution of doubling times within an offspring (Figure S4A). Indeed, the relative dispersion was around 10%, confirming that cells remain synchronous for several generations. This confirms the validity of BGW processes in describing trajectories. It is worth noting that this generally holds for mammalian cells, as it was shown that doubling time distributions follow approximately an Erlang distribution with a relatively large shape parameter^60^^,^^61^^,^^62^^,^^63^^,^^64^^,^^65^ (a more detailed discussion can be found in STAR Methods). We applied our inference method to the case when tracking is available, by using the model shown in Figure 4D. To this aim, we segmented and counted cells at all frames of our timelapse and detected cell death, identified readily by the absence of movement and by rapid decay of nuclear fluorescence and by evident cell fragmentation. Analogously we could detect cell divisions by looking at binary splitting of cells in the timelapse sequences. By doing so we were able to count the total number of divisions and deaths for each trajectory. Conveniently, when having a number of consecutive tracked observations until the generation *n*_*f*_, the total number of divisions and deaths are related by the relation

(Equation 2) $$\sum_{\text{obs}}Y\left(0\right)+N_{\mathrm{div}}-N_{\text{death}}=\sum_{\text{obs}}Y\left(n_{f}\right)\text{,}$$

where the sums are intended over all observations. This is because standbys do not change the final number of cells. Therefore, in practice, one can verify the correctness of the death events by directly counting cells and divisions, which with a nuclear marker such as that used in our timelapse is simple. Further, in order to determine all the transitions observed, one needs to count the standbys. We note that this is also straightforward: it is sufficient to determine the counts at all generations to write:

(Equation 3) $$N_{\text{inact}}=\sum_{\text{obs}}\sum_{k}^{n_{f}-1}Y\left(k\right)-N_{\mathrm{div}}-N_{\text{death}}\text{.}$$

**Figure 4:**
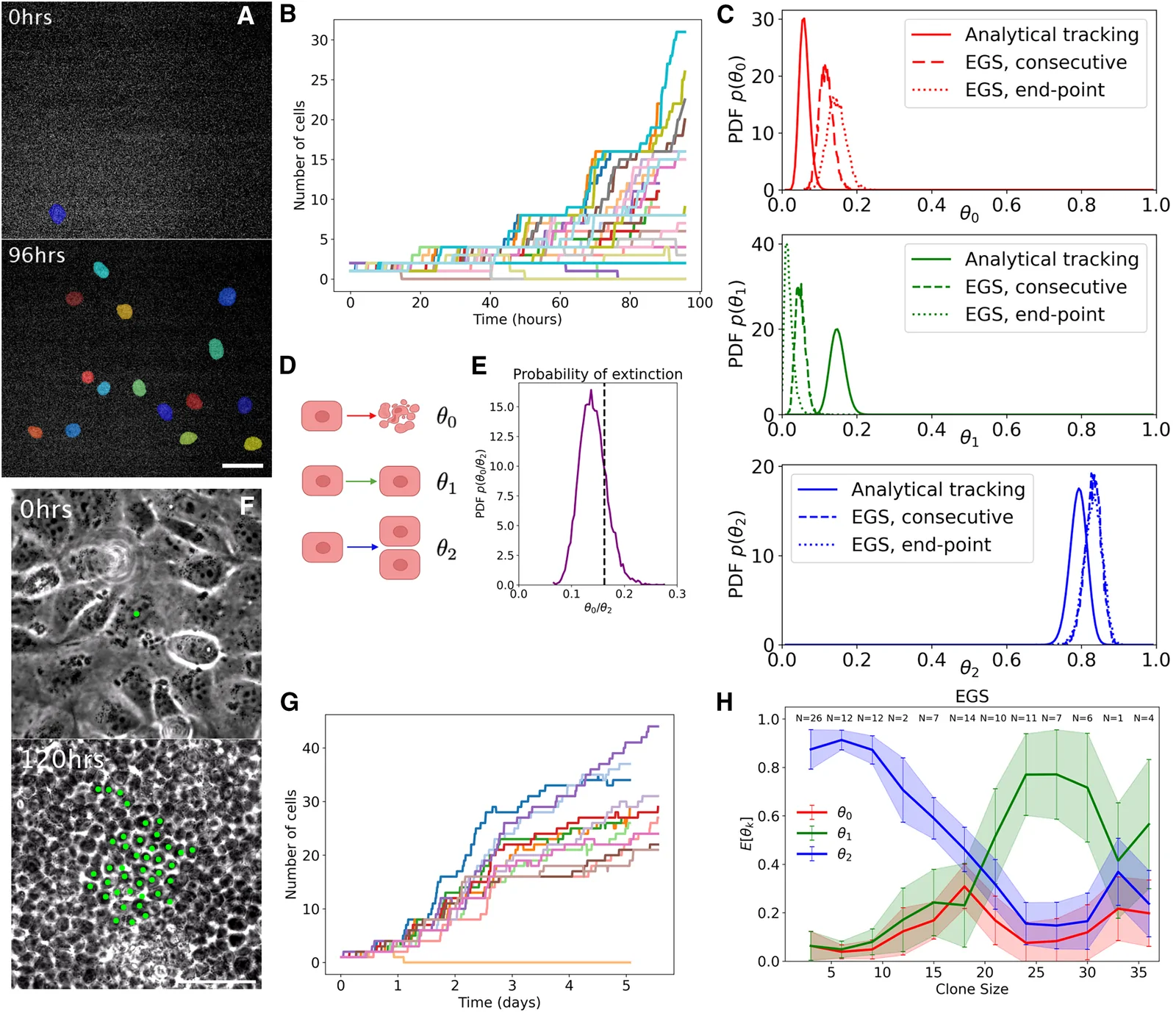
Inferring from experimental data: single type cellular systems (A) Representative snapshot of MG-63-H2BiRFP670 imaged and segmented at two different time-points cells. Scale bars: 50*μm*. (B) Single-cell trajectories monitored for 96 h. (C) Inference of data shown in (B) with analytical tracking and EGS for consecutive or endpoint measurements. (D) BGW model used for inference. Schematic created with BioRender.com. (E) Probability of extinction estimated through inference of parameters (violet distribution) compared with the experimental value (dashed line). (F) Representative snapshots of MDCK cells from Puliafito et al.^60^ tracked over more than 5 days. Scale bars: 50*μm*. (G) Single-cell trajectories monitored for more than 5 days. (H) EGS inference of data shown in (G). Shades and bars represent standard deviation of the posterior distribution.

Once the numbers of transitions have been determined one can directly write the posterior as previously: $p\left(\Theta |Y\right)∼\theta_{0}^{N_{\text{death}}}\theta_{1}^{N_{\text{inact}}}\theta_{2}^{N_{\mathrm{div}}}$. For a set of 37 trajectories lasting 4 generations the results are shown in Figure 4C. It is worth noting that while knowing all transitions for all cells (i.e., tracking) contains all the information to write down the posterior, it is indeed sufficient to count cells at all generations and count all cell division events regardless of the generation in order to write the posterior. When cell divisions are difficult to track and only counts are available, we can resort to the other methods developed here. We therefore used the EGS to infer parameters without tracking but resorting to consecutive and endpoint measurements. Results are shown in Figure 4C. It is worth noting that while being from the same experimental dataset, data are not at all equivalent and, correspondingly, inference gives different results. Indeed, since we have no tracking information, there are many more compatible trees to go from one point to another, resulting in slightly different estimation of the parameters. A more detailed discussion of this aspect can be found in the STAR Methods. We note that the inferred parameters can help predicting population properties such as the extinction probability, which can be readily calculated as *p*_*e*_ = *θ*_0_/*θ*_2_ when *θ*_2_>*θ*_0_ and *p*_*e*_ = 1 otherwise. The probability of extinction at every generation can also be calculated simply by collecting terms in zero-th order in the composite probability generating function (PGF) of the BGW process (see STAR Methods and Haccou et al.^49^). Notice that in the entire database considered, 6 out of 37 clones went extinct, consistent with the extinction probability calculated theoretically and shown in the inset of Figure 4E.

#### Epithelial contact inhibition

We then considered the case of interacting cells and employed data collected in ref.^60^ of MDCK (Madin-Darby Canine Kidney) cells growing onto a 2D substrate (Figure 4F). We tracked the progeny of single cells starting from sub-confluent regimes and followed the offspring for more than 5 consecutive days in order to see the slow-down of cell proliferation (Figure 4G). As before, we estimated the relative dispersion of doubling times within the initial free growth regime and found it to be of the order of 10%, consistently with the previous case of unconstrained growth (Figure S4B). We used these data to infer population parameters. Results obtained with the EGS are shown in Figure 4H. Inferred parameters clearly show that the probability of cell division per generation decreases over time in favor of an increased probability of being inactive, alongside a rather constant death probability that balances out rare cell division asymptotically. As specified before, no functional dependence of the transition probabilities on density has been assumed here. Results are consistent with what was found in ref.^66^, where cell division is found to be decaying with a power law.

#### Inferring transitions in a multi-state cell population

We then verified the ability of our approach to handle multiple states in a cell population, by selecting a two-phenotype system in which, among all theoretically possible transitions, only few are known to be effectively taking place. To this aim, we leveraged the FastFUCCI^67^ construct to monitor the cell-cycle phase in individual cells (as shown in Figure 5A). We reasoned that by assuming no prior on the possible transitions as usual, we would recover the only possible transitions observed in the cell cycle, i.e., one S/G _2_/M cell to two G _1_ cells (green to two red), and one G _1_ cell to one S/G _2_/M cell (red to green), all the other transition probabilities involving type change being zero (Figure 5B). These have a similar phenomenology to a system in which some kind of progenitor can divide by giving rise to two differentiated cells which can revert to the progenitor state. First we verified that even in this case, data could be modeled with a BGW branching process (Figure S4D). In order to apply our method, we built a dataset by segmenting cells and assigning a class (either green or red) by calculating the respective fluorescence intensities (details are given in the STAR Methods section). A sample of analyzed trajectories is shown in Figures 5C and 5D. By performing tracking on 20 trajectories, we were able to infer parameters, as shown in Figure 5E. Our results indicate that inference performed with all transitions (Figure 5B) identifies only the expected transitions, i.e., heterotypic cell division where a green cell divides into two red cells and type switch from red to green. In order to illustrate the power of our approach, we then performed inference assuming only consecutive measurements, i.e., observing only the counts of each type at each generation without knowledge of the pedigree. Results are shown in Figure 5F and are compatible with the data from tracking. Note that, as previously noted, data from consecutive measurements are less informative than data from tracking and yield different inference results (see STAR Methods).

**Figure 5:**
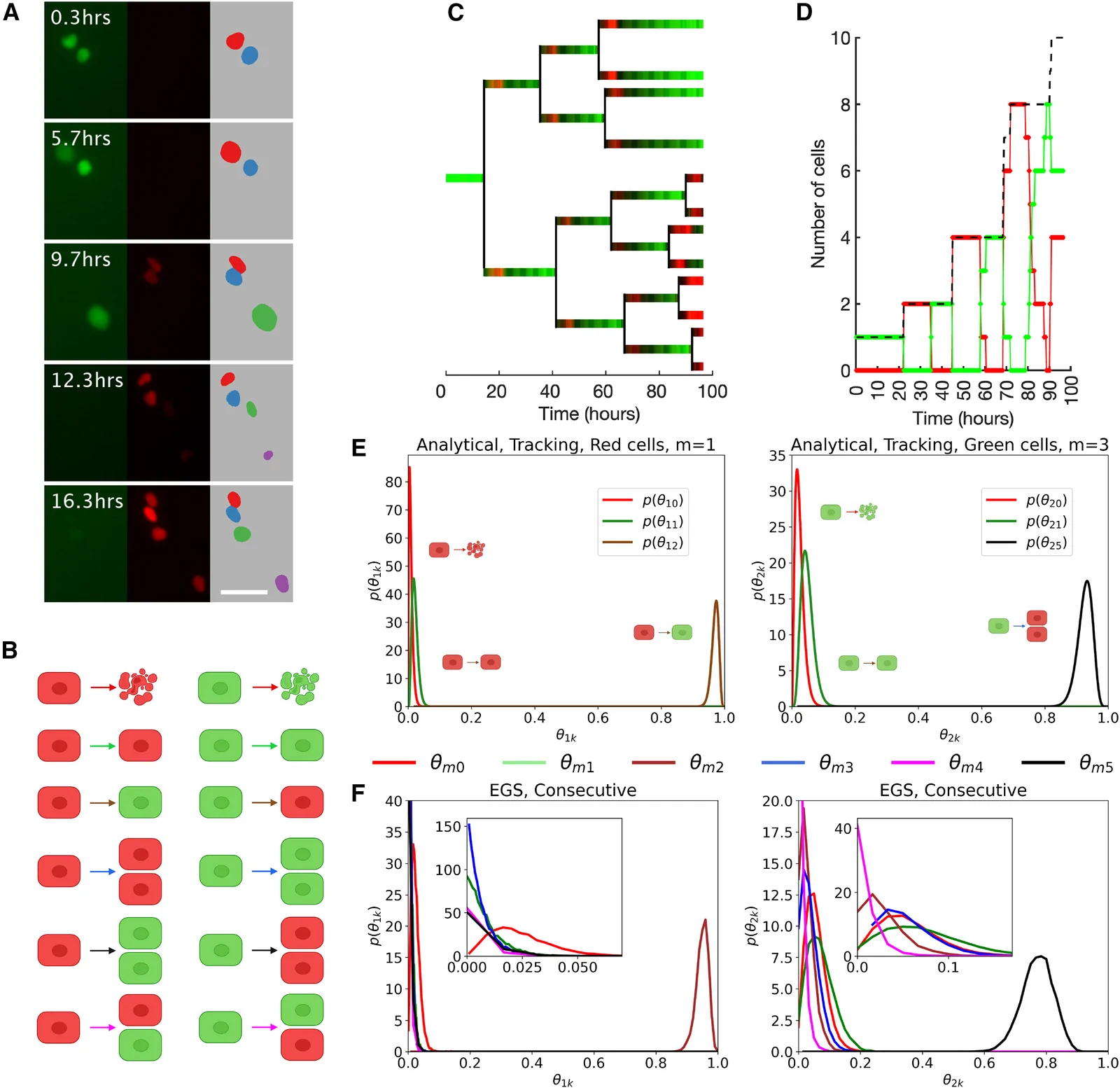
Inferring from experimental data: multi-state cellular populations (A) Representative snapshots of MG-63-FastFUCCI along with cell segmentation. (B) Sketch of the BGW model used for inference. Schematic created with BioRender.com. (C) Single-cell trajectory for one cell reconstructed and plotted over the course of the timelapse. Color shades are real fluorescent data for each cell. (D) Data series for a single green (S/G _2_/M) cell evolving into a small clone. Dashed line corresponds to the total number of cells, while red and green count the number of G1 and S/G _2_/M cells respectively. (E) Analytical inference of 16 trajectories with tracking. Colors match those of arrows in (B). (F) EGS inference on the same trajectories as in (E). Colors match those of arrows in (B).

#### Inferring transitions in the hematopoietic stem and progenitor compartment

As an example of multi-type cell population, we exploited the data presented by Wheat et al.^68^, which dissects the dynamics of hematopoietic stem and progenitor cells. Data (Figure 6A) consist in several measured trees of mouse hematopoietic cells tracked for 96 h with no access to cell type, except at the end, where cells are phenotypically characterized with single-molecule fluorescence *in situ* hybridization (smFISH). The authors use the expression of the master transcription factors *PU.1*, *Gata1*, and *Gata2*^68^ in order to classify cells into 4 different types named P1H (*PU.1*^high^*/Gata1/2*^low^); G1/2H (*Gata1/2*^high^*/PU.1*^low^); G2H (*Gata2*^high^); and LES (with low expression of all three genes), and perform inference on these 4 states by using kin correlation analysis.^35^ We retrieved raw data from Wheat et al.^68^, and performed endpoint inference with MCEM in order to extract transition probabilities by using only the endpoint states. To this aim, following the approach of Wheat et al.,^68^ we defined a minimal model (shown in Figure 6B). The minimal model is designed to be consistent with biological observations and to further dissect which transitions are most likely taking place given the data. Note that, compared to the original model proposed by Wheat et al.^68^, our model makes it possible to further unpack specific types of phenotypic switches and therefore includes a slightly larger number of parameters. Results are presented in Figure 6C and show that LES cells are inferred to be mostly self-renewing (with probability *θ*_12_ = 0.76) and perform symmetric differentiation giving rise to one P1H cell and one G2H cell. Conversely G2H cells mostly perform asymmetric division (i.e., divide into one G2H cell and one LES cell) with probability *θ*_23_ = 0.54 and self-renew (*θ*_22_ = 0.27). The probability of performing symmetric differentiation (i.e., dividing into a G1/2H cell and a LES cell) is very rare (*θ*_24_ = 0.01). G1/2H cells are found to be dividing with smaller probability than other cell types, coherently with their position within the differentiation hierarchy. Since the ancestor of each data tree collected is unknown, we also considered inference starting with different ancestor distribution (Figure S5A). Our data indicate the strong predominance of one type for all ancestors as highly unlikely, while more probable configurations include all four states as possible as ancestors. In this regime, parameters inferred do not appreciably depend on the initial distribution of ancestors, as shown in Figures S5B–S5E. It is worth noting that, here, we have access to separate phenotypic transitions and not just cumulative rates describing transitions. It is however possible to re-frame our results into aggregated probabilities, similar to the model presented by Wheat et al.^68^, (STAR Methods, Figure 6D) and to obtain comparable results.

**Figure 6:**
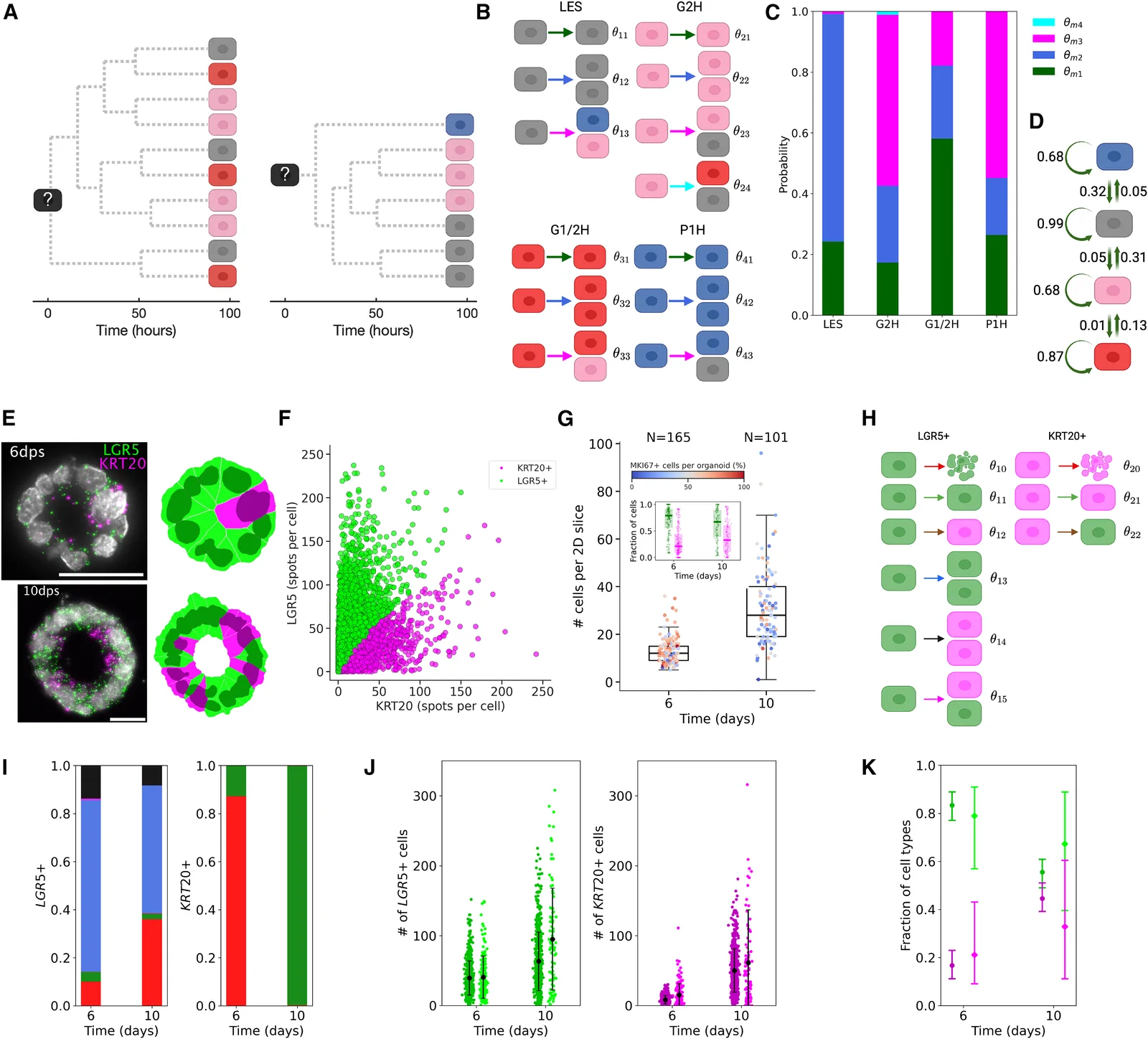
Inferring from experimental data: multi-type cell populations (A) Illustration of cell types and sample of trajectories from Wheat et al.^68^ The ancestor of each endpoint measurement is unknown. (B) Minimal model used to infer transitions from data, illustrating the different transitions considered. (C) Results of MCEM inference using endpoint only. Colors match those of arrows in (B). (D) Aggregated transition probabilities from (C). (E) Snapshots of a CRC organoid (6 days post-seeding [dps] on top and 10 dps on the bottom) stained with DAPI (gray) and with labeled *LGR5* and *KRT20* transcripts (green and magenta respectively). Scale bars: 30*μm*. The right side of each figure shows the digital segmentation, reconstruction and identified cell types (*LGR5*^+^ in green, and *KRT20*^+^ magenta). (F) Quantified number of spots per cell corresponding to each gene in all experimental conditions (6 dps and 10 dps), clustered depending on the level of expression of each transcript. Each dot in the plot represents a cell. (G) Distribution of cell sizes in 2D slices of organoids at each timepoint with color coding for the percentages of *MKI67*^+^ cells. The total number of analyzed organoids for each timepoint is specified on top of the plot. Inset: distribution of the fraction of *LGR5*^+^ and *KRT20*^+^ cells in 2D slices at the two time points. Boxplots indicate 25th, 50th, and 75th percentiles, while whiskers represent the most extreme data points extending beyond 25th and 75th for a maximum of the 25th–75th width. (H) Minimal model used to infer data from growing organoids illustrating all considered transitions. (I) Inference of parameters in samples collected at different time-points for *LGR5*^+^ and *KRT20*^+^ cells (left and right respectively). Colors match those of arrows in (H). (J) Data distribution obtained by simulated BGW (left, dark hues) compared to experimental data (right, light hues). Thick black lines represent mean and standard deviation. (K) Fraction of each cell type across the different time-points for simulated (left, dark hues) and experimental (right, light hues) data. Error bars are 25^th^ and 75^th^ percentile of the distributions. Schematic created with BioRender.com.

#### Inferring transitions in a multi-type tumor

As a final example, we considered a multi-type cancer cell population, by focusing on the LGR5/KRT20 hierarchy in colorectal cancer.^8^^,^^69^ According to published evidence, a subset of colorectal tumors, when untreated, present a defined lineage where *LGR5*^+^ cells possess stem-like properties, while *KRT20*^+^ cells are considered to be post-mitotic differentiated cells. By leveraging the biobank developed at our institute,^70^ we performed smFISH at different time points (6 and 10 days post-seeding [dps]) on a patient-derived tumoroid hybridizing for the transcripts of the genes *LGR5*, *KRT20*, and *MKI67* (Figure 6E; Figures S6A and S6B). Our data consist mostly of single cells or, more rarely, small multicellular aggregates developing into organoids with variable number of cells (Figure S6E). Cell states were derived by classifying each cell as *LGR5*^+^ or *KRT20*^+^ (Figure 6F; Figures S6C and S6D). By comparing the experimental data obtained at the two different time-points, we were able to show that while the total number of cells grows, the fraction of *MKI67*^+^ decreases from 6 to 10 dps (Figure 6G) and the fraction of *LGR5*^+^ cells decreases in favor of *KRT20*^+^ cells (Figure 6G inset). We then performed inference in order to dissect the different mechanisms by which *LGR5*^+^ cells give rise to *KRT20*^+^ cells. To this aim, we sampled the initial distribution (at zero-th generation) of cell states from classified data at 6 dps, as organoids are split around day 6. The number of elapsed generations was estimated by leveraging confocal timelapse performed with the same organoid transfected with a nuclear fluorescent marker (H2B-emiRFP670), as shown in Figure S6F. In order to perform inference, we opted for the model presented in Figure 6H. This model does not include the possibility of *KRT20*^+^ cells to proliferate in accordance with published data.^8^^,^^69^ This assumption is also consistent with our results showing decay of MKI67 levels with time (Figure 6G) and anti-correlation between *MKI67* expression and *KRT20* expression (Figure S6G). It is important to note that, by definition, endpoint datasets miss extinction events. In order to take this into consideration, we note that, on average, between 10% and 30% of seeded cells result in growing organoids. In order to verify the effect of extinction on our inference, we first considered organoids seeded as single cells and grown for 6 days, with variable number of extinct trajectories ranging from 0% to 80% (Figures S7A–S7C). Results show that inferred parameters do not vary appreciably when extinction probability falls within the experimental range. Furthermore, we note that extinction is quantitatively relevant at the first time point which is connected to seeding, as the probability of extinction rapidly converges at a constant level over generations. We then inferred parameters at 6 dps and used the inferred parameters to generate initial conditions to infer parameters at 10 dps. Our results indicate that two different solutions exist, with comparable likelihood. One first solution is presented in Figure 6I. First of all asymmetric division for LGR5 is excluded as its inferred probability is zero (this holds also for the other found solution shown in Figure S7D). Interestingly, LGR5 mostly self-renew, consistent with their stem-cell nature, with a small probability of symmetric differentiation (i.e., generating two *KRT20*^+^ cells). Note that this is also consistent with the rare appearance in smFISH data of *KRT20*^+^ cells that are also *MKI67*^+^ which can be explained by the slow degradation of *MKI67* transcript in *KRT20*^+^ cells that recently divided.^71^^,^^72^ We also note that we did not assume prior information on the probability of back-differentiation. Interestingly, our inference results return very small (but non-zero) values for this transition on *KRT20*^+^ cells. The parameters inferred for *KRT20*^+^ cells show a marked decrease in the probability of death, which largely explains the probability of extinction. At 10 dps *KRT20*^+^ cells are instead found to be quiescent, coherently with their differentiation status. The results of inference show distribution of cell types and fraction of cell types per organoid compatible with those experimentally observed (Figures 6J and 6K). As anticipated, our results return another admissible solution compatible with data (Figures S7D–S7F), where *LGR5*^+^ cells decrease self-renewal in favor of symmetric differentiation, while *KRT20*^+^ mostly die out. While theoretically plausible, we excluded this solution because of the large amount of apoptotic events predicted, which is not frequently observed in growing organoids. In conclusion, our method proposes a number of experimentally testable predictions for the determination of active transitions and proposes quantitative estimates and phenotypic transitions to explain observed data.

## Discussion

In this paper, we address the problem of identifying existing phenotypic switches and their probability of occurrence within a population when cell tracking is not necessarily available. Our approach relies on the hypothesis that biological data are reasonably well described by a BGW branching process. BGW-based models allow to describe a diverse set of branching processes, they are suitable for different extensions, they allow analytical calculations and can be easily numerically simulated. Such aspects make our approach conceptually very clear and also easily generalizable to other contexts (one above all: epidemiological models^73^). In the context of cancer and cancer therapy, we showed how our approach can be used to infer phenotypic transitions in a multi-type tumor, and this can of course be applied to normal tissues as well. An interesting application of our method, outlined here, is that of identifying the mechanism of a drug or compound or growth factor on cell populations. It is indeed possible to compare cell populations under treatment and untreated and identify which transitions are mostly affected by the drug. We have shown that our inference approach is able to quantitatively determine all transition probabilities considered in a general model with no need to track single cells and, under certain conditions, even with the sole knowledge of endpoint counts. Such a feature is particularly relevant in the context of cancer phenotypic plasticity where lineage hierarchies might be different from those found in normal tissues and no strong prior might be available. This applies in particular to reversibility of—normally unidirectional—transitions, which can be determined quantitatively. The inference technique presented here exploits the statistical distribution of observed data without the need of measuring or monitoring directly each transition. When compared with the direct observation of a transition, the data employed within our approach is in a sense less informative. As with any inference problem, this comes at the price of the need of larger amount of data in order to perform statistically reliable estimates. We have shown that for a reasonable number of cell types (*M* ≤ 3), inference can be performed with a number of datapoints which is experimentally reasonable even with a flat prior on the parameters. When increasing the number of populations the number of data becomes significantly larger and might not be fully compatible with experimental needs, therefore potentially requiring to complement information with a non-flat prior. Note that an alternative to impose a non-flat prior is that to match the moments of the process from the data. Within the BGW formalism, this can be done by equating analytically derived moments with empirical values. Such procedure sets a number of additional constraints on the parameters, thereby reducing the amount of data required for inference. The proposed BGW branching processes involve essentially discrete states and discrete time. While more complicated phenotypic descriptions having continuous states cannot be described within this formulation, one advantageous aspect of the approach presented here is that stochasticity is taken into account in a very natural way. This means that extinction of clones is possible in the model even within clones that are growing on average. This feature cannot be considered directly in continuous models. A valid method to infer properties of phenotypic transitions was proposed by Klein et al.,^48^ followed by a number of many successful applications to experimental clonal analysis. Such formulation rather than being based on inference is relying on the process of fitting asymptotic size distribution through the dynamics reconstructed via a master equation. Typically, clonal dynamics is resolved by assuming one unique duplication time for both symmetric and asymmetric cell division, and the population model is postulated *a priori*. Within these limits, asymptotics presented by Klein et al.^48^ might be more reliable than the approach presented here for long observation times and large clones. Our approach is indeed based on a higher degree of synchrony and has quite the opposite behavior, with large asynchronous clones being more difficult to characterize. It is worth noting, however, that our modeling approach does not allow non-biological rapid cell doublings as would a Poisson distributed doubling time.

One piece of information that is required to apply our approach in the case of multi-type dynamics is the number of defined types that compose the population. While this is strictly necessary in the examples presented within the paper, we note that model selection approaches with varying number of populations could be applied in our proposed framework in order to identify the most adherent model to the presented data. Within our manuscript we have not explicitly described genetic heterogeneity. While our approach could be applied to single, genetically distinct, clones in order to describe potentially different, clone dependent, lineages, we have not included formally the possibility that one cell can accumulate a genetic mutation nor the interactions between different genotypes within a tumor. Our approach is ideally suited for relatively young populations making the probability of newly acquired mutations very small. It is worth noting however that interactions between different genetic species can be naturally introduced in our approach by describing genetically different cells as different BGW types. Therefore, provided that genetically distinct clones and their phenotypic states can be tracked simultaneously, our framework enables the integration of these multi-level interactions (with the computational limitations described).

We note that the formally correct way to consider birth-death processes with biologically realistic doubling time distribution and dynamics would be to choose a Bellman-Harris stochastic process rather than a BGW. This however comes with significant formal complications and will be left for future developments. It is indeed important to note that our MCEM method is not necessarily bound to BGW processes in particular and its use can be extended to other discrete branching processes or other theoretical models quite simply.

Another aspect to be discussed is the possibility to include cell fate correlations between sister cells, or the possibility of having one progenitor that can follow different types of symmetric and asymmetric cell divisions.^16^^,^^20^^,^^35^^,^^36^^,^^37^ The model introduced here includes the possibility for a single progenitor type to transition into any other states through symmetric or asymmetric cell divisions and also includes the possibility of transitions not explicitly linked to cell cycle. Such wide range of possibilities allows our approach to uncover previously unknown modes of tissue homeostasis and growth paradigms. As a side comment, throughout the experimental data considered here, cell death of both siblings (either synchronous or not) was anecdotally observed. Such scenario, which might appear difficult to consider in our approach, could indeed be included by introducing a new cell type which can be reached by symmetric cell division and which can only die with probability 1.

In conclusion, we presented a suite of inference methods to identify lineage transitions within a cell population. Our strategy is useful to dissect population hierarchies in growing and treated cell populations and gives access to dynamical features without requiring cell tracking but only cell counts and types. Future research will include the generalization of our approach to other types of branching processes in order to extend the applicability to an even more general set of biological problems.

### Limitations of the study

Discrete stochastic branching processes like BGW do not have clear notion of time and everything is contained within the concept of generation. When putting together inferred probabilities and trying to express these as rates this needs to be taken into account. Also, whenever dealing with experimental data, a number of pre-processing steps needs to be performed to align the data series and estimate the generation time. As written throughout the manuscript, our method works well for progenies that remain substantially correlated for several generations, while it is not suitable *a priori* for Poissonian or similar statistics. Indeed, as a general consideration, BGW branching processes are not suitable to describe large well-mixed cell populations as these would show completely asynchronous cell divisions. It is worth noting that in our simulations (STAR Methods), estimates based on inference with BGW on realistic data deviates from empirical values of around 10% which is reasonable in the logic of guiding experimental validation. As detailed previously, Bayesian methods rely on writing the full likelihood of a given observation and for many generations (or many observations or many cells). This translates into extremely long polynomials which are hardly manageable with currently available computing possibilities. Such situations can however easily be addressed with the MCEM method which does not need explicit likelihood knowledge. A feasible approach (left for future extensions) is to resort to truncated likelihoods. We note that the number of possible cell-types that our approach can handle heavily relies on the amount and type of data available (even with the MCEM method). In order to exploit a fully agnostic model one has to compensate with large amount of data, possibly incompatible with experimental approaches. Therefore, biological experimental settings describing dozens of clones each characterized by multiple cell-types cannot currently be addressed within our method.

We note also that BGW are normally spaceless models, where the notion of neighbors is in principle not included. In cases where spatial interactions are necessary this model would not be a natural choice.

Finally, we note that with increasing number of types, for a given observed data, the number of possible trees explaining that particular dataset increases, giving reduced identifiability, i.e., different sets of parameters might explain equally well the same set of observations. Analogously, specific, very rare, transitions might be difficult to observe, resulting in underestimated transition probabilities or reduced observability.

## Resource availability

### Lead contact

Requests for further information and resources should be directed to and will be fulfilled by the lead contact, Alberto Puliafito (alberto.puliafito@unito.it).

### Materials availability

This study did not generate new materials.

### Data and code availability

• The data presented in this paper will be shared by the lead contact upon reasonable request.
• Instances of the codes are available on GitHub at the following link: https://github.com/AndreaPiras21/Inference-Cells/ with the following DOI: https://doi.org/10.5281/zenodo.21276633.
• Any additional information required to reanalyze the data reported in this paper is available from the lead contact upon request.

## Acknowledgments

This work was supported by AIRC (10.13039/501100005010Associazione Italiana per la Ricerca sul Cancro) via grant MFAG-25040 to A. Puliafito, IG-23211 to L. Primo and 5x1000 grant 21091 to A.B.; FPRC ETS 5x1000, 10.13039/501100003196Ministero della Salute 2022 - Progetto CARESS to A. Puliafito; 10.13039/501100021856MUR (10.13039/100017336Dipartimenti di Eccellenza DM 11/05/2017 n262) to the Department of Oncology, 10.13039/501100006692University of Turin (2023-2027 14586 DIORAMA); 10.13039/501100003196Italian Ministry of Health, Ricerca Corrente 2025–2026; 10.13039/100018693European Union’s Horizon Europe research and innovation program under the Marie Skłodowska-Curie grant agreement no 101104340 to S.J.F.; Fondazione Veronesi 2023 postdoctoral fellowship to S.J.F. We acknowledge fruitful discussions with Livio Trusolino and the precious work of Dr. Roberta Porporato on smFISH.

## Author contributions

Conceptualization, A. Piras, A.C., and A. Puliafito; data curation, A. Piras and A. Puliafito; formal analysis, A. Piras, A.C., and A. Puliafito; funding acquisition, L. Primo and A. Puliafito; investigation, A. Piras, F.G., L. Pizzini, and S.J.F.; methodology, A. Piras and A.C.; project administration, A.C. and A. Puliafito; resources, L. Primo and A. Puliafito; software, A. Piras, A.C., and M.N.; supervision, A.C., L. Primo, and A. Puliafito; validation, all authors; visualization, A. Piras, L. Pizzini, A.C., and A. Puliafito; writing – original draft, A. Piras and A. Puliafito; writing – review and editing, all authors.

## Declaration of interests

The authors declare no competing interests.

## Declaration of generative AI and AI-assisted technologies in the writing process

During the preparation of this work the authors used LLMs in order to improve English syntax and to assemble plots with the package Matplotlib for Python. After using this tool/service, all authors reviewed and edited the content as needed and take full responsibility for the content of the published article.

## STAR★Methods

### Key resources table

**Table:** 

REAGENT or RESOURCE	SOURCE	IDENTIFIER
**Critical commercial assays**		

RNAScope Multiplex Fluorescent Reagent Kit v2	ACD/Bio-techne®	#323100
Hs-LGR5-C3	ACD/Bio-techne®	#311021-C3
Hs-KRT20-C2	ACD/Bio-techne®	#549681-C2
OPAL690	Aurogene	#FP1497001KT
OPAL570	Aurogene	#FP1488001KT

**Deposited data**		

Clonal data for hematopoietic cells	Wheat et al.^68^	https://github.com/justincwheat/Single-Molecule-Imaging-of-Transcription-Dynamics-in-Somatic-Stem-Cells
Timelapse of MDCK-II	Puliafito et al.^60^	N/A

**Experimental models: Cell lines**		

MG-63 Osteosarcoma cell line	Interlab Cell Line Collection (ICLC)	Accession number: ICLC HTL99003; RRID:CVCL_0426
Tumor organoid line derived from patient-derived xenograft CRC0322	Prof. Andrea Bertotti	Leto et al.^70^
MDCK-II	WJ Nelson, Stanford University	Puliafito et al.^60^

**Recombinant DNA**		

pLentiPGK DEST H2B-iRFP670	Markus Covert (via Addgene)	Addgene plasmid #90237; RRID:Addgene_90237
pBOB-EF1-FastFUCCI-Puro	Kevin Brindle and Duncan Jodrell (via Addgene)	Addgene plasmid #86849; RRID:Addgene_86849
pH2B-emiRFP670	Vladislav Verkhusha (via Addgene)	Addgene plasmid #136571; RRID:Addgene_136571

**Software and algorithms**		

Sample code for inferring lineage hierarchies in cell populations	This paper	https://github.com/AndreaPiras21/Inference-Cells/; https://doi.org/10.5281/zenodo.21276633

### Experimental model and study participant details

MG-63 cell line, an osteosarcoma cell line derived from a male donor, was purchased from Interlab Cell Line Collection (ICLC). The cell line was kept in stock in our institute’s cell culture facility, which re-authenticates cells by applying the PowerPlex 16 HS System (#DC2101, Promega, USA) and tests for Mycoplasma contamination with a PCR Mycoplasma Detection kit (#G238, Applied Biological Materials Inc., Canada).

MG-63 were cultured at 37 °C under 5% CO2 humidified atmosphere in DMEM low glucose (#ECM0070L, Euroclone S.p.A., Milano, Italy). Culture medium was supplemented with 10% fetal bovine serum (#A5256701, Gibco BRL, ThermoFisher, Waltham, MA, USA), 100U/mL of penicillin and 100μg/mL streptomycin (#ECB3001D, Euroclone S.p.A., Milano, Italy) and 2mM L-Glutamine (#ECB3000D-20, Euroclone S.p.A., Milano, Italy). Cells were cultured in standard tissue culture plastic dishes (Falcon) and imaging dedicated supports (#80826 μ-Slide 8 Well ibiTreat; #89626 - μ-Plate 96 Well Square, ibiTreat; IBIDI, GmbH, Gräfelfing, Germany). MG-63 cells transduced with lentiviral vectors indicated below were cultured at low density (125/well-96 Well Square or 500/well-8 well) on day 0, in 300 μL of complete growth medium. The next day, we performed live imaging choosing only single-cell-fields, with an inverted wide-field microscope Nikon Ti2 (Lipsi, Nikon Instruments, Amstelveen, The Netherlands) with a 20X 0.75 NA objective and a digital camera (IRIS 15; Photometrics, Tucson, AZ, USA) and an incubator to keep the plate stably at 37°C and 5% CO2. Images were taken every 20 minutes and the time-lapse assay lasted 96 hours. Plasmids were purchased from Addgene. pLentiPGK DEST H2B-iRFP670 was a gift from Markus Covert (Addgene plasmid # 90237 ^74^). pBOB-EF1-FastFUCCI-Puro was a gift from Kevin Brindle & Duncan Jodrell (Addgene plasmid # 86849 ^67^). The colorectal cancer tumoroid denoted by CRC0322, in our biobank^70^, was originally derived from a hepatic metastatic originated in the rectum. Sex of the patient was not available in the clinical data at the time of collection. Organoids were cultured embedded in Growth Factor Reduced (GFR) Matrigel (Corning). Growing media consisted of Dulbecco’s modified Eagle medium/F12 (DMEM/F12) supplemented with penicillin-streptomycin, 2 mM L-glutamine, 1 x B27 (Gibco), 1 x N-2 supplement (Gibco), 1 mM N-acetylcisteine and 20 ng/mL of human EGF (Sigma-Aldrich). Organoids were cultured at 37°C in a humidified atmosphere with 5% CO2, and were regularly tested for mycoplasma

Generation times were estimated by manually spotting consecutive cell divisions in confocal timelapse experiments from the same patient-derived tumoroid (CRC0322) infected with the construct pH2B-emiRFP670. Confocal stacks accommodating entire organoids (with slice thickness 2*μm*) were acquired on a Leica SP8 confocal microscope, equipped with a 63X (HC PL APO CS2 63x/1.40 OIL) objective, 37°C humidified chamber with 5% CO2 over 67 h every 30 min pH2B-emiRFP670 was a gift from Vladislav Verkhusha (Addgene plasmid # 136571^75^).

SmFISH was performed by using the RNAscope Multiplex Fluorescent v2 kit (Advanced Cell Diagnostic -ACD- Cat. # 323100) on slices of tumoroids. Briefly organoids were seeded as single cells, grown embedded in a basement membrane extract (Corning Matrigel, Cat. # CLS354230) for the specified time after seeding (6 dps and 10 dps). Organoids were then extracted from the hydrogel, embedded into agarose, fixed in 4% paraformaldehyde and sliced. mRNA expression of target genes was evaluated on FFPE cytoincluded 5 *μ*m-thick sections. Slices were processed to detect *LGR5* (probe code 311021-C3), *KRT20* (probe code 549681-C2), and *MKI67* (probe code 591771) transcripts. Sequences of target probes, preamplifier, amplifier, and label probe are proprietary (Advanced Cell Diagnostics, Hayward, CA). Briefly, after deparaffinization and peroxidase blocking, the samples were pretreated with boiling antigen retrieval for 15 min and with protease Plus for 30′ at 40°C. Then, the samples were incubated for 2 h at 40°C with appropriate mixed probes. The signals were amplified and finally detected with OPAL dyes (1:1500) according to the ACD protocol and assigning OPAL520 at channel 1 (*MKI67*), OPAL690 at channel 2 (*KRT20*) and OPAL570 at channel 3 (*LGR5*). Adjacent, 0.3 *μm* spaced z-stacks were acquired with an inverted wide-field microscope Nikon Ti2 (Lipsi) with a 63×1.2 NA immersion objective and a digital camera (IRIS 15) and collapsed onto 2D images with either maximum projection or Extended Depth of Focus (for fluorescence or bright-field respectively).

### Method details

#### Inference of cell transitions in a BGW branching process

We begin by defining what kind of data we aim at inferring from. As previously described the observations W can be thought in different experimentally relevant situations:

• When tracking is available, we assume we have access to cell identities and transitions at all times. In general, this means that W contains for each cell its type, the type of its predecessor and the transitions that each cell performed at the end of each generation. This is a matrix that for each generation contains a variable number of entries (the cells at each generation). As explained below, the properties of the BGW processes are such that the probability of observing a particular configuration does not depend on the particular tree that ends up in that configuration but rather from the number of different transitions observed. Therefore, in practice, W here consists of a vector $W=\left\{w_{1},\ldots ,w_{T}\right\}$ of integer numbers, each counting the transitions of each type.
• In the case of consecutive measurements, the data consists in a set of matrices **Y**, one for each experimental replicate. Each entry in the matrix *Y*_*mk*_ of size *M*×(*n*+1) represents the number of cells of phenotype *m* at a given generation *k.* Here by *n* we indicate the maximum number of generations followed in the data (*k*∈{0, …,*n*}). It is worth noting that each matrix represents the offspring of one or more cells but all cells are related by genealogical relations in a given matrix. Here $W=\left\{Y^{1},\ldots ,Y^{R}\right\}$ where *R* is the number of replicates or datasets collected.
• In connected endpoints measurements each matrix **Y** is an *M* × 2 matrix where *Y*_*m*1_ contains the zero-th generation and *Y*_*m*2_ contains the *n*-th generation. Here $W=\left\{Y^{1},\ldots ,Y^{R}\right\}$.
• In unconnected endpoint measurements, each observation is represented by a collection of *M* × 2 matrices, each containing generations 0,*k* for different values of *k* (for example *k*∈{0, …,*n*}) but there is no genealogical relation between cells at different generations, so that effectively one deals with several connected endpoint measurements obtained at different unrelated generations. Here $W=\left\{Y^{1}\left(0\right),\ldots ,Y^{R}\left(n\right)\right\}$

#### Modeling cell lineages as stochastic branching processes

In order to be able to infer transitional probabilities in a multi-type biological setting we frame our problem into the context of multi-type BGW branching processes.^49^ We consider *M* distinct types (equivalent to cell types or states if more reversible, see Glossary, Table S1), where individuals (or cells) of the same type are assumed to behave identically, while cells of different types may exhibit distinct behavior. Each cell of type *m* can generate a number of descendants (daughter cells) between 0 and 2, and the cell type of each daughter cell belongs to one of the *M* possible type, while descendants of a cell of type *m* can in general belong to two distinct types *m*′ and *m*″ (either coincident as in the case of symmetric cell divisions or not as in the case of asymmetric cell divisions, see Figure 1A). In terms of BGW formalisms, this translates into a random variable describing the offspring of each type *m* at the *n*-th generation that we indicate with ${\vec{X}}_{m}\left(n\right)=\left(X_{m,1}\left(n\right),\ldots ,X_{m,M}\left(n\right)\right)$. The components $X_{m,m^{\prime }}\left(n\right)$ represent the number of descendants of type *m*′ generated by type *m* at the *n*-th generation. For example a realization ${\vec{X}}_{1}\left(n\right)=\left(1,0,\ldots ,0\right)$ corresponds to a transition 1→1, where a cell of type 1 was inactive at that generation, ${\vec{X}}_{3}\left(n\right)=\left(0,\ldots ,0\right)$ corresponds to 3→∅, where a cell of type 3 died, and ${\vec{X}}_{2}\left(n\right)=\left(0,2,0,\ldots ,0\right)$ corresponds to 2→(2,2), i.e., a cell of type 2 performing cell division.

The population size at the (*n*+1)-th generation can be expressed in terms of the number *Y*_*m*_(*n*) of cells of each type *m* at the *n*-th generation:

(Equation 4) $$Y\left(n+1\right)=\sum_{m=1}^{M}\sum_{k=1}^{Y_{m}\left(n\right)}\sum_{m^{\prime }=1}^{M}X_{mm^{\prime }}^{\left(k\right)}\left(n\right)$$

where the index *k* runs on all the *Y*_*m*_(*n*) individuals of type *m* at the generation *n*. Note that $\sum_{m^{\prime }=1}^{M}X_{mm^{\prime }}^{\left(k\right)}\left(n\right)\leq 2$. In the framework of multi-type BGW model, for each type *m*, we can write the probabilities ${\vec{\theta }}_{m}=\left\{\theta_{m0},\ldots ,\theta_{mT}\right\}$ associated to each possible transition that an individual of cell type *m* can perform, where *T* is the total number of different offspring configurations as shown in Figure 1A. It is worth remembering that the ordering of the transitions is arbitrary in the *M*×*T* matrix Θ containing all the probabilities *θ*_*mj*_. The total number of transitions *T* for each phenotype *m* can be calculated as follows: the transitions shown in Figure 1A and I-III (i.e., cell death, inactivity and self-renewal) will be 3, then there will be 3(*M*-1) for those shown in Figure 1A and IV-VI and (*M*-2)(*M*-1)/2 for those in Figure 1A and VII. This gives *T*=(*M*+1)(*M*+2)/2. Each cell type is assumed to act independently and so we have a normalization condition $\sum_{j=1}^{T}\theta_{mj}=1$ on each parameters ${\vec{\theta }}_{m}$ governing the cell behavior, for a total of *M* constraints.

The probability generating function (PGF) of the vectors ${\vec{X}}_{m}$ is defined as:

(Equation 5) $$f_{m}\left(\vec{s}\right)=E\left[{\vec{s}}^{{\vec{X}}_{m}}\right]=\sum_{t=0}^{T}\theta_{mt}{\vec{s}}^{{\vec{\nu }}_{mt}}$$

where *s*_*m*_ are the auxiliary variables relative to each phenotype and are grouped in a vector $\vec{s}=\left\{s_{1},\ldots ,s_{M}\right\}$, and where we have used the following compact notation ${\vec{s}}^{{\vec{X}}_{m}}=s_{1}^{X_{m1}}\cdots s_{M}^{X_{mM}}$. The vector of exponents ${\vec{\nu }}_{mt}=\left(\nu_{mt1},\ldots ,\nu_{mtM}\right)$ contains the number of cells for each type that emerged from the transition *t*.

Given that the maximum number of offspring is 2, Equation 5 is a polynomial with at most degree 2. The PGFs of the offspring distribution $\vec{X}$ can be used to calculate the population distributions $\vec{Y}\left(n\right)=\left\{Y_{1}\left(n\right),\ldots ,Y_{M}\left(n\right)\right\}$ containing the number of individuals for each type at the *n*-th generation by recursive composition. By taking into account the properties of the PGFs, the probability to obtain a specific number of individuals for each type {*Y*_1_(*n*) = *y*_1_, …,*Y*_*M*_(*n*) = *y*_*M*_}, while starting with one individual of type *m* can be written as:

(Equation 6) $$p\left(\vec{Y}\left(n\right)\right)=\frac{1}{y_{1}!\ldots y_{M}!}\frac{\partial^{y_{1}}}{\partial s_{1}^{y_{1}}}\ldots \frac{\partial^{y_{M}}}{\partial s_{M}^{y_{M}}}f_{m}^{\left(n\right)}\left(\vec{s}\right){|}_{\vec{s}=0}$$

where $f_{m}^{\left(n\right)}$ is the recursive composition performed *n* times on *f*_*m*_, i.e.,:

(Equation 7) $$f_{m}^{\left(1\right)}\left(\vec{s}\right)=f_{m}\left(s_{1},\ldots ,s_{M}\right)$$

(Equation 8) $$f_{m}^{\left(2\right)}\left(\vec{s}\right)=f_{m}\left(f_{1}\left(\vec{s}\right),\ldots ,f_{M}\left(\vec{s}\right)\right)$$

(Equation 9) ⋮

(Equation 10) $$f_{m}^{\left(n\right)}\left(\vec{s}\right)=f_{m}\left(f_{1}\left(f_{1}\left(\ldots \right),\ldots ,f_{M}\left(\ldots \right)\right),\ldots ,f_{M}\left(f_{1}\left(\ldots \right),\ldots ,f_{M}\left(\ldots \right)\right)\right)$$

It is important to note that Equation 6 contains all possible trees, or combination of events, that connect a given initial configuration with one cell of phenotype *m* to the generic configuration $\vec{Y}\left(n\right)=\left(Y_{1}\left(n\right),\ldots ,Y_{M}\left(n\right)\right)$. A worked out example for 2 types is given below. The BGW model just introduced has obvious analogies with the context of cell lineages, where each type might be thought as a distinct phenotypic identity or cell type, characterized by a relatively stable transcriptional status. Transitions in this context identify all possible actions that each type can undergo. Cell death, inactivity and self-renewal for a given phenotype *m* are indicated in Figure 1A (I-III) and correspond to terms in $s_{m}^{0}$, $s_{m}^{1}$ and $s_{m}^{2}$ respectively in $f_{m}\left(\vec{s}\right)$. Note that a non-zero probability of inactivity for a given phenotype, while not changing the number of cells in the population, allows some degree of dynamic desynchronization that naturally appears in biological setups. A cell of a given phenotype *m* can also switch to a phenotype *m*′ as shown in Figure 1A IV to take into account extrinsic de/differentiations and would appear as terms in $s_{m}s_{m^{\prime }}$ in the corresponding PGF $f_{m}\left(\vec{s}\right)$. Cell divisions other than self-renewal, can either be symmetric (Figure 1A V) or asymmetric (Figure 1A (VI,VII)) where either one or none of the daughter cells is equal to the mother cell and these two terms would be of the form $s_{m^{\prime }}^{2}$, $s_{m}s_{m^{\prime }}$ or $s_{m^{\prime }}s_{m^{\prime \prime }}$ in the PGF. Average growth rates and moments of each type can be easily obtained from the PGF.^49^ In the proposed model the number of parameters *M*·*T* grows rapidly with the number of phenotypes with law *M*·*T* = *M*(*M*+1)(*M*+2)/2. Therefore, multi-type complicated population structures map into a relatively large set of interpretable parameters. It is worth noting that if the parameters are all constant, phenotypic transitions would be described by our model as just a stochastic event with an assigned probability. However, parameters can also be made a function of the number of cells in one or more types, making this model more suitable to describe interaction among cell types or secreted molecules.^33^ In this situation, in order to maintain markovianity of the process, one needs to have as many sets of parameters as the number of conditions or number of cells in a given phenotype. In what follows we show how to implement inference with such a model, by starting from observed data. It is the data in our approach that will select which transitions are compatible with observations and which not, determining the underlying hierarchical structure of the population. Including all possible transitions in the model means that inference is performed even with no prior knowledge on population dynamics. We developed 3 different approaches which give access to different levels of information: analytical inference, EGS and Monte Carlo Expectation Maximization. Analytical inference is strictly Bayesian, while the Extended Gibbs Sampler (EGS) is a numerical method. Both these approaches output the full probability distribution functions for the parameters (exact and approximated respectively) in situations where we can computationally afford to write the likelihood (small amount of data/low number of cells or sparse data). A third method is presented (the Monte Carlo Expectation Maximization, MCEM) which outputs only point estimates for the parameters and is suited to cases where the amount of data is high and/or the data are sparse, i.e., we do not necessarily have information about intermediate population sizes.

#### Analytical Bayesian inference on BGW branching processes

Bayesian inference applied to BGW branching processes allows one to infer the posterior distribution for the model parameters starting from the empirical observation of single or multiple trees.^50^^,^^51^^,^^76^^,^^77^^,^^78^ What follows is written as referring to consecutive observations without tracking but can be extended to any of the observations W presented in the previous section. We use the letter **Y** to indicate the whole data in W. Note that while here we derive the approach on the entire dataset at once, one can apply the following formalism for one instance of **Y** at a time, and update the prior at each iteration with the posterior. This approach is followed in the manuscript only to explicitly show the convergence process while adding data but is not computationally efficient. For now the set of parameters Θ is thought as being the same across all datasets which are essentially different observations or realizations of the underlying BGW process. In this case we can start from Bayes’ rule:

(Equation 11) $$p\left(\Theta |Y\right)=\frac{L\left(Y|\Theta \right)p\left(\Theta \right)}{p\left(Y\right)}$$

where we are relating the probability distribution of the parameters given the observed data *p*(Θ|**Y**) with the prior belief on parameters *p*(Θ) and the probability of observing the specific data given a certain value of the parameters (or likelihood) L(Y|Θ). The latter, within our model, is found through the PGF. We can indeed decompose the likelihood using two distinct assumptions: independence and markovianity. Different experiments/observations are assumed to be statistically independent, and the number of cells at the (*n*+1)-th generation depends only on the number of cells at the *n*-th generation. In this way we can write the likelihood as a product of the probabilities of each independent observation, which can be easily derived from Equation 6. The term *p*(Θ) on the numerator of the r.h.s. of Equation 11 incorporates our belief on the parameters. Among the possible choices we assume a flat prior. This assumption can however be updated with a different and more stringent belief with the immediate effect of decreasing the number of free parameters in Equation 11. The denominator on the r.h.s. of Equation 11 is known as the marginal likelihood (or evidence) and can be written as

(Equation 12) $$P\left(Y\right)=\int L\left(Y|\Theta \right)p\left(\Theta \right)d\Theta =:E_{p\left(\Theta \right)}\left[L\left(Y|\Theta \right)\right]\text{,}$$

where the integration is taken over the whole parameter space, taking into account the normalization condition. Equation 11 leads us to the posterior *p*(Θ|**Y**), which is the probability distribution of the parameters of our BGW model given the observed data. This is a multivariate function which needs to be marginalized to find the probability distribution *p*(*θ*_*mj*_|**Y**) for each single parameter. Point estimators like the mean and the variance of the distribution can be easily calculated starting from the marginal distribution above.

The analytical approach here presented can be employed in several experimentally relevant situations, with a number of limitations mostly originating from computational costs. A first interesting case is that of cell tracking: in the logic of being able to track all cell transitions the likelihood for each observation is essentially that of a particular tree, and the likelihood of all independent observations just the product of all likelihoods. Inferring parameters in this situation is therefore conceptually simple as applying Equation 11 as is. Another interesting case would be that of having the counts for each type, or the total number of cells at each generation. In this case one would construct a likelihood with the conditioned probabilities and again apply directly Bayes’ formula. Finally, this approach could also be applied when one only has information regarding the count of individuals of each type on non-consecutive generations, a situation typically encountered when dealing with fixed samples at a given time. In this case the likelihood would contain all possible trees to reach a given configuration. A plot of an example of a full posterior is shown in Figure S2. The numerical implementation of the presented approach is described below and a sketch is given in Figure S1A.

One severe limitation of the proposed analytical inference is that computational cost becomes rapidly unpractical when increasing either the number of data, the number of cells or the number of cell types and hence the number of parameters. In such situations the likelihood becomes a significantly long list of monomials that while trivial to integrate constitute a computationally prohibitive challenge on standard computers. Analogously, if the data only contains information on a late number of generations, the number of trees to satisfy the observation can grow quite rapidly and become computationally intractable. Therefore, alternative methods are needed, based on numerical simulations that possibly avoid the explicit calculation of the marginal likelihood.

#### The knowledge of prior information

The simplest way of using prior experimental information is that of modifying the model in a way that it contains a reduced number of parameters. This is the case for example of when one cell type is known to be post-mitotic, or terminally differentiated. In this case, parameters corresponding to the unobserved transitions can be eliminated from the model used inference. It is worth noting that should the experimentalist then observe one such transitions, this would not be compatible with the model and would make the model unable to explain it.

Another typical situation is that of knowing that some transitions are much more frequent than others. For example one might know that cell division of type A is occurring on a faster scale than for type B. While choosing a formal way to implement this information into a prior might not be easily generalizable, this kind of knowledge can also be used within our framework and can help inference converge faster to the solution.

#### Applicability to single cell experimental techniques

Emerging technologies combine single cell transcriptomics with barcoding so that clonal identity (i.e., sharing a common ancestor) can be integrated with cell type or state.^79^^,^^80^^,^^81^ Such experimental possibility is clearly making lineage reconstruction more feasible. We would like to note that, currently, our approach is not optimal for barcoding experiments due to the following limitations.

• In order for our inference method to be effective in determining transitions, the number of elapsed generations between the initial and the final state needs to be small. Indeed, when the number of elapsed generations is large, the number of compatible trees with initial and final configuration becomes increasingly large thus keeping uncertainty on the inferred parameters too large (especially if the number of types or states is large). This limits the applicability of our methods to short experiments which however suffer even more of technical and experimental issues. Indeed the barcodes are normally to be amplified in order to obtain a significant yield in terms of RNA to be sequenced and to obtain unique barcodes to be split into distinct experimental conditions.
• Currently, single cell transcriptomics can reasonably aim at 20K cells. These cells however, due to experimental procedures, would come from multiple culture dishes or clones and therefore would likely constitute several incomplete clones, i.e., a few cells would share the same barcode but not all cells with the same barcode would be detected. This might introduce biases in determining the phenotypic transitions and would require some kind of sampling based data generation. While theoretically this is not impossible to address within our framework, it represents a significant complication and makes our approach not ideal in this context.
• Matching barcodes with initial type or state is not possible currently, as cells need to be sacrificed in order to extract phenotypic information. Therefore one could only reason in probabilistic terms unless prior information is available (for example, all starting cells are of one type only). Barcodes from initial and final states would in fact have to match, and dropout rates for barcodes are reported to be significant (reportedly in the range 30%–60% according to ref.^81^). In the ideal situation one would expect to have 20K unique barcodes in the initial state and 20K cells from mixed barcodes (presumably not completely overlapping).

Complementary information on lineage reconstruction and particularly on transitions between different states comes from trajectory inference methods in single cell transcriptomics data. Such approaches rely on collective measurements, where clonal relations, i.e., relations to ancestors, are inevitably lost. Such approaches could definitely represent a very valuable approach to build prior information that can be implemented within the inference method.

#### Worked-out likelihoods for BGW branching process with 2 cell types

We here explicitly develop the likelihood of observing a set of particular realizations as indicated in Figure S8.

The PGFs in this case can be written as:

(Equation 13) $$f_{1}\left(s_{1},s_{2}\right)=\theta_{11}s_{1}^{2}+\theta_{12}s_{2}^{2}$$

(Equation 14) $$f_{2}\left(s_{1},s_{2}\right)=\theta_{21}+\theta_{22}s_{2}^{2}$$

In case ➀ (see Figure S8) we sought to determine the probability of observing one black cell dividing into two black cells which then respectively divide into two black cells and two white cells. This can be computed starting from the PGF as:

(Equation 15) $$p\left(y_{1}\left(2\right)=2;y_{2}\left(2\right)=2\right)=\frac{1}{4}\frac{\partial^{2}}{\partial s_{1}^{2}}\frac{\partial^{2}}{\partial s_{2}^{2}}f_{1}\left(f_{1}\left(s_{1},s_{2}\right),f_{2}\left(s_{1},s_{2}\right)\right){|}_{\vec{s}=0}=2\theta_{11}^{2}\theta_{12}$$

Analogously, case ➁ represents the tree where one black cell divides into two white cells which then both standby. This can be worked out as:

$$p\left(y_{1}\left(2\right)=0;y_{2}\left(2\right)=2\right)=\frac{1}{2}\frac{\partial^{2}}{\partial s_{2}^{2}}f_{1}\left(f_{1}\left(s_{1},s_{2}\right),f_{2}\left(s_{1},s_{2}\right)\right){|}_{\vec{s}=0}$$

(Equation 16) $$=\frac{1}{2}\frac{\partial^{2}}{\partial s_{2}^{2}}\left[\theta_{11}{\left(\theta_{11}s_{1}^{2}+\theta_{12}s_{2}^{2}\right)}^{2}+\theta_{12}{\left(\theta_{21}+\theta_{22}s_{2}\right)}^{2}\right]{|}_{\vec{s}=0}=\theta_{12}\theta_{22}^{2}$$

Case ➂ corresponds to one black cell dividing into two white cells then both dying:

(Equation 17) $$p\left(y_{1}\left(2\right)=0;y_{2}\left(2\right)=0\right)=f_{1}\left(f_{1}\left(s_{1},s_{2}\right),f_{2}\left(s_{1},s_{2}\right)\right){|}_{\vec{s}=0}=\theta_{12}\theta_{21}^{2}$$

A slightly more complex case is ➃ which corresponds to the probability of having 4 cells of any type at generation 2. This last case corresponds to three independent possible trees, whose probabilities add up:

$$p\left(y_{1}+y_{2}=4\right)=\left(\frac{1}{4!}\frac{\partial^{4}}{\partial s_{1}^{4}}+\frac{1}{4}\frac{\partial^{2}}{\partial s_{1}^{2}}\frac{\partial^{2}}{\partial s_{2}^{2}}+\frac{1}{4!}\frac{\partial^{4}}{\partial s_{2}^{4}}\right)f_{1}\left(f_{1}\left(s_{1},s_{2}\right),f_{2}\left(s_{1},s_{2}\right)\right){|}_{\vec{s}=0}$$

(Equation 18) $$=\theta_{11}^{3}+2\theta_{11}^{2}\theta_{12}+\theta_{11}\theta_{12}^{2}$$

#### Worked-out likelihoods for different data types in a BGW branching process with 1 cell type

Here, we explicitly consider an example of inference starting from different types of available data. Suppose we are monitoring two generations of a single-type BGW process with cell death, standby and division indicated by probabilities *θ*_0_,*θ*_1_,*θ*_2_ as usual. We consider: i) the full tracking case, where we observe one division at first and then two inactivations, ii) the consecutive measurements case where we observe the number of cells at any generation (here: one, two and two), iii) the case where we only observe the initial and final number of cells.

The tracking case is represented by the following likelihood:

(Equation 19) $$L=\theta_{1}^{2}\theta_{2}$$

This corresponds to a single tree where one cell divides first and then the two daughter cells perform a standby. The marginal posteriors correspondingly indicate a larger probability for inactivation and division, while death probability peaks on zero.

The consecutive case can be worked out by multiplying the probabilities of observing each transition (1→2 and 2→2), to obtain:

(Equation 20) $$L=\theta_{1}^{2}\theta_{2}+2\theta_{0}\theta_{2}^{2}$$

where now there are two possible distinct independent trees one in which there is adivision followed by two standby, and the second where one of the daughter cells divides and the other dies (the factor two follows from the exchangeability of the two cells).

As a final case we consider the case where the configuration at the intermediate generation is not observed. The likelihood can be calculated as previously:

(Equation 21) $$L=\theta_{1}^{2}\theta_{2}+2\theta_{0}\theta_{2}^{2}+\theta_{1}\theta_{2}$$

where we have now a a contribution from a third tree which corresponds to one cell standing by and then dividing.

We remark that in these three cases the same underlying process yields different inference results as shown in the marginals in Figure S9. Note that the maximum of the marginals for parameters *θ*_0_ and *θ*_1_ is inverted when switching from tracking to consecutive measurements.

#### Approximate Bayesian inference with the extended Gibbs sampler

The explicit knowledge of the analytical likelihood allows one to extend previous work,^78^ belonging to the class of Monte-Carlo Markov-Chain algorithm, to the more general case of inferring the parameters of a multi-type BGW process without the need of generation-contiguous observations. The approach developed here, can be applied to any dataset W with the exception of the case with tracking which has no reason to be tackled with this method (see ref.^76^ for an extension toward non-parametric distributions). We use the letter **Y** to indicate the data in W (without tracking).

As explained at the end of the previous section, a problem of the analytical setup is that when the number of cells is large, for any given dataset W (without tracking information), there are multiple trees that end up in the same configuration, resulting in a likelihood which is the sum of several terms each corresponding to one of these trees. Each of these trees can be indicated by a matrix **Z** containing the count of all the possible *Z*_*mt*_ transitions for each phenotype *m*, where *m* = 1, …,*M* and *t* = 1, …,*T*. The exact knowledge of the tree **Z** determining the configuration **Y** with a given initial condition requires tracking each cell at all times. However **Z** can also be considered a latent variable, and this assumption still makes it possible to express the likelihood:

(Equation 22) $$L\left(Y|\Theta \right)=\sum_{Z\in \left\{Z^{\prime }\right\}}p\left(Y|Z\right)p\left(Z|\Theta \right)$$

where the summation is over the set of possible trees {*Z*′} compatible with the observation **Y**. The idea behind the Gibbs Sampler^78^ is to sample directly from the posterior distribution, knowing that it is proportional to the likelihood via the prior:

(Equation 23) $$p\left(\Theta |Y\right)\propto \sum_{Z\in \left\{Z^{\prime }\right\}}p\left(Y|Z\right)p\left(Z|\Theta \right)p\left(\Theta \right)$$

To sample from the posterior distribution one can sample a first parameter matrix Θ∗ drawing from the prior distribution. If all types are independent the prior can be factorized into marginal priors over each phenotype, and each marginal $p\left({\vec{\theta }}_{m}\right)$ can be expressed in terms of a Dirichlet distribution:

(Equation 24) $$p\left(\Theta \right)\propto \prod_{m=1}^{M}\prod_{t=0}^{T}\theta_{mt}^{n_{mt}-1}$$

where *n*_*mt*_ reflects the shape of the chosen prior distribution.

At this point, without tracking information, one needs to sample among all the possible trees that are compatible with the observation **Y**, which is formally done in Equation 23. Such information however is contained within the analytical likelihood, which lists all the different trees compatible with the observation as a sum of terms. Therefore the likelihood itself can be used to sample among all the trees as follows. Each monomial of the likelihood corresponds to a possible tree **Z**. We can therefore construct a vector by substituting the values Θ∗ into each monomial of the likelihood. Upon normalization, such a vector gives the actual probability of occurrence of each tree *p*(**Z**|Θ) and can be used to sample trees. At this point it is sufficient to exploit the fact that a Dirichlet distribution and the product of multinomial distributions are conjugated to find an instance of the posterior:

(Equation 25) $$p\left(\Theta |Y\right)\propto \prod_{m=1}^{M}\prod_{t=0}^{T}\theta_{mt}^{n_{mt}-1+Z_{mt}}$$

This posterior distribution can finally be used to sample a new set of values Θ∗.

The iterative use of this procedure produces a sequence of values {Θ∗} which distribution is known to converge to the marginal distribution of the true parameter values. A sketch of this approach is presented in Figure S1B, and the comparison with the analytical method is presented in Figure S3. It is worth noting that this approach generalizes that presented in ref.^78^, which only allows one to infer from data series containing generation-contiguous observations, i.e., *Y*(1), …,*Y*(*n*).

While the proposed version outperforms the analytical methods in a number of situations, it still relies on the ability of writing and calculating an exact likelihood function, which can be prohibitive in a similar set of cases as the previous method, making the use of this method suitable for intermediate number of cells and observations. Both the analytical method and the extended Gibbs sampler (EGS) suffer computational regimes that increase the exponents in the likelihoods (i.e., a higher number of cells and a higher number of observations) or those that increase the number of terms in the likelihood such as incomplete or non contiguous observations. A phase portrait containing these limitations is shown in Figure 1C. Such considerations call for yet another approach with better computational performances.

#### Inferring maximum likelihood estimators with Monte Carlo Expectation Maximization

In this section we limit our attention to connected non-consecutive observations (indicated as usual with **Y** which represents data in W). In this scenario, the likelihood contains a possibly very large number of possible trees or forests that are all compatible with the observed data. Given these challenges, it is computationally very difficult to compute the posterior distribution for the parameters with the previously described approaches. Therefore, we developed a Monte Carlo Expectation-Maximization algorithm (MCEM) to efficiently estimate the parameters by maximizing the log likelihood. If *y* is one particular observation within the set **Y**, the set of parameters $\hat{\Theta }$ maximizing the log likelihood is:

(Equation 26) $$\hat{\Theta }=\arg\max_{\Theta }\sum_{y}\mu \left(y\right)\log\;L\left(Y=y|\Theta \right)$$

where *μ* is the empirical distribution:

(Equation 27) $$\mu =\frac{1}{N}\sum_{y}I\left(Y=y\right)$$

and *I* is an indicator function.

As with the EGS, we write everything in terms of the hidden variables **Z**, containing all possible ways to go from an initial population to a given population at a future generation:

(Equation 28) $$\hat{\Theta }=\arg\max_{\Theta }\sum_{y}\mu \left(y\right)\log\sum_{Z\in \left\{Z^{\prime }\right\}}p\left(Y|Z\right)p\left(Z|\Theta \right)$$

This problem does not have a closed form solution. However the MCEM algorithm can be used to maximize the likelihood iteratively, by means of a surrogate function that possesses the same extrema of the likelihood. A surrogate function B(Θ) can be found exploiting Jensen’s inequality: *log*(*E*_*p*(*x*)_[*f*(*x*)])≥*E*_*p*(*x*)_[*logf*(*x*)] and can be written as:

(Equation 29) $$B\left(\Theta \right)=\sum_{y,Z\in \left\{Z^{\prime }\right\}}\mu \left(y\right)p\left(Z|y,\Theta \right)\log\frac{p\left(Y|Z\right)p\left(Z|\Theta \right)}{p\left(Z|y,\Theta \right)}$$

where the terms $p\left(Z|\vec{y},\Theta \right)$ are usually called responsibilities and can be written as $p\left(Z|\vec{y},\Theta \right)=p\left(\vec{y}|Z\right)p\left(Z|\Theta \right)/p\left(\vec{y}|\Theta \right)$.

In our multi-type BGW the term *p*(**Z**|Θ) can be written as:

(Equation 30) $$p\left(Z|\Theta \right)\propto \prod_{m=1}^{M}\prod_{t=0}^{T}\theta_{mt}^{z_{mt}}$$

The maximization of B(Θ) can be obtained with the method of Lagrangian multipliers with *M* constrains. This procedure lead us to maximum-likelihood estimation for each parameter

(Equation 31) $${\hat{\theta }}_{mt}=\frac{\sum_{\vec{y}}\mu \left(\vec{y}\right)\sum_{Z}p\left(Z|\vec{y},\Theta \right)z_{mt}}{\sum_{t=0}^{T}\sum_{\vec{y}}\mu \left(\vec{y}\right)\sum_{Z}p\left(Z|\vec{y},\Theta \right)z_{mt}}$$

The terms in the numerator required for the conditional probabilities can be theoretically calculated optimizing the surrogate function B(Θ) with respect to the responsibilities. However, it is possible to overcome this procedure by estimating the numerator by means of more efficient numerical methods. The previous numerator is approximated by the quantity:

(Equation 32) $${\hat{Q}}_{mt}=\frac{1}{L}\sum_{l=1}^{L}z_{ml}^{\left(l\right)}\tau_{l}\left(Y^{\left(l\right)}=\vec{y}\right)\mu \left(Y^{\left(l\right)}=\vec{y}\right)$$

where the average is performed over *L* different numerical simulations and $\tau_{l}\left(Y^{\left(l\right)}=\vec{y}\right)$ represents inter-arrival time between two identical observations defined mathematically as

(Equation 33) $$\tau_{l+1}\left(\vec{y}\right)=\tau_{l}\left(\vec{y}\right)I\left(Y^{\left(l\right)}\neq \vec{y}\right)+1$$

with initial condition $\tau_{1}\left(\vec{y}\right)=1$. With this estimators, each parameter *θ*_*mt*_ can be estimated through the formula

(Equation 34) $${\hat{\theta }}_{mt}=\frac{{\hat{Q}}_{mt}}{\sum_{t^{\prime }}{\hat{Q}}_{mt^{\prime }}}$$

This numerical approach is computationally effective even for relatively large number of cells and parameters and covers a wide range of experimentally relevant situations as shown in the next sections. Such efficiency however comes at the price of sacrificing the entire probability distribution function in favor of point estimators $\hat{\Theta }$. A graphical sketch for this method is shown in Figure S1C. Also, note that the method proposed here can be easily extended to the case of different initial cell numbers or types. The extended version is discussed in the STAR Methods.

#### Implementation of analytical Bayesian inference with Mathematica

In order to perform analytical Bayesian inference we made use of the software Wolframe Mathematica 13.2. Given an observation **Y** we first generate the likelihood L(Y|Θ) using the PGF of the model considered. The aim is then to calculate the posterior using Bayes’ formula:

(Equation 35) $$p\left(\Theta |Y\right)=\frac{L\left(Y|\Theta \right)p_{0}\left(\Theta \right)}{p\left(Y\right)}$$

where the prior *p*_0_ is taken to be flat, i.e., constant. The non trivial step here is to calculate the term in the denominator, *p*(**Y**), which can be written as

(Equation 36) $$p\left(Y\right)=\int_{\Theta }L\left(Y|\Theta \right)p_{0}\left(\Theta \right)d\Theta$$

where the integration is performed over the entire parameter space and takes into account all the *M* constrains. For the simplest case of one and only phenotype *M* = 1 this formula reduces to:

(Equation 37) $$p\left(Y\right)=\int_{0}^{1}\int_{0}^{1-\theta_{1}}p\left(\theta_{1},\theta_{2}\right)L\left(Y|\theta_{1},\theta_{2}\right)d\theta_{1}d\theta_{2}$$

where the integration is performed over only two parameters thanks to the constraint *θ*_0_ = 1-*θ*_1_-*θ*_2_.

It is worth noting that the likelihood for a general BGW process contains only polynomial terms and is therefore theoretically straightforward to integrate analytically. However, when the number of observations becomes large or there are many possible trees that explain one given observation, it can contain a significantly large number of terms and represents therefore one of the most computationally intensive tasks when implementing Equation 35.

Note that this approach can be taken iteratively or in one step. When computing iteratively one basically repeats the calculation in Equation 35 for each observation separately using the posterior at step *n* as the prior for step *n*+1. Alternatively one can calculate the full likelihood of all the observations at once and perform a one step calculation.

Once the posterior is obtained we can calculate the marginal distributions by integrating over all the parameters but one. The moments of the marginal distributions are also readily calculated starting from the marginal PDF. Two sample Mathematica scripts covering the cases of one type and two types can be found on our dedicated github: https://github.com/AndreaPiras21/Inference-Cells/, accessible at the following DOI: https://doi.org/10.5281/zenodo.21276633.

#### Implementation of numerical inference: The extended Gibbs sampler

The logic of a Gibbs sampler algorithm is to iteratively draw values from fully conditional distributions. In our setting, the fully conditional distribution $p\left(Z|\vec{y},\Theta^{*}\right)$ is provided directly by the analytical likelihood. Indeed, we can draw a particular set of trees by exploiting the fact that the likelihood contains all possible admissible sets of trees linking two separate generations that agree with the initial condition and the final observation. The choice of the tree set depends on the current parameter values $\hat{\Theta }$, which are initially drawn from a flat prior and subsequently drawn from a set of Dirichlet distributions, –one for each phenotype– updated based on the previously selected set of trees, $p\left(\Theta |Z^{*},\vec{y}\right)$.

The obtained series of drawn $\hat{\Theta }$ builds our approximated numerical posterior distribution, and each value ${\hat{\theta }}_{mt}$ can be exploited to build the approximated marginal distribution of the transition *j* with respect to phenotype *m*. A numerical implementation of this method in Mathematica can be found on our dedicated github: https://github.com/AndreaPiras21/Inference-Cells/ accessible at the following DOI: https://doi.org/10.5281/zenodo.21276633.

#### Implementation of numerical inference: Monte Carlo Expectation Maximization

The Monte Carlo Expectation-Maximization (MCEM) algorithm estimates transition probabilities in a BGW process by simulating cell lineage trees. These trees, which represent the full developmental history of a cell population, are typically not observed in experiments and thus constitute latent variables. However, they can be relatively easily simulated using a BGW process. Starting from an initial set of parameter values $\hat{\Theta }$, the algorithm simulates multiple cell lineage trees. For each simulation *l*, it records how many times a specific transition *t* occurs for a given phenotype *m*. At the same time, it also tracks the number of steps elapsed between two identical realizations (Equation 33). The inverse of the mean of this quantity becomes essentially the probability of observing that particular realization. This inferred probability is then combined with the empirical frequency of the realization in the observed dataset. Together, these quantities are used to update the parameter estimates $\hat{\Theta }$. The update is performed through the empirical mean ${\hat{Q}}_{mt}$ in Equation 32 which is iteratively updated while samples are generated using the following update rule:

(Equation 38) $${\hat{Q}}_{mt}\leftarrow {\hat{Q}}_{mt}+\frac{1}{l}\left(Z^{\left(l\right)}\tau_{l}\left(Y^{\left(l\right)}\right)\mu \left(Y^{\left(l\right)}\right)-{\hat{Q}}_{mt}\right)$$

Finally, the updated parameters are obtained using Equation 34, and the entire process is iterated until the estimates converge to a local maximum. A Python script implementing MCEM for one type BGW process can be found on our dedicated github: https://github.com/AndreaPiras21/Inference-Cells/ accessible at the following DOI: https://doi.org/10.5281/zenodo.21276633.

#### MCEM convergence

A known limitation of MCEM methods is that they are local: when the likelihood function has multiple extrema, the algorithm may converge to a suboptimal solution corresponding to a local maximum rather than the global one. To mitigate this issue, one can run the algorithm multiple times with different initializations of the parameters, compute the likelihood at each MCEM step, and select the estimates corresponding to the highest likelihood found. An example is shown in Figure S14, where the algorithm converges to two distinct sets of parameter values (corresponding to two different values of the likelihood). Computing the likelihood values (as shown in Figure S14) allows one to rank the quality of estimates, even when the true parameter values are unknown. It is worth noting that, given a certain data realization, multiple solutions with numerically equivalent (or nearly equivalent) likelihoods might exist. This corresponds to the notion that especially when the model is rich and contains many parameters, there exist different solutions that can explain data. While it is possible to verify which solution is biologically more acceptable or more compatible with complementary information, the most reliable way to overcome this difficulty is that of increasing the amount of data and/or priors used for inference.

#### Monte Carlo Expectation Maximization with variable initial number of cells

The MCEM algorithm presented in the main text implicitly assumes that all observations at the *n*-th generation originate from the same initial number of cells. Here, we generalize the approach to accommodate observations derived from different initial cell counts.

We consider an initial number of cells denoted by **Y**_**i**_ and end up with a final number of cells **Y**_**f**_. Now, the likelihood explicitly contains the dependence from the initial population **Y**_**i**_:

(Equation 39) $$L\left(Y_{f}|Y_{i},\Theta \right)=\sum_{Z\in \left\{Z^{\prime }\right\}}p\left(Y_{f}|Y_{i},Z\right)p\left(Z|Y_{i},\Theta \right)$$

When we perform the maximum-likelihood approach we need to sum also over the different initial conditions:

(Equation 40) $$\hat{\Theta }=\arg\max_{\Theta }\sum_{{\vec{y}}_{f}}\sum_{{\vec{y}}_{i}}\mu \left({\vec{y}}_{f},{\vec{y}}_{i}\right)\log\sum_{Z\in \left\{Z^{\prime }\right\}}p\left(Y_{f}|Y_{i},Z\right)p\left(Z|Y_{i},\Theta \right)$$

where $\mu \left({\vec{y}}_{f},{\vec{y}}_{i}\right)$ is the joint empirical distribution that can be written as:

(Equation 41) $$\mu \left({\vec{y}}_{f},{\vec{y}}_{i}\right)=\mu \left({\vec{y}}_{f}|{\vec{y}}_{i}\right)\mu \left({\vec{y}}_{i}\right)=\frac{1}{N_{{\vec{y}}_{i}}}\sum_{{\vec{y}}_{f}}I\left(Y_{f}={\vec{y}}_{f}|Y_{i}={\vec{y}}_{i}\right)\frac{1}{N}\sum_{{\vec{y}}_{i}}I\left(Y_{i}={\vec{y}}_{i}\right)$$

with $N_{{\vec{y}}_{i}}$ representing the number of observations starting with the initial population ${\vec{y}}_{i}$. Then we can write the surrogate function

(Equation 42) $$B\left(\Theta \right)=\sum_{\vec{y_{f}},\vec{y_{i}},Z\in \left\{Z^{\prime }\right\}}\mu \left({\vec{y}}_{f},{\vec{y}}_{i}\right)p\left(Z|{\vec{y}}_{f},{\vec{y}}_{i},\Theta \right)\log\frac{p\left(Y_{f}|Y_{i},Z\right)p\left(Z|Y_{i},\Theta \right)}{p\left(Z|{\vec{y}}_{f},{\vec{y}}_{i},\Theta \right)}$$

The maximum-likelihood estimate of each parameter becomes:

(Equation 43) $${\hat{\theta }}_{mt}=\frac{\sum_{{\vec{y}}_{f}}\sum_{{\vec{y}}_{i}}\mu \left({\vec{y}}_{i},{\vec{y}}_{f}\right)\sum_{Z}p\left(Z|{\vec{y}}_{f},{\vec{y}}_{i},\Theta \right)z_{mt}}{\sum_{t=0}^{T}\sum_{{\vec{y}}_{f}}\sum_{{\vec{y}}_{i}}\mu \left({\vec{y}}_{i},{\vec{y}}_{f}\right)\sum_{Z}p\left(Z|{\vec{y}}_{f},{\vec{y}}_{i},\Theta \right)z_{mt}}$$

Equation 23 in the main text is therefore replaced by:

(Equation 44) $${\hat{Q}}_{mt}=\frac{1}{L}\sum_{l=1}^{L}z_{ml}^{\left(l\right)}\tau_{l}\left(Y_{f}^{\left(l\right)}={\vec{y}}_{f}|Y_{i}^{\left(l\right)}={\vec{y}}_{i}\right)\mu \left(Y_{f}^{\left(l\right)}={\vec{y}}_{f}|Y_{i}^{\left(l\right)}={\vec{y}}_{i}\right)\mu \left(Y_{i}^{\left(l\right)}={\vec{y}}_{i}\right)$$

where the average is performed over different *L* simulations each indicated by *l*, and $\tau_{l}\left(Y_{f}^{\left(l\right)}={\vec{y}}_{f}|Y_{i}^{\left(l\right)}={\vec{y}}_{i}\right)$ represents inter-arrival time between two identical observations that start from the same initial condition:

(Equation 45) $$\tau_{l+1}\left({\vec{y}}_{f}|{\vec{y}}_{i}\right)=\tau_{l}\left({\vec{y}}_{f}|{\vec{y}}_{i}\right)I\left(Y_{f}^{\left(l\right)}\neq {\vec{y}}_{f}|Y_{i}^{\left(l\right)}={\vec{y}}_{i}\right)+1$$

with initial condition $\tau_{l}\left({\vec{y}}_{f}|{\vec{y}}_{i}\right)=1$. With this estimator, each parameter *θ*_*mt*_ can be estimated as before through the formula

(Equation 46) $${\hat{\theta }}_{mt}=\frac{{\hat{Q}}_{mt}}{\sum_{t^{\prime }}{\hat{Q}}_{mt^{\prime }}}$$

This formulation generalizes the one presented in the main text and is needed for example in the case of multiple phenotypes with feedback, where there data series can start from different initial conditions, or in the case of experimental data.

#### Initialization strategies for MCEM inference

As previously pointed out, MCEM only guarantees convergence to local extreme and requires restart. The technique of moment matching allows to find a set of parameters that is able to give the same set of moments (and thus theoretically the same distribution/statistics) of a given random variable. This allows to select appropriate initial conditions for the parameters that avoid inconsistencies with the data. Note that for simple processes (for example the 1-type BGW) moment matching allows in principle to fully determine the parameters of the process starting from statistical observations such as mean, standard deviations and higher order moments. As detailed in the following, the situation for multi-type processes is far more complex. The approach below was followed for the inference presented in Figure 3D of the main text.

#### Moments of a multi-type Galton-Watson model

Let us start by considering a 3-types BGW model as described in the manuscript, characterized by the following PGFs:

(Equation 47) $$f_{1}\left(s_{1},s_{2},s_{3}\right)=\theta_{10}+\theta_{11}s_{1}+\theta_{12}s_{2}+\theta_{13}s_{3}+\theta_{14}s_{1}^{2}+\theta_{15}s_{1}s_{2}+\theta_{16}s_{1}s_{3}+\theta_{17}s_{2}^{2}+\theta_{18}s_{3}^{2}+\theta_{19}s_{2}s_{3}$$

$$f_{2}\left(s_{1},s_{2},s_{3}\right)=\theta_{20}+\theta_{21}s_{2}+\theta_{22}s_{1}+\theta_{23}s_{3}+\theta_{24}s_{2}^{2}+\theta_{25}s_{2}s_{1}$$

(Equation 48) $$+\theta_{26}s_{2}s_{3}+\theta_{27}s_{1}^{2}+\theta_{28}s_{3}^{2}+\theta_{29}s_{1}s_{3}$$

$$f_{3}\left(s_{1},s_{2},s_{3}\right)=\theta_{30}+\theta_{31}s_{3}+\theta_{32}s_{1}+\theta_{33}s_{2}+\theta_{34}s_{3}^{2}+\theta_{35}s_{3}s_{1}$$

(Equation 49) $$+\theta_{36}s_{3}s_{1}+\theta_{37}s_{1}^{2}+\theta_{38}s_{2}^{2}+\theta_{39}s_{1}s_{2}$$

If we consider each type to start with a generic initial population *Y*(0)=(*Y*_1_,*Y*_2_,*Y*_3_), then the PGFs for the sub-populations can be written as

(Equation 50) $$f_{Y_{1}}\left(s_{1},s_{2},s_{3}\right)=f_{1}{\left(s_{1},s_{2},s_{3}\right)}^{Y_{1}}$$

(Equation 51) $$f_{Y_{2}}\left(s_{1},s_{2},s_{3}\right)=f_{2}{\left(s_{1},s_{2},s_{3}\right)}^{Y_{2}}$$

(Equation 52) $$f_{Y_{3}}\left(s_{1},s_{2},s_{3}\right)=f_{3}{\left(s_{1},s_{2},s_{3}\right)}^{Y_{3}}$$

As detailed in the text, within BGW the random variable that describes the population dynamics is the random variable that contains the offspring of each type *m* at the first generation, indicated with ${\vec{X}}_{m}\left(1\right)={\vec{X}}_{m}=\left(X_{m,1},\ldots ,X_{m,M}\right)$. The components *X*_*m*,*i*_ represent the number of descendants of type *i* generated by type *m* in one generation, and where each index runs over {1, …,3}. The relationship between the number of cells of the *i*-th type and the offspring random variable {*X*_*i*_} is given by the equation:

(Equation 53) $$Y_{i}\left(1\right)=\sum_{m=1}^{3}\sum_{k=1}^{Y_{m}\left(0\right)}X_{mi}^{\left(k\right)}$$

where the first sum is over the cell types, the second one is over the cells of a given type.

If one is interested in the moments of the process, it is convenient to work with the moment generating functions (rather than the PGFs), easily found from the PGFs through the substitution $s_{i}\to e^{s_{i}}$:

$$M_{1}\left(\vec{s}\right)=\theta_{10}+\theta_{11}e^{s_{1}}+\theta_{12}e^{s_{2}}+\theta_{13}e^{s_{3}}+\theta_{14}e^{2s_{1}}+\theta_{15}e^{s_{1}+s_{2}}$$

(Equation 54) $$+\theta_{16}e^{s_{1}+s_{3}}+\theta_{17}e^{2s_{2}}+\theta_{18}e^{2s_{3}}+\theta_{19}e^{s_{2}+s_{3}}$$

$$M_{2}\left(\vec{s}\right)=\theta_{20}+\theta_{21}e^{s_{2}}+\theta_{22}e^{s_{1}}+\theta_{23}e^{s_{3}}+\theta_{24}e^{2s_{2}}+\theta_{25}e^{s_{2}+s_{1}}$$

(Equation 55) $$+\theta_{26}e^{s_{2}+s_{3}}+\theta_{27}e^{2s_{1}}+\theta_{28}e^{2s_{3}}+\theta_{29}e^{s_{1}+s_{3}}$$

$$M_{3}\left(\vec{s}\right)=\theta_{30}+\theta_{31}e^{s_{3}}+\theta_{32}e^{s_{1}}+\theta_{33}e^{s_{2}}+\theta_{34}e^{2s_{3}}+\theta_{35}e^{s_{3}+s_{1}}$$

(Equation 56) $$+\theta_{36}e^{s_{3}+s_{1}}+\theta_{37}e^{2s_{1}}+\theta_{38}e^{2s_{2}}+\theta_{39}e^{s_{1}+s_{2}}$$

The **first moment** can be found through the relation

(Equation 57) $$E\left[Y_{i}\right]=\sum_{m}\sum_{k}^{Y_{m}\left(0\right)}E\left[X_{mi}^{\left(k\right)}\right]=\sum_{m}Y_{m}\left(0\right)E\left[X_{mi}\right]=\sum_{m}Y_{m}\left(0\right)\frac{\partial M_{m}}{\partial s_{i}}{|}_{\vec{s}=\vec{0}}$$

We are also interested in the **second moment**:

(Equation 58) $$E\left[Y_{i}{\left(1\right)}^{2}\right]=E\left[Y_{i}\left(1\right)Y_{i}\left(1\right)\right]=E\left[\left(\sum_{m}^{3}\sum_{k}^{Y_{m}\left(0\right)}X_{mi}^{\left(k\right)}\right)\left(\sum_{m^{\prime }}^{3}\sum_{k^{\prime }}^{Y_{m^{\prime }}\left(0\right)}X_{m^{\prime }i}^{\left(k^{\prime }\right)}\right)\right]$$

(Equation 59) $$=E\left[\left(\sum_{m}^{3}\sum_{m^{\prime }}^{3}\sum_{k}^{Y_{m}\left(0\right)}\sum_{k^{\prime }}^{Y_{m^{\prime }}\left(0\right)}X_{mi}^{\left(k\right)}X_{m^{\prime }i}^{\left(k^{\prime }\right)}\right)\right]$$

(Equation 60) $$=\sum_{m}^{3}\sum_{m^{\prime }}^{3}\sum_{k}^{Y_{m}\left(0\right)}\sum_{k^{\prime }}^{Y_{m^{\prime }}\left(0\right)}E\left[X_{mi}^{\left(k\right)}X_{m^{\prime }i}^{\left(k^{\prime }\right)}\right]$$

There are 3 contributions in the above equation.

1. *m* = *m*′, *k* = *k*′: same type, same cell

(Equation 61) $$E\left[X_{mi}^{\left(k\right)}X_{mi}^{\left(k\right)}\right]=E\left[X_{mi}^{2}\right]=\frac{\partial^{2}M_{m}}{\partial s_{i}^{2}}{|}_{\vec{s}=\vec{0}}$$

2. *m* = *m*′, *k*≠*k*′: same type, different cells

(Equation 62) $$E\left[X_{mi}^{\left(k\right)}X_{mi}^{\left(k^{\prime }\right)}\right]=E\left[X_{mi}^{\left(k\right)}\right]E\left[X_{mi}^{\left(k^{\prime }\right)}\right]={\left(\frac{\partial M_{m}}{\partial s_{i}}{|}_{\vec{s}=\vec{0}}\right)}^{2}$$

3. *m*≠*m*′, *k*≠*k*′: different types, different cells

(Equation 63) $$E\left[X_{mi}^{\left(k\right)}X_{m^{\prime }i}^{\left(k^{\prime }\right)}\right]=E\left[X_{mi}^{\left(k\right)}\right]E\left[X_{m^{\prime }i}^{\left(k^{\prime }\right)}\right]=\frac{\partial M_{m}}{\partial s_{i}}{|}_{\vec{s}=\vec{0}}\frac{\partial M_{m^{\prime }}}{\partial s_{i}}{|}_{\vec{s}=\vec{0}}$$

Gathering all these terms together, we obtain:

$$E\left[Y_{i}{\left(1\right)}^{2}\right]=\sum_{m}Y_{m}\left(0\right)\frac{\partial^{2}M_{m}}{\partial s_{i}^{2}}{|}_{\vec{s}=\vec{0}}$$

$$+\sum_{m}Y_{m}\left(0\right)\left(Y_{m}\left(0\right)-1\right){\left(\frac{\partial M_{m}}{\partial s_{i}}{|}_{\vec{s}=\vec{0}}\right)}^{2}$$

$$+\sum_{m}\sum_{m^{\prime }}Y_{m}\left(0\right)Y_{m^{\prime }}\left(0\right)\frac{\partial M_{m}}{\partial s_{i}}{|}_{\vec{s}=\vec{0}}\frac{\partial M_{m^{\prime }}}{\partial s_{i}}{|}_{\vec{s}=\vec{0}}$$

In the same way we can obtain the **cross moments**:

$$E\left[Y_{i}\left(1\right)Y_{j}\left(1\right)\right]=E\left[\left(\sum_{m}^{3}\sum_{k}^{Y_{m}\left(0\right)}X_{mi}^{\left(k\right)}\right)\left(\sum_{m^{\prime }}^{3}\sum_{k^{\prime }}^{Y_{m^{\prime }}\left(0\right)}X_{m^{\prime }j}^{\left(k^{\prime }\right)}\right)\right]$$

$$=E\left[\sum_{m}^{3}\sum_{m^{\prime }}^{3}\sum_{k}^{Y_{m}\left(0\right)}\sum_{k^{\prime }}^{Y_{m^{\prime }}\left(0\right)}X_{mi}^{\left(k\right)}X_{m^{\prime }j}^{\left(k^{\prime }\right)}\right]$$

$$=\sum_{m}^{3}\sum_{m^{\prime }}^{3}\sum_{k}^{Y_{m}\left(0\right)}\sum_{k^{\prime }}^{Y_{m^{\prime }}\left(0\right)}E\left[X_{mi}^{\left(k\right)}X_{m^{\prime }j}^{\left(k^{\prime }\right)}\right]$$

There are again three contributions in the above equation.

1. *m* = *m*′, *k* = *k*′: same type, same cell

(Equation 64) $$E\left[X_{mi}^{\left(k\right)}X_{mj}^{\left(k\right)}\right]=\frac{\partial^{2}M_{m}}{\partial s_{i}\partial s_{j}}{|}_{\vec{s}=\vec{0}}$$

2. *m* = *m*′, *k*≠*k*′: same type, different cells

(Equation 65) $$E\left[X_{mi}^{\left(k\right)}X_{mj}^{\left(k^{\prime }\right)}\right]=E\left[X_{mi}^{\left(k\right)}\right]E\left[X_{mj}^{\left(k^{\prime }\right)}\right]=\frac{\partial M_{m}}{\partial s_{i}}{|}_{\vec{s}=\vec{0}}\frac{\partial M_{m}}{\partial s_{j}}{|}_{\vec{s}=\vec{0}}$$

3. *m*≠*m*′, *k*≠*k*′: different types, different cells

(Equation 66) $$E\left[X_{mi}^{\left(k\right)}X_{m^{\prime }j}^{\left(k^{\prime }\right)}\right]=E\left[X_{mi}^{\left(k\right)}\right]E\left[X_{m^{\prime }j}^{\left(k^{\prime }\right)}\right]=\frac{\partial M_{m}}{\partial s_{i}}{|}_{\vec{s}=\vec{0}}\frac{\partial M_{m^{\prime }}}{\partial s_{j}}{|}_{\vec{s}=\vec{0}}$$

Gathering all these terms together, we obtain:

$$E\left[Y_{i}\left(1\right)Y_{j}\left(1\right)\right]=\sum_{m}Y_{m}\left(0\right)\frac{\partial^{2}M_{m}}{\partial s_{i}\partial s_{j}}{|}_{\vec{s}=\vec{0}}$$

$$+\sum_{m}Y_{m}\left(0\right)\left(Y_{m}\left(0\right)-1\right)\frac{\partial M_{m}}{\partial s_{i}}{|}_{\vec{s}=\vec{0}}\frac{\partial M_{m}}{\partial s_{j}}{|}_{\vec{s}=\vec{0}}$$

$$+\sum_{m}\sum_{m^{\prime }}Y_{m}\left(0\right)Y_{m^{\prime }}\left(0\right)\frac{\partial M_{m}}{\partial s_{i}}{|}_{\vec{s}=\vec{0}}\frac{\partial M_{m^{\prime }}}{\partial s_{j}}{|}_{\vec{s}=\vec{0}}$$

At this point, we only need to evaluate the terms containing the derivatives of the mgfs:

(Equation 67) $$E\left[X_{mi}X_{mj}\right]=\frac{\partial M_{X_{m}}}{\partial s_{i}\partial s_{j}}{|}_{\vec{s}=\vec{0}}$$

When *i*≠*j* these are:

$$E\left[X_{11}X_{12}\right]=\theta_{15}\;E\left[X_{11}X_{13}\right]=\theta_{16}\;E\left[X_{12}X_{13}\right]=\theta_{19}$$

$$E\left[X_{21}X_{22}\right]=\theta_{25}\;E\left[X_{21}X_{23}\right]=\theta_{26}\;E\left[X_{22}X_{23}\right]=\theta_{29}$$

$$E\left[X_{33}X_{31}\right]=\theta_{35}\;E\left[X_{33}X_{32}\right]=\theta_{36}\;E\left[X_{31}X_{32}\right]=\theta_{39}$$

whereas when *i* = *j* these values are:

$$E\left[X_{11}^{2}\right]=2\theta_{14}+E\left[X_{11}\right]\;E\left[X_{12}^{2}\right]=2\theta_{17}+E\left[X_{12}\right]\;E\left[X_{13}^{2}\right]=2\theta_{18}+E\left[X_{13}\right]$$

$$E\left[X_{21}^{2}\right]=2\theta_{27}+E\left[X_{21}\right]\;E\left[X_{22}^{2}\right]=2\theta_{24}+E\left[X_{22}\right]\;E\left[X_{23}^{2}\right]=2\theta_{28}+E\left[X_{23}\right]$$

$$E\left[X_{31}^{2}\right]=2\theta_{37}+E\left[X_{31}\right]\;E\left[X_{32}^{2}\right]=2\theta_{38}+E\left[X_{32}\right]\;E\left[X_{33}^{2}\right]=2\theta_{34}+E\left[X_{33}\right]$$

#### Moment matching

Now we want to perform moment matching, i.e., determining the parameters in such a way that the theoretically obtained moments (as a function of the parameters) correspond to those obtained empirically from data. The obtained solution will act as a starting point to infer the underlying parameters with MCEM. Note that, for simplicity, we wrote this paragraph thinking to the data shown in Figure S13. Therefore, we suppose to have cell counts *Y* at two consecutive generations 0 and 1, so that, in what follows, *Y*(1) can be thought as the number of cells at one generation, knowing the number of cells at the previous generation.

Let us denote with ⟨⟩the empirical average. We therefore have access to all of the following quantities:

$$\left\{\left\langle Y_{m}\left(1\right)\right\rangle ,\left\langle Y_{m}^{2}\left(1\right)\right\rangle ,\left\langle Y_{m}\left(1\right)Y_{m^{\prime }}\left(1\right)\right\rangle ,\ldots \right\}$$

We then have a (non-linear) system of equations with the parameters as unknown:

(Equation 68) $$\left\{\begin{array}{l} E\left[Y_{m}\left(1\right)\right]=\left\langle Y_{m}\left(1\right)\right\rangle \\ E\left[Y_{m}^{2}\left(1\right)\right]=\left\langle Y_{m}^{2}\left(1\right)\right\rangle \\ E\left[Y_{m}\left(1\right)Y_{m^{'}}\left(1\right)\right]=\left\langle Y_{m}\left(1\right)Y_{m^{'}}\left(1\right)\right\rangle \\ \text{Var}\left(Y_{m}\left(1\right)\right)=\left\langle Y_{m}^{2}\left(1\right)\right\rangle -{\left\langle Y_{m}\left(1\right)\right\rangle }^{2}\\ E\left[Y_{m}^{2}\left(1\right)Y_{m^{'}}^{2}\left(1\right)\right]=\left\langle Y_{m}\left(1\right)Y_{m^{'}}\left(1\right)\right\rangle \\ \text{Cov}\left(Y_{m}\left(1\right)Y_{m^{'}}\left(1\right)\right)=\left\langle Y_{m}\left(1\right)Y_{m^{'}}\left(1\right)\right\rangle -\left\langle Y_{m}\left(1\right)Y_{m^{'}}\left(1\right)\right\rangle \\ \vdots \end{array}\right)$$

These are 18 relations in total, to which a normalization condition for each phenotype can be added: $\sum_{t}\theta_{mt}=1$. Therefore, Equation 68 represents a non-linear undetermined system of Equation 21 conditions for 30 unknowns). In order to increase the number of equations, we considered datasets as being organized into different unique initial conditions:

$$\left\{\left(Y_{1,r}\left(0\right),Y_{2,r}\left(0\right),Y_{3,r}\left(0\right)\right)\right\}$$

where each *r* is an index for a different initial configuration. At this point, we can write 18 equations for each index *r*, and find (numerically) a solution Equation 68.

In order to find a set of parameters satisfying Equation 68, we resort to the numerical minimization of the sum of the squares of the terms:

(Equation 69) $$\begin{array}{l} \min_{\Theta }\{\sum_{r}[\;\sum_{m}{\left(\sum_{t}\theta_{mt}-1\right)}^{2}+\sum_{m}{\left(E\left[Y_{m,r}\left(1\right)\right]-\left\langle Y_{m,r}\left(1\right)\right\rangle \right)}^{2}+\sum_{m}{\left(E\left[Y_{m,r}^{2}\left(1\right)\right]-\left\langle Y_{m,r}^{2}\left(1\right)\right\rangle \right)}^{2}+{\sum_{m}\sum_{m^{\prime }\neq m}\left(E\left[Y_{m,r}\left(1\right)Y_{m^{\prime },r}\left(1\right)\right]-\left\langle Y_{m,r}\left(1\right)Y_{m^{\prime },r}\left(1\right)\right\rangle \right)}^{2}+\sum_{m}{\left(\text{Var}\left(Y_{m,r}\left(1\right)\right)-\left\langle Y_{m,r}^{2}\left(1\right)\right\rangle +{\left\langle Y_{m,r}\left(1\right)\right\rangle }^{2}\right)}^{2}+{\sum_{m}\sum_{m^{\prime }\neq m}\left(\text{Cov}\left(Y_{m,r}\left(1\right)Y_{m^{\prime },r}\left(1\right)\right)-\left\langle Y_{m,r}\left(1\right)Y_{m^{\prime },r}\left(1\right)\right\rangle +\left\langle Y_{m,r}\left(1\right)\right\rangle \left\langle Y_{m^{\prime },r}\left(1\right)\right\rangle \right)}^{2}]\} \end{array}$$

where the minimization is performed over the entire set of parameters. Note however that once a minimum is found, it is still necessary to verify that it is a solution to the system.

In practice, we considered three different initial conditions:

$$Y_{1,1}\left(0\right)=5,Y_{2,1}\left(0\right)=32,Y_{3,1}\left(0\right)=3$$

$$Y_{1,2}\left(0\right)=6,Y_{2,2}\left(0\right)=30,Y_{3,2}\left(0\right)=4$$

$$Y_{1,3}\left(0\right)=3,Y_{2,3}\left(0\right)=27,Y_{3,3}\left(0\right)=2$$

Each of these initial conditions was used to generate via numerical simulations of BGW process a total of 1500 realizations with the given parameters. At this point, 1500 × 3 data series spanning one generation (one for each initial condition) were used to write 18 × 3 equations to minimize Equation 69. For each of these we obtained a value for the 30 parameters, as shown in Figure S17.

#### Numerical simulations of BGW processes

Simulations of the BGW process are generated using a Python script. Given an initial number of cells with a known phenotype composition, we simulate a given number of generations. At each generation, for each cell of phenotype *m*, we randomly select a possible transition according to the offspring distribution associated with that phenotype. This is simply done by throwing a random number and deciding which transition within ${\vec{\theta }}_{m}$ the cell will undergo. We then update the number of cells for each phenotype, taking into account that different transitions lead to different changes in the sub-populations. In our setup, the only possible changes for a sub-population due to a cell of phenotype *j* are: Δ*n*_*j*_∈{-1,0,1,2}. It is important to note that the order used to list the transitions is arbitrary, and alternative orderings are equally valid. Finally, when feedback mechanisms are incorporated, the offspring distribution is updated after each change in the sub-populations, according to a predefined mathematical rule. This allows the model to capture scenarios in which the parameters depend on the total number of cells in the population or on the number of cells within specific sub-populations. Python scripts to simulate BGW processes can be found on our dedicated github: https://github.com/AndreaPiras21/Inference-Cells/ accessible at the following DOI: https://doi.org/10.5281/zenodo.21276633.

#### Lineage inference in BGW models of 1 cell type populations

Here we describe how to apply the different developed methodologies to a number of biologically relevant situations, represented by *in silico* data generated by numerical simulations of BGW branching processes.

This example can be linked to several biological contexts (Figure S10A), such as a single cell developing into a cell colony, a spheroid, an tumoroid or a developing embryo in the zygote to morula stage, prior to any cell fate specification. The possible transitions for each cell are cell death, standby and self-renewal, as illustrated in Figure S10B. In order to present the different scenarios where our inference approach can be applied we numerically simulated a BGW branching process and generated different datasets corresponding to different experimental setups all with the same parameter values, as shown in Figure S10B. A plot of the typical set of realizations used is shown in Figure S10C. Note that, at variance with continuous models, the probability of extinction is strictly positive in any nontrivial BGW branching process, and therefore it is possible that a single cell does not give rise to a progeny even if on average the population is growing.

• *Tracking all cells* First we consider the case where cell tracking is available, i.e., we assume full knowledge of the history of each single cell in terms of transitions on all generations. To this aim we consider ten distinct data sequences generated by numerically simulating a BGW process, starting from a single cell and lasting 8 generations.
  This situation can be easily tackled analytically. Across the whole dataset, 107 cells died, there were 180 standbys and 415 cell divisions. The likelihood of such observation can be obtained by factorizing the single tree likelihoods or just considering the total number of events: $L\left(\vec{z}|\vec{\theta }\right)=\theta_{0}^{107}\theta_{1}^{180}\theta_{2}^{415}$. The one final step is to obtain the posterior from such a likelihood which can be easily done by recalling normalization conditions for Dirichlet distributions. A direct application of Bayes’ rule with a flat prior and such a likelihood then leads to the posterior shown in Figure S10D. This result is in agreement with the true values used to generate the observations and the width of the distributions expresses the statistical uncertainty originating from the finite number of data points used to infer the parameters.
  Thanks to the mathematical properties of BGW processes, cell tracking is phrased into knowing all possible transitions that occurred within a given number of generations rather than really knowing all the different trees. Therefore, in an experimental situation, the information required is the knowledge of how many cells died, how many cell division were observed, while the standbys are automatically calculated by counting the total number of cells, as shown below. This represents an experimentally relevant point as one can use temporally well resolved timelapse experiments and recognize transitions (e.g., mitoses, cell death) without the need to explicitly track cells one by one.
• *Consecutive measurements with no tracking.* Our methodology can also be used when single cell tracking is not available and, for example, we only have access to the total number of cells at each generation, rather than the pedigree of each cell. We therefore consider the same dataset as before but assuming no direct connection between data at different generations. This case was analyzed using our analytical method and the results are shown in Figure S10E. Here, we present an iterative approach, where data are incorporated one by one into the likelihood, providing insights about the convergence of the estimations. Our results show that, even without recurring to cell tracking, parameters can be inferred and the distributions are concentrating around the true values.
  Analogous conclusions were reached by employing the EGS to a larger dataset, where now the marginals are computed numerically (as shown in Figure S10F). Note that the latter approach is computationally more efficient than the analytical one which is why more sequences, or datasets with more cells can be analyzed.
• *Connected endpoint measurements.* We now turn our attention to the case when only the population size at the initial and final generations is measured. This corresponds to an experiment where single cells are seeded and grown for an appropriate time, assuming no mixing is occurring, and where cells are then fixed and counted at the end of the experiment. In this context, ’no mixing’ means that cells coming from different ancestors are never exchanged, i.e., they are either very far away or do not move appreciably. This situation can be theoretically addressed both by the analytical and the EGS methods, but becomes rapidly intractable when the number of elapsed generations increases. Such data series can however be analyzed using the MCEM algorithm. This algorithm requires a relatively larger amount of data to produce reliable estimates in contrast with the two previous approaches. To show the power of this approach, we considered a much larger dataset, starting from a single cell, and including only the counts at the 8-th generation. This dataset was then processed with the MCEM algorithm and the convergence to the true values was reached after only a few steps, as shown in Figure S10G.
• *Unconnected endpoint measurements.* Finally, we consider a situation in which data from tumoroids or cell colonies are independent at each generation, i.e., each observation is obtained by seeding a single cell and then fixing at a given generation and the experiment is repeated for different final generations, as shown in fig. SH. Data generated in this way does not allow to the use conditional probability on consecutive generations *p*(*y*(*n*+1)|*y*(*n*)) since for a given data-point *y*(*n*+1) the corresponding *y*(*n*) is unknown.
  One possible approach is to marginalize over all possible *y*(*n*), giving $p\left(y\left(n+1\right)\right)=\sum_{y}p\left(y\left(n+1\right)|y\left(n\right)\right)$. Inference with this approach however leads to a larger uncertainty with respect to when the previous data point is known, as it indeed includes fewer transitions and hence less information. Furthermore, this approach becomes computationally intensive for large population sizes or large number of types as the number of possible previous conditions to sum over becomes large. To overcome these issues we approximate the marginalization by summing just over the populations sizes *y* observed at the previous generation $p\left(y\left(n+1\right)\right)=\sum_{y}p\left(y\left(n+1\right)|y\left(n\right)\right)≃\sum_{y_{obs}}p\left(y\left(n+1\right)|y\left(n\right)\right)$.
  In order to perform inference we then apply MCEM by using 100 observations for each generation, as shown in Figure S10I. In this way we can recover inferred parameters for each generation of observations and such estimates can be combined to give a more precise global estimate over the whole dataset.
  It is worth remarking that this approach does not require cell tracking nor consecutive observations. As such, it is ideally suited to the case when measurements are based on fixed snapshots obtained at different times, a situation which is frequently encountered experimentally. While this is admittedly a less rigorous methodology than the other ones presented in this section, it can however prove useful in experimental setups, provided that data for each generation are sufficiently representative of all possible realizations.

#### Clonal expansion with feedback: The case of contact inhibition

Here we consider the case where a cell colony, spheroid or tumoroid, made of cells of a single type is subject to a feedback on its growth that depends on the population size. A common example is when proliferation rates depend on the local density on the population size. For instance, the release of promoting or inhibiting factors might increase (or decrease) the self-renewal probability. As another example, nutrient consumption or release of toxins, which increase with population size, might then in turn increase the probability of death.

We consider contact inhibition of growth as a reference biological situation.^82^ In this situation, once confluence is reached, each cell will undergo several rounds of cell division while slowing down and will be reaching a regime where rare cell divisions are in equilibrium with rare cell deaths.^60^^,^^66^ Cells can therefore grow with a self-renewal probability which decreases as the number of cells in the progeny increases, by increasing the probability of inactivation. In the following, we assume a simple linear functional form to define a BGW model capable of generating data with constrained dynamics, as sketched in Figure S11A. Mathematically, the functions governing the model parameters are defined as follows:

(Equation 70) $$\left\{ \begin{array}{l} \theta_{0}\left(Y\right)=\theta_{0}\\ \theta_{1}\left(Y\right)=1-\theta_{0}-\theta_{2}\left(1-\frac{Y}{K}\right)\\ \theta_{2}\left(Y\right)=\theta_{2}\left(1-\frac{Y}{K}\right) \end{array}\right.$$

where *K* is a maximum clone size, constraining local density in the tissue, and $\vec{\theta }$ are analogous to the previous case. Inspired by experimental evidence on contact inhibition of growth in epithelial cell colonies,^60^ we set *K* = 30. This choice is motivated by the fact that cells can reduce their projected area of around a factor *K* from when they fill the culture dish to when they arrest their growth. We performed numerical simulations with initial parameters $\vec{\theta }=\left(0.2,0,0.8\right)$, as shown in Figure S11B. Here Markovianity is assured by the fact that the probability of obtaining a population size *Y*(*n*+1) starting from *Y*(*n*) depends only on *Y*(*n*) as in Equation 70. Note that the precise choice of functional dependence is only needed to generate the synthetic data, but its knowledge is not required to perform the inference. Rather, it is the inference itself which reconstructs the functional form of the feedback.

All data generated from a given *Y*, regardless of the specific generation in which it occurs, can be grouped together by population size and used to infer the parameter values for that population size $\vec{\theta }\left(Y\right)$. The results of the inference performed with the EGS are shown in Figure S11C, where the mean and standard deviation of the marginals are displayed as a function of the clone size *Y*.

The comparison between the true and inferred parameter values clearly shows that our approach can be used to estimate the functional form of the feedback starting from data on the population size. Note that in all approaches presented here, inference is performed on data without tracking, assuming only the knowledge of the population size at all generations. Larger data-series can be handled by means of the MCEM approach which allows computationally more efficient inference. Results obtained by this method are shown in Figure S11D and highlight once more the capability of our approach to capture the true values with no prior knowledge.

#### Treating a one type cell population

We begin by addressing a simple 1-type model, illustrated in Figure S12A. This case can be tackled by means of different approaches, presented throughout the paper. First we imagine we can count all occurred transitions, and we therefore write the analytical expression for the posterior. A variation in the observation of the different transitions directly maps into a change of parameters which is captured by analytical inference (as shown in Figure S12B). If during an experiment we witness 620 cells dead, only 39 cells dividing and 247 cells standing by, we can write the posterior as $p\left(\Theta |Y\right)=\theta_{0}^{620}\theta_{1}^{247}\theta_{2}^{39}/N$, where N is the normalization factor. Alternatively, if detailed information on the transitions is not available, one can either apply the analytical or the EGS method to infer transitions in this case. Results of the inference performed with the MCEM method are shown in Figure S12D, which, as shown previously, can reach larger number of cells and observations.

In order to rationalize the effect of the therapy one can exploit all the formalism of the BGW branching processes, calculating extinction probability at a given generation, the mean growth and the ratio between the different transition probabilities. For example, the model shown in Figure S12A is predicted to grow with an average number of cells at the *n*-th generation given by $m=Y_{0}{\left(\theta_{1}+2\theta_{2}\right)}^{n}=Y_{0}{\left(1+\theta_{2}-\theta_{0}\right)}^{n}$, where *Y*_0_ is the number of cells at the beginning of the treatment. Therefore we can interpret the changes in parameters in the following way: a cytostatic drug will mostly impact on *θ*_1_, while a cytotoxic drug would instead impact the parameter *θ*_0_ by necessarily reducing inactivity and proliferation probabilities and thus affecting the growth rate. For example, a compound leaving the difference *θ*_2_-*θ*_0_ unchanged would not change the overall growth rate but would result in a different standard deviation and would therefore be distinguishable from the untreated case, in contrast with a continuous process. It is worth noting that in presence of parameters inferred in both untreated and treated conditions, it is also possible to evaluate the effect of a drug by measuring the changes in the probability of extinction which can be calculated analytically thanks to the BGW formalism.

#### 3-types plastic cell populations

As an even more complete example showing the power of our approach we present here the case of a stem/progenitor cell compartment (*m* = 1) which can generate transit-amplifying cells (TA, *m* = 2). These in turn can also expand or transition into differentiated cells (*m* = 3), as shown in Figures S13A–S13C. The biological reference situation is similar to that of many tissues where however we allow differentiated cells to plastically dedifferentiate.^54^ Similar examples are encountered upon injury or ablation in healthy tissues^53^^,^^55^^,^^56^^,^^83^^,^^84^ or in cancer.^7^^,^^8^^,^^9^^,^^10^

In order to tackle this scenario, we generated 10000 observations collected after 3 generations starting from one stem cell. On such data we performed inference using MCEM with no prior knowledge and inferred all the 30 parameters of the full model. The comparison between the true values and the estimates obtained exploiting our MCEM algorithm is shown in Figure S13D. Once again our approach proves itself successful even in a relatively complex case of a 3 populations hierarchy with no prior knowledge on the transitions.

From an inference perspective, identifying biologically relevant parameters in the full model shown in Figure 1A for 3 distinct cell types, translates into finding 30 parameters in total and therefore requires a substantially larger amount of data with respect to the previous cases. This situation can be attenuated if one introduces a prior knowledge on transitions, for example lack of cell division for differentiated cells, or specifying the type of division (whether symmetric or asymmetric) for stem cells or TA cells.

Implementation of feedback on the total population for multi-type models.

In this section we consider the effect of feedback on two different phenotypes. A possible biologically relevant scenario is the following. Suppose cells are able to degrade the medium or release certain inhibitory factors. Assume then that these processes inhibits the proliferation and the differentiation of stem cells while promoting quiescence and suppressing reversal to proliferative phenotype for differentiated cells, as shown in Figure S15A. We can model this situation with the following two-type BGW probabilities

(Equation 71) $$\left\{ \begin{array}{l} \theta_{10}\left(Y\right)=\theta_{10}\\ \theta_{11}\left(Y\right)=0\\ \theta_{12}\left(Y\right)=1-\theta_{10}-\theta_{13}\left(Y\right)\\ \theta_{13}\left(Y\right)=\theta_{13}\left(1-\frac{Y}{K}\right) \end{array}\right.\;\left\{ \begin{array}{l} \theta_{20}\left(Y\right)=\theta_{20}\\ \theta_{21}\left(Y\right)=1-\theta_{21}-\theta_{22}\left(Y\right)\\ \theta_{22}\left(Y\right)=\theta_{22}\left(1-\frac{Y}{K}\right) \end{array}\right.$$

This model leads to a constrained growth in both subpopulations as shown in Figure S15B and promotes a change of abundance of cell of the two types, displaying stem cell dominated growth when *Y* is small and viceversa when *Y* is large. In order to perform inference with this model we generated 1000 simulations starting with one stem cell. Results of the inference are shown in Figure S15D together with a comparison with the true parameters.

#### Inferring from non-clonal or asynchronous experimental data

Here we aim at testing our method against data where generations are not perfectly synchronous, for which the BGW branching process is only an approximation. Deviations from synchrony may take place because the initial condition is made of several non synchronized cells, or because division times are highly variable. In the first case, if the number of cells is very large, one might prefer to adopt a different model than BGW from the outset.

Clonal dynamics, i.e., the growth of small cell groups or single cells developing into a clone, is well suited to our approach as doubling times in cell populations are empirically found to be distributed according to Erlang distribution with relatively large shape parameters *k*.^60^^,^^61^^,^^62^^,^^63^ Similar results have been shown for embryos,^64^^,^^65^ while these distributions are significantly more dispersed in bacterial populations.^85^ For narrow doubling time distributions, the typical time for which the offspring remains significantly correlated is several generations or doubling times.

In order to show the performance of our method in this setting, we generated several numerical simulations with different doubling time statistics, i.e., generated with Erlang distribution with shape parameter *k* = 10,50,100, and rate parameters $\tilde{\lambda }k$ so that the mean doubling time mean is $\mu =\tilde{\lambda }=1$, as shown in Figure S16. The relative dispersion for such distributions would be:

(Equation 72) $$\frac{\sqrt{\sigma^{2}}}{\mu }=\frac{\sqrt{\frac{k}{{\tilde{\lambda }}^{2}k^{2}}}}{\tilde{\lambda }k}=\frac{1}{\sqrt{k}}$$

where *k* directly controls correlation between siblings (with *k* = 1 being the Poissonian case and *k*→*∞* representing BGW). Numerical simulations of these birth-death processes were obtained by implementing Gillespie’s method.^86^ Cell death was simulated with a Poissonian process with rate *δ* (in the absence of more refined hypotheses), while cell proliferation would occur after *k* Poissonian steps of rate *λ*, yielding an Erlang distribution for doubling time with shape parameter *k* and rate parameter $k\tilde{\lambda }$.

To demonstrate the effectiveness of our approach, we conducted tests using two different parameter sets, keeping the cell’s duplication time equal to 1. In the first series, we assumed no cell death (Figure S16A), while in the second we enforced an approximate division probability per generation of 0.9 and a death probability per generation of 0.1 (Figure S16B).

To carry out inference on the growth curves, we began by sampling the data under the imposed doubling time *μ* = 1. We then aligned the trajectories to avoid ill-posed configurations, such as multiple consecutive divisions within a single generation time. To achieve this, we used numerically generated sequences of the form [*t*,*Y*(*t*)] and applied a mean smoothing filter with a window size of 0.35*μm*. This allowed us to identify contiguous regions with non-positive derivatives. Among these, we decided to discard those shorter than 35% of the doubling time.

We then searched for an appropriate time offset of the data such that sampling times *nT*_*div*_ with (*n* = 1,2,3) would fall within the non positive derivative regions. Finally, we inferred transition probabilities using this sampled dataset.

For shape parameters and death rates similar to those encountered in experimental data, inference shows good results in both considered cases, as shown in Figures S16A and S16B. Our results indicate that deviations between true and inferred values lie within a range of 10% even considering asynchronous data closer to Poisson processes. Note that uncertainty is present also when data series are extracted from BGW processes as the amount of data is finite.

As expected, when lowering the shape parameters toward Poissonian dynamics inference is less and less reliable. One issue that is encountered is that different generations can overlap when cells become asynchronous, resulting in configurations that are incompatible with the model (for example observing more than 2^*n*^ cells at generation *n*). These cases can be handled in different ways, but would technically correspond to null likelihood and should therefore be discarded. In general this issue is present whenever within one generation cells perform two consecutive transitions (for example two divisions or one division and one death). Cell death can easily be counted as happening at the next generation or, different definition of generation time can be chosen in order to solve this issue, for example half the division time. Another possibility is to introduce dedicated cell types which die with probability one. This new type, while fictitious and not directly measurable biologically, can introduce the kind of correlated behavior observed in experimental data, where generally the probability of cell death is very low but often happening in daughter cells.

#### Inference of transition probabilities in hematopoiesis

##### Aggregating transitions

In order to aggregate transitions into unique probabilities as in ref.^68^ (Figure 6D) we calculated the mean number of offspring by leveraging BGW formalism. For example, the probability of LES cells of generating other LES cells includes two different contributions, one coming from self-renewal and the other coming from inactivation. For brevity here we indicate LES: 1, G2H: 2, G1/2H: 3, P1H: 4 (please note that, however, indexing of transition probabilities refer to Figure 6A in the main text). Therefore one LES cell will generate on average *θ*_11_+2*θ*_12_ LES cells (which are the corresponding probabilities of inactivation and self-renewal). One LES cell will also generate on average *θ*_13_ P1H and G2H cells. Therefore, by just normalizing these average number of cells over all the offspring one obtains the proportions of cells of each given type generated by the type LES, i.e., *p*_11_, *p*_12_ and *p*_14_ (*p*_13_ is 0 in the model):

$$E\left[X_{11}\right]=\theta_{11}+2\theta_{12};E\left[X_{12}\right]=E\left[X_{14}\right]=\theta_{13}$$

$$m_{1}=E\left[X_{11}\right]+E\left[X_{12}\right]+E\left[X_{14}\right]$$

$$p_{11}=\frac{E\left[X_{11}\right]}{m_{1}};p_{12}=\frac{E\left[X_{12}\right]}{m_{1}};p_{14}=\frac{E\left[X_{14}\right]}{m_{1}}$$

where, as previously indicated, *X* is the random variable that contains the offspring of each type *m* at the first generation, indicated with ${\vec{X}}_{m}\left(1\right)={\vec{X}}_{m}=\left(X_{m,1},\ldots ,X_{m,4}\right)$. The components *X*_*m*,*i*_ represent the number of descendants of type *i* generated by type *m* in one generation, and where each index runs over {1, …,4}.

This can be extended for the other cell types, giving for phenotype 2 (G2H):

$$E\left[X_{21}\right]=\theta_{23}+\theta_{24};E\left[X_{22}\right]=2\theta_{22}+\theta_{23}+\theta_{21};E\left[X_{23}\right]=\theta_{24}$$

$$m_{2}=E\left[X_{21}\right]+E\left[X_{22}\right]+E\left[X_{23}\right]$$

$$p_{21}=\frac{E\left[X_{21}\right]}{m_{2}};p_{22}=\frac{E\left[X_{22}\right]}{m_{2}};p_{23}=\frac{E\left[X_{23}\right]}{m_{2}}$$

For phenotype 3 (G1/2H):

$$E\left[X_{32}\right]=\theta_{33};E\left[X_{33}\right]=2\theta_{32}+\theta_{31}+\theta_{33}$$

$$m_{3}=E\left[X_{32}\right]+E\left[X_{33}\right]$$

$$p_{32}=\frac{E\left[X_{32}\right]}{m_{3}};p_{33}=\frac{E\left[X_{33}\right]}{m_{3}}$$

and for phenotype 4 (P1H):

$$E\left[X_{41}\right]=\theta_{43};E\left[X_{44}\right]=2\theta_{42}+\theta_{43}+\theta_{41}$$

$$m_{4}=E\left[X_{41}\right]+E\left[X_{44}\right]$$

$$p_{41}=\frac{E\left[X_{41}\right]}{m_{4}};p_{44}=\frac{E\left[X_{44}\right]}{m_{4}}$$

### Quantification and statistical analysis

Cells were segmented with the help of Ilastik,^87^ Cellpose^88^ and custom scripts written in MATLAB (The Mathworks). Briefly, timelapse sequences of MG-63-H2BiRFP670 were segmented with Ilastik and tracked by means of a custom script written in MATLAB. Obtained tracks were manually inspected for occasional mistakes. By this means we could either obtain all the transitions observed in each data series or compute the total number of cells at all times and process data as specified in the next section.

MG-63-FastFUCCI timelapse sequences were segmented by creating a linear combination of red and green channel and applying Cellpose.^88^ Then cells were tracked as before and tracks were manually corrected. Red and green signals corresponding to segmented cells were rescaled to avoid time dependent fading (which we found relevant particularly for the green channel). With the obtained point distribution, and in order to define the state (green or red) of each cell, we built a Gaussian Mixture Model with two components for each data series. Cells were then assigned to either class at all time-points. In order to avoid wrong classification we isolated cells that were not clearly assigned to either class (by filtering for the value of the posterior probability of assignment to either class). We found that these would correspond to ’phenotypic switches’, i.e., either cells that just divided, where red intensity was not strong enough yet, or cells that were ceasing to express red to start expressing green. In such cases we assigned the closest future state. Tracks for each data series were either transformed into counts of all transitions or processed as explained hereafter (Figures S4E and S4F).

In order to perform inference on experimental data we first determined the average doubling time *T*_*div*_ for a given set of experiments and then constructed sampling times spaced away by a cell doubling time. Experimental data (once processed and segmented) were expressed as temporal sequences of number of cells *n*(*t*) where the frame rate is either 10 or 20 min. In order to find a set of sampling points to convert the finely resolved experimental data series into BGW branching process-like data series, we identified, in each data series, all regions with $\frac{dn\left(t\right)}{dt}\leq 0$. Once excluded connected regions shorter than 4 h, we identified *n*_*e*_ regions (typically 4 or 5 for each data series), corresponding to generations. For each of these regions we identified the first and last time-point: $\left[t_{2l-1},t_{2l}\right]$, with $l=1,\ldots ,n_{e}$ and the midpoints: $t_{l}^{\left(m\right)}=\left(t_{2l-1}+t_{2l}\right)/2$. The corresponding *n*_*e*_ sampling points ${\hat{t}}_{l}$ were found by minimizing the function:

(Equation 73) $$f\left(\vec{\hat{t}}\right)=\sum_{l}{\left({\hat{t}}_{l}-t_{l}^{\left(m\right)}\right)}^{2}$$

over $\vec{\hat{t}}$, with the constraints:

$${\hat{t}}_{l}<t_{2l}$$

$${\hat{t}}_{l}>t_{2l-1}$$

(Equation 74) $$\left|{\hat{t}}_{l}-{\hat{t}}_{l+1}\right|\leq T_{\mathrm{div}}+\Delta$$

where *T*_*div*_ is the average doubling time over trajectories measured for each cell and Δ is a tolerance set to *T*_*div*_/2. Such a system of constrained nonlinear equations was minimized with MATLAB (see Figure S4C). Data series with misidentified plateau were manually corrected.

For MG-63-FastFUCCI we sampled every half average doubling time in order to accommodate for the cell cycle transitions from G1 to S/G _2_/M. We used the same nonlinear programming solver in MATLAB to impose sampling points at the midpoint of each plateau which was identified by considering regions where the number of red (or green) cells was equal to the total number of cells. Each sampling time was again picked to fall within a plateau and to be a half duplication time away from the following and previous sampling times. Cells trajectories for the case of contact inhibition were only aligned on the first cell division.

Raw data from ref.^68^ were retrieved from the repository cited in the paper at the link provided.^89^ In order to handle the unknown progenitor in the raw data we explored the space of possible initial conditions by creating a grid in probability space with interval 0.1 and picked the initial distribution with the highest likelihood given the observed data.

In order to quantify the expression of our targets in smFISH, nuclei were segmented by means of Cellpose^88^ and cell cytoplasms were reconstructed by growing labeled regions around nuclei without spilling out of the organoid as identified by the bright-field images. Spot detection was performed using the Big-FISH Python package (part of FISH-quant^90^), following the default single-molecule detection pipeline. The number of spots belonging to each cell in an organoid was then fed to a hierarchical clustering algorithm with two classes, using the number of spots corresponding to *LGR5* and *KRT20* as features.

To account for the three-dimensional structure of organoids, independent full 3D stacks of DAPI-stained whole organoids were acquired and segmented in order to create a mapping from the number of nuclei in a randomly selected 2D slice to the total number of nuclei in the corresponding 3D organoid (Figure S6H). The relationship, fitted with a power law, was then used to infer the number of nuclei in the corresponding 3D organoid for each slice of organoid in our smFISH experiment. The ratio between *LGR5*-and *KRT20*-expressing cells found in the 2D slice was preserved in the 3D reconstruction. The initial number of cells right after seeding was measured by counting the number of cells in each seeded object at the microscope, by means of a DAPI staining.

Inference on mCRC organoids was performed using the MCEM method in two sequential steps. In the first step we inferred parameters using data collected at 6 dps, with starting condition generating according to experimental observations, i.e., distribution from Figure S6E and proportions from data at 6 dps. The parameters inferred from the day 6 data were then used to simulate starting conditions to match endpoints data at 10 dps and infer corresponding parameters. From these simulations, we excluded extinct trajectories, defined as those in which the total number of cells is zero. From the MCEM results, we excluded simulations that did not reach convergence on parameter estimates. In addition, we discarded solutions in which *KRT20*+ cells were allowed to back-differentiate with probability equal to 1, as such behavior is biologically unrealistic.

## Supplemental information

Document S1. Figures S1–S17 and Tables S1–S2

Document S2. Article plus supplemental information
