# Supplementary material for: Inference of lineage hierarchies, growth, and drug response mechanisms in cancer cell populations without tracking
Source: Cell Rep Methods. 2026 Aug 17;6(10):101566. doi: 10.1016/j.crmeth.2026.101566 (PMC13721761; doi:10.1016/j.crmeth.2026.101566)
Supplement: Supplementary file 1 [file mmc1.pdf]

Cell Reports Methods, Volume *6*

Supplemental information

Inference of lineage hierarchies, growth, and drug response mechanisms in cancer cell populations without tracking

Andrea Piras, Federica Galvagno, Letizia Pizzini, Matteo Nunziante, Sabrina J. Fletcher, Elena Grassi, Andrea Bertotti, Luca Primo, Antonio Celani, and Alberto Puliafito

Inference of lineage hierarchies, growth and drug response mechanisms in cancer cell populations – without tracking

Andrea Piras[1,2], Federica Galvagno[1,2], Letizia Pizzini[1,2], Matteo Nunziante[1,2], Sabrina J. Fletcher[1,2], Elena Grassi[1,2], Andrea Bertotti[1,2], Luca Primo[1,2], Antonio Celani[2,3,*], and Alberto Puliafito[1,2,**,4]

[1]INOC – Istituto Nazionale Oncologico Candiolo, IRCCS, Candiolo (TO), Italy
[2]Department of Oncology, University of Turin, Candiolo (TO), Italy
[3]The Abdus Salam International Centre for Theoretical Physics ICTP, Trieste, Italy
[4]Lead contact
[*]Correspondence: antonio.celani@unito.it
[**]Correspondence: alberto.puliafito@unito.it

SUPPLEMENTARY MATERIAL

Glossary

**genetic heterogeneity**	existence of multiple genotypes within the same group of cells or tumor
**phenotypic heterogeneity**	existence of distinct cell states or types within the same group of cells or tumor. This can exist also isogenically, i.e. with all identical cells. Also genetic and phenotypic heterogeneity might be linked
**clone**	ensemble of cells deriving from the same common ancestor, i.e. an entire progeny
**lineage**	ensemble of rules and fate decisions possibilities arising from the same ancestral progenitor (see also **lineage hierarchy**)
**pedigree**	full knowledge of the lineage for one particular ancestor, i.e. a clone, which implies the knowledge of all the transitions observed (including phenotypic transitions or switches, cell division events, cell death, etc, along with all cell types at all times). An alternative term used throughout the paper for pedigree is **lineage tree** or simply **tree**. In the language of branching processes, a tree is a particular realization consisting of a sequence of given transitions
**division time**	the time between two consecutive cell division events in a cell
**lineage hierarchies**	the ensemble of all transition probabilities for a lineage. This is also indicated as population structure
**phenotypic state**	distinct set of transcriptional activities for virtually all genes in a cell, i.e. the transcriptional program that defines a relatively stable phenotype and potentially the behavior of a cell. If very stable or irreversible this assumes the meaning of cell type in a developmental sense
**cell type**	a phenotypic state characterized by stability (for example long-lasting or resilient to perturbations) and/or irreversibility (for practical purposes a very strong basin of attraction)
**phenotypic plasticity**	The ability or increased ability of a cell to reversibly alter its cell state in response to intrinsic signals or environmental cues
**tracking**	experimental technique resulting in full determination of a lineage tree or pedigree of a particular cell or ancestor
**phenotypic switch**	transition from one phenotypic state to another
**generation**	within BGW processes this can be thought as the discrete time step separating individuals that performed one transition of any kind. Mapping a generation into a real time is not a univocal procedure. In our manuscript we consider that in a clone, the generation is the shortest division time occurring in the lineage hierarchy. Our definition is closest to what commonly found in the context of developmental biology. Note that cell divisions do not need to be perfectly synchronized in either definition
**transition**	throughout the manuscript we indicate with transitions all possible moves that one cell can undergo, these include phenotypic switches but also division (whether homotypic or heterotypic, symmetric or asymmetric), standbyes and cell death
**individual**	within the language of BGW processes it corresponds to a cell
**type**	we map the notion of BGW type directly into a phenotypic state (or cell type)

Table S1: **Glossary of terminology throughout the manuscript, related to Fig. 1.** We report here a list of the most commonly used terms reviewed according to the recent discussions found in refs.[1–3].

Data types compatible with our approach

a. knowledge of cell types and all transitions for all generations (this case is described as 'tracking' throughout the manuscript and in fig. 1D). Experimentally this situation corresponds to having a fluorescent reporter for each cell-type or state and observing cell types or states at all times, including transitions, division and deaths.

b. knowledge of cell types for all generations with no direct observation of transitions (this case is described as 'consecutive measurements without tracking' in fig. 1E). Experimentally this situation corresponds to having a fluorescent reporter for each cell-type or state and observing cell types or states at all times (without tracking).

c. knowledge of the starting type or state of the cell and final types/states and counts (the case is shown in fig. 1F as connected end-point measurements). In this case it is not required to know the underlying tree, but just the counts and types at the end of the experiment. As such, this scenario contains less information than the previous case and therefore requires more data.

d. knowledge of cell types/counts at different generations all starting from different initial ancestors (the case is shown in fig. 1G as unconnected endpoint measurements). This situation corresponds to fixing different experiments at different timepoints.

e. knowledge of states/types and counts at a late generation with no knowledge of the number of starting cells (this case is associated with little information and a large amount of data is required in order to produce meaningful inference results). If the initial types or states are not known, it is still possible to infer transitions. While theoretically possible this case is not explicitly considered in the manuscript.

f. knowledge of the starting cell type or state, the underlying tree with unobserved types until the last time-point where types are known (see for example[4,5]). This case is experimentally more accessible than the previous one as determination of the tree can occur for example by means of live-microscopy and then cell types or states at the end can be determined with a biological technique of choice which can require fixation (immunofluorescence, *in situ* hybridization,...). Initial state can either be known (for biological prior information) or can also be left undetermined. While theoretically possible this case is not explicitly considered in the manuscript.

Table S2: **List a number of representative data structures, related to Fig. 1** We here report a number of different experimentally relevant situations that our method can tackle. Inferring a lineage from counts only does come at the price of increasing the amount of data needed. Therefore, while theoretically accessible, all of the described experimental situations need to be tackled with the sufficient amount of data, which is difficult to determine *a priori* with systematic criteria. All the situations taken into consideration assume that the existing states or types are known. It is in principle also possible to perform model selection with our approach but this is not explicitly presented in the manuscript.

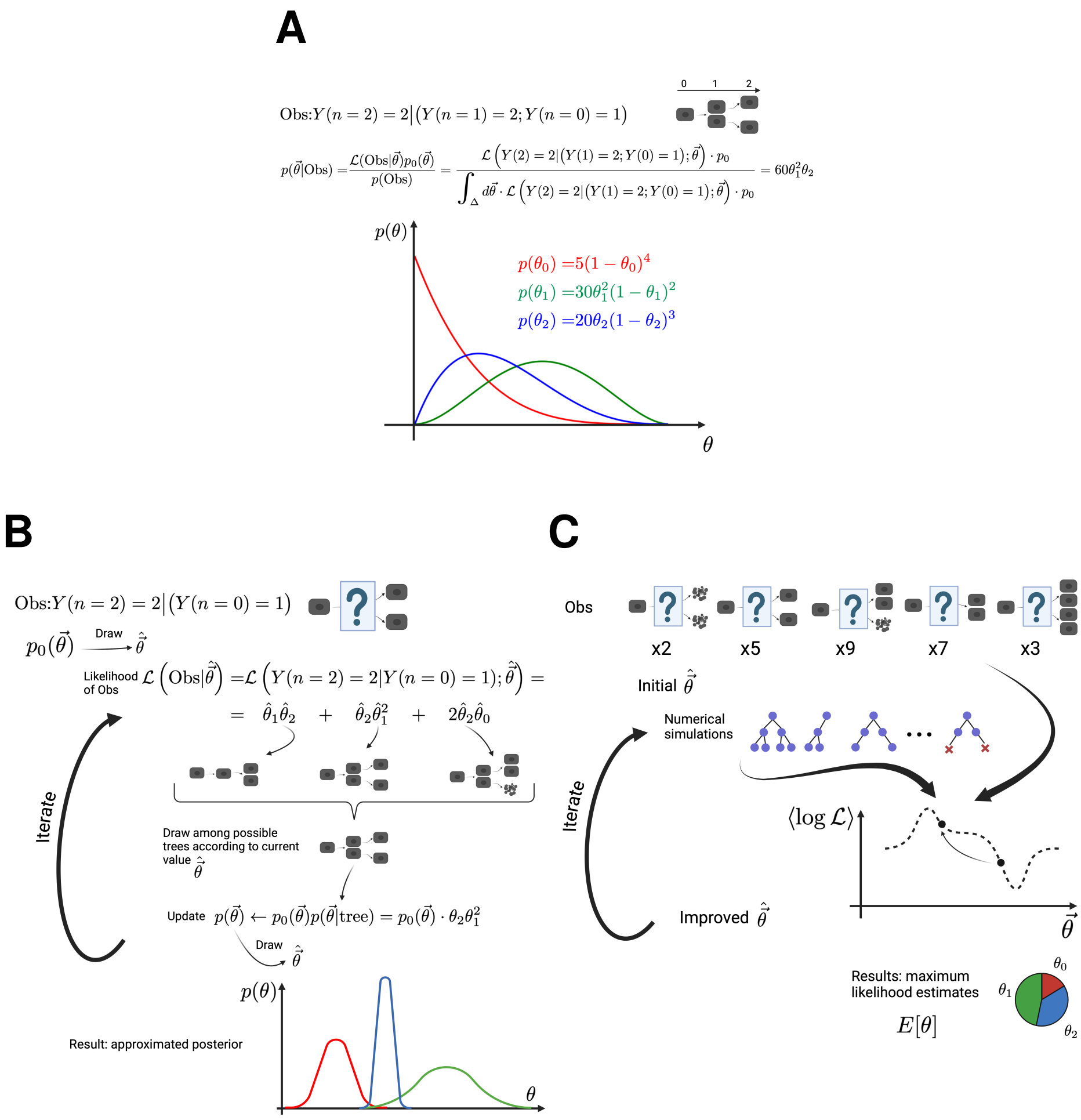

Figure S1: **Bienaymé Galton Watson branching processes and inference for cell populations, related to STAR METHODS. A** Sketch of the methodology to infer transition probabilities with the analytical method. One full observation is assumed, along with associated transitions (one division and two inactivities). The corresponding posterior is explicitly worked out by means of Bayes' formula with a constant prior $p_0(\vec{\theta}) = p_0$. The normalization factor at the denominator is integrated over the parameters simplex (in this particular case a triangle). The posterior is then marginalized to obtain the analytical probability distribution functions for each parameter. Different observation are all factorized into the likelihood and processed in the same way. **B** Sketch of the methodology to infer transition probabilities with the EGS method. With a given observation (which here does not include the number of cells at the first generation) one draws a set of parameters distributed according to the prior. This set of parameters is used to evaluate the probability of each possible tree compatible with the given observation as shown in the sketch. Once one of the compatible trees is drawn the distribution of parameters is updated accordingly, and is then used to draw a new set of values. The procedure is iterated to compute the full marginal PDF of each parameter. **C** Sketch of the methodology to infer transition probabilities with the MCEM method. A set of observations with unknown intermediate states is assumed, along with a multiplicity. A set of parameters is drawn and numerical simulations of the underlying process are performed. These simulations are weighted with the empirical distribution of the observations and contribute to the estimation of the likelihood. The local extrema of the likelihood are searched by means of a surrogate function. This procedure, once converged, outputs the maximum likelihood estimation of each parameter.

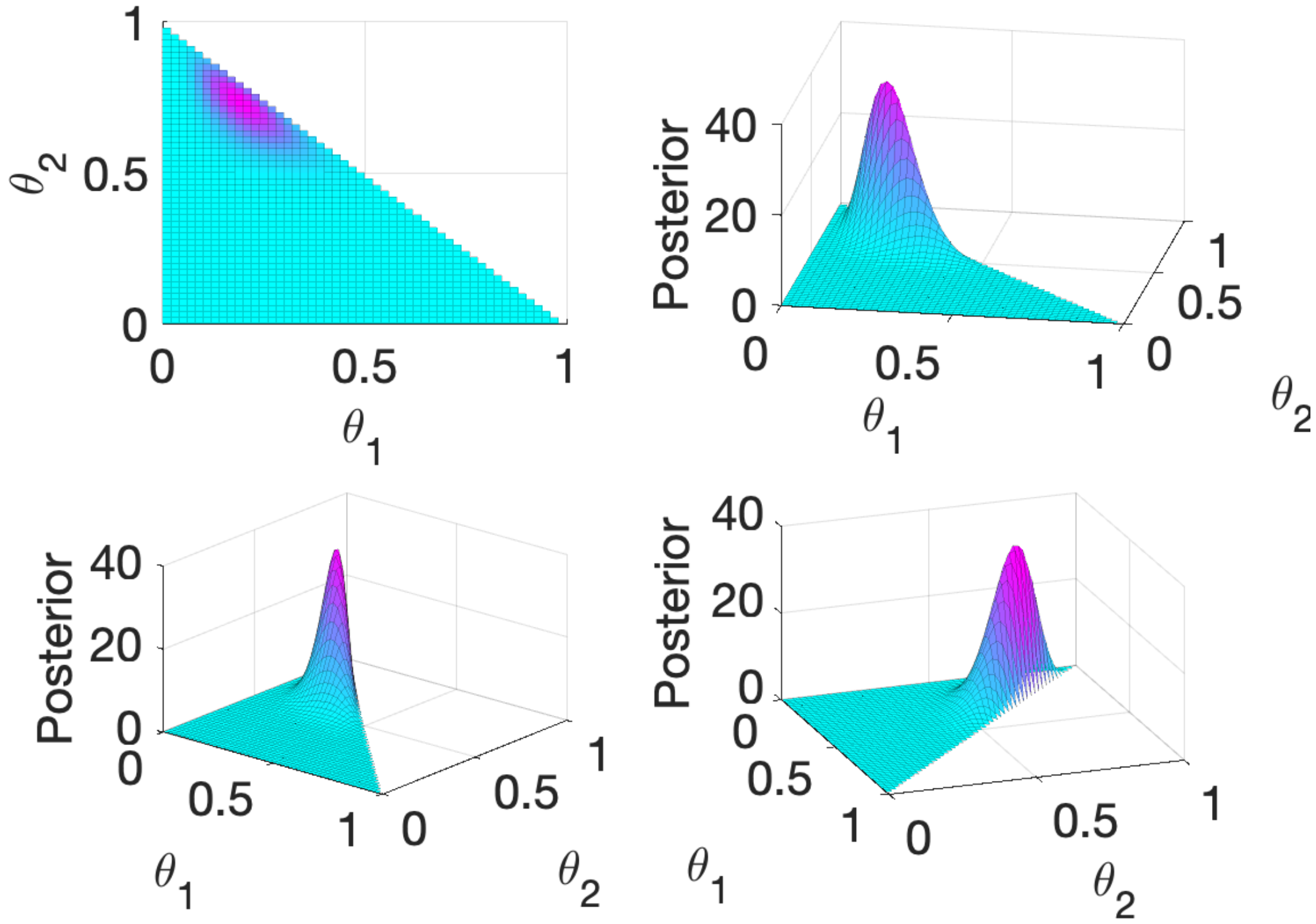

Figure S2: **Illustration of posterior distribution, related to STAR METHODS.** Each panel shows a different view of the multivariate posterior distribution obtained for the function $p(\mathbf{\Theta}|\mathbf{Y}) = \theta_0^2\theta_1^4\theta_2^{16}/\mathcal{N}$, where $\mathcal{N} = \Gamma(3)\Gamma(5)\Gamma(17)$.

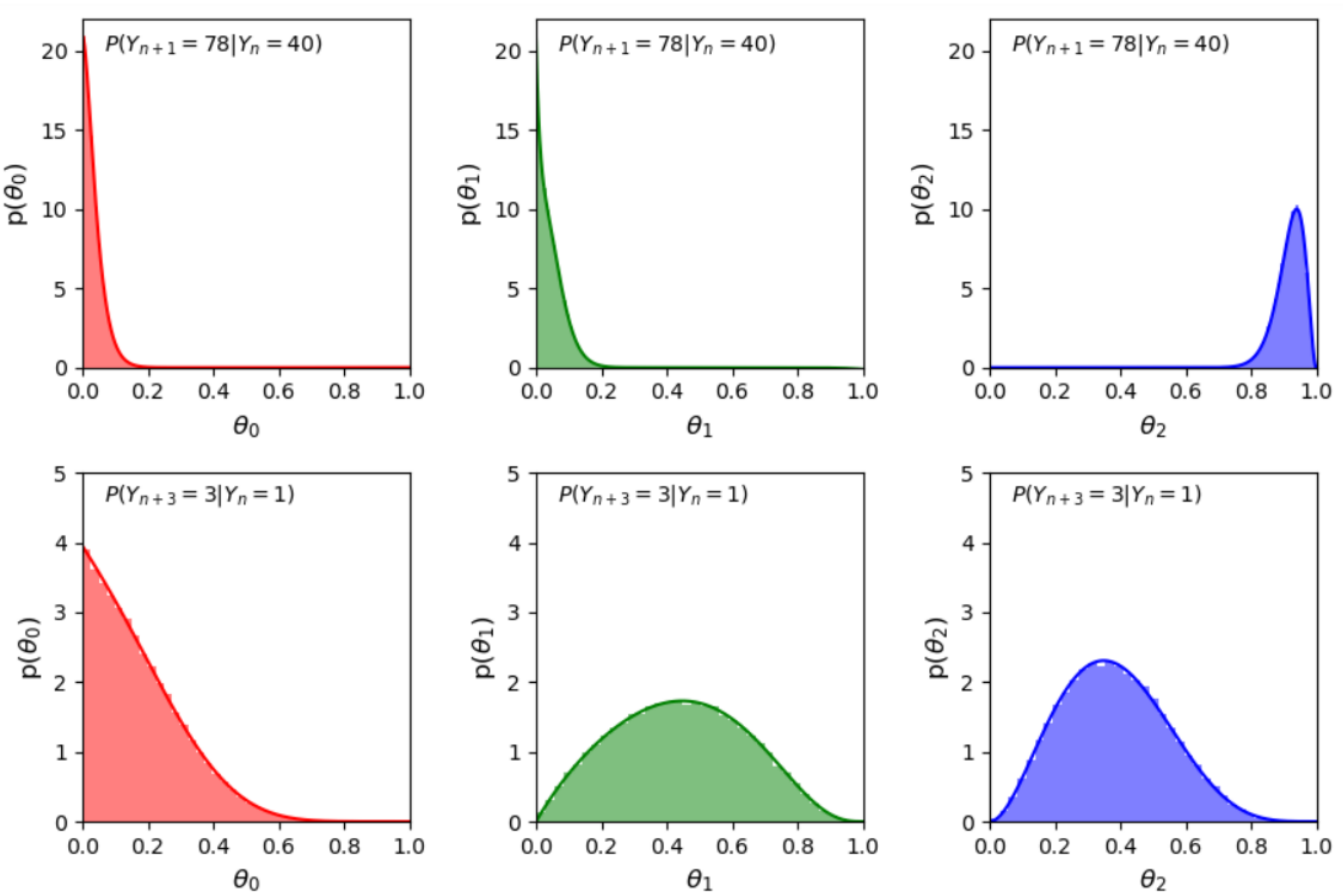

Figure S3: **Comparison between analytical and EGS marginals, related to STAR METHODS.** (Top) Marginal distributions for a BGW with one phenotype where 78 cells are observed after one generation starting from 40 cells. Marginal distributions obtained with EGS (in the lighter colors) are compared with the analytical (plotted in solid color) (Bottom) A case where 3 cells are observed after 3 generations starting with a single cell is considered. A Kolmogorov-Smirnov two-tailed test for EGS marginals computed with 10^5 points tested against discretized (100 points) exact marginal distributions yielded acceptance of the null hypothesis (the two come from equal distributions) at 5% significance level ($p-$value smaller than 10^{-6} for all marginals).

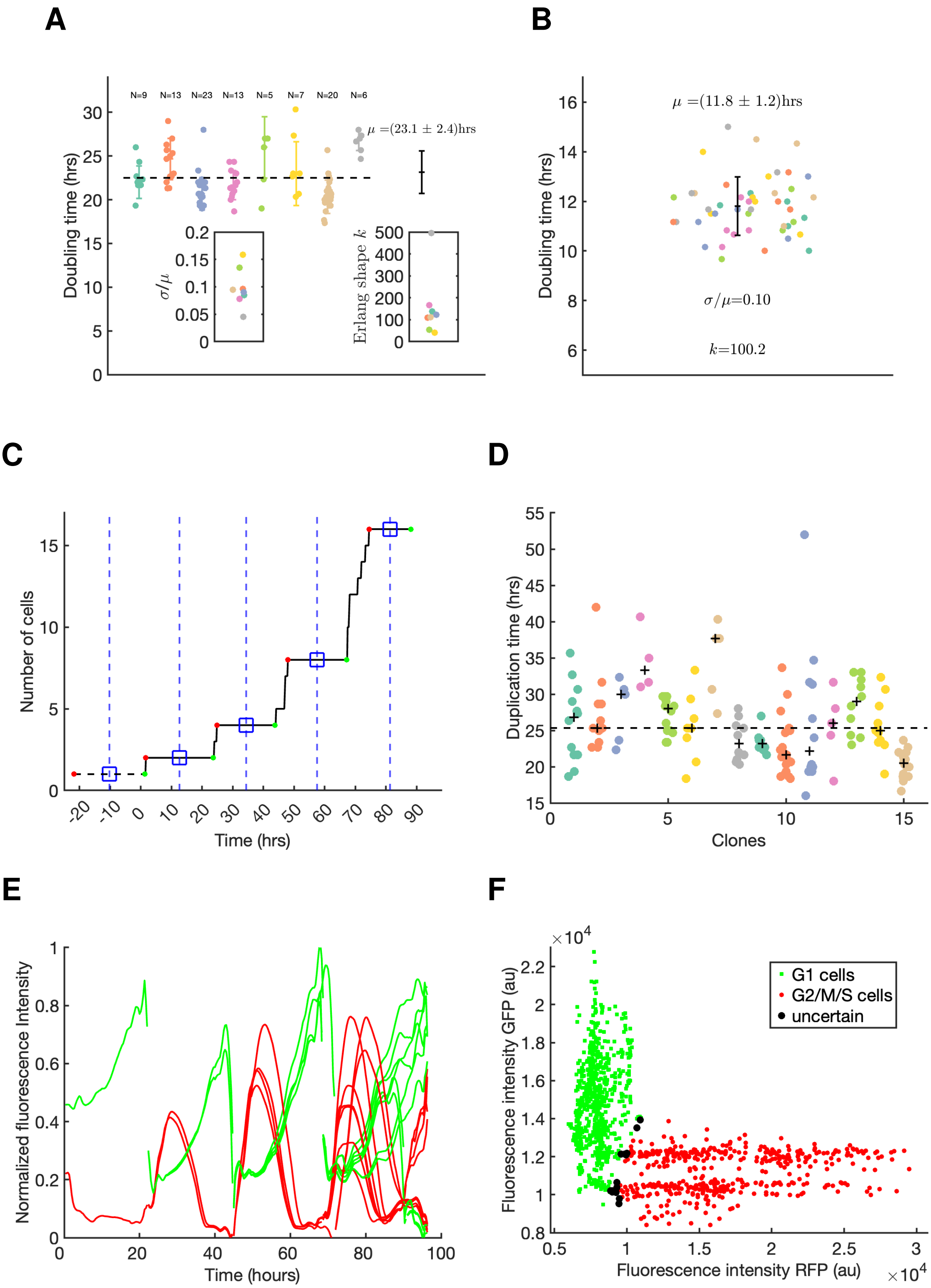

Figure S4: Caption is on the next page.

Figure S4: **Characterization of proliferation statistics and timelapse data processing, related to fig. 4,5. A** Doubling times of offspring of single MG-63 cells, relative to fig. 4A-E. Each cloud plot represents a clone and its offspring. The insets show the relative dispersion in each offspring and the corresponding shape parameter k for an Erlang distribution. **B** Distribution of doubling times for MDCK cells relative to fig.4F-H in the initial free growth phase, along with relative dispersion and corresponding shape parameter for an equivalent Erlang distribution. **C** Distribution of duplication times for each clone relative to fig. 5. **D** Explanatory figure for experimental data processing. The plateaus in the original signal (black continuous line) are found along with start and end points (red and green dots respectively). Sampling points are found as those closest to plateau midpoints (blue empty squares), and spaced with the doubling time. Dashed blue lines correspond to the result of the minimization (see explanation on the STAR METHODS section). **E** Fluorescent signal coming from single cells with a common single ancestor during a timelapse experiment. **F** Scatter plot of green vs red channel signal for a given clone in the timelapse.

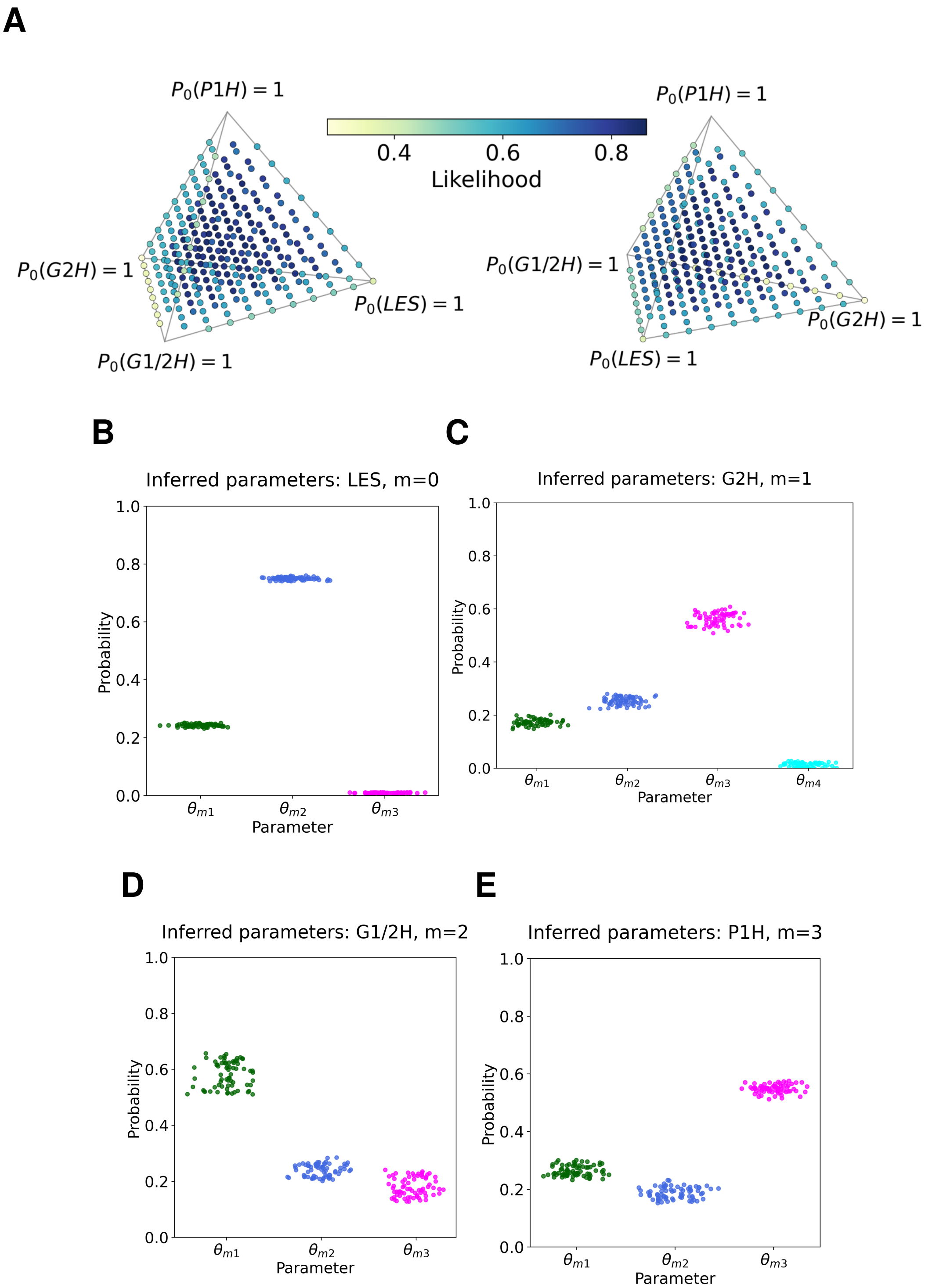

Figure S5: **Dependence of inference on initial ancestor distribution, related to fig. 6. A** Likelihoods for different uniformly spaced values for initial distribution of ancestors. Inner dots represent states with similar probabilities for all cell types, while edges represent extreme values (one or more types not appearing as ancestor). Missing points (specifically on vertices) were excluded from simulations as these specific starting conditions were unable to explain observed data under the chosen model. **B-E** Distribution of inferred parameters for each cell type for the top 25% of likelihoods shown in panel A.

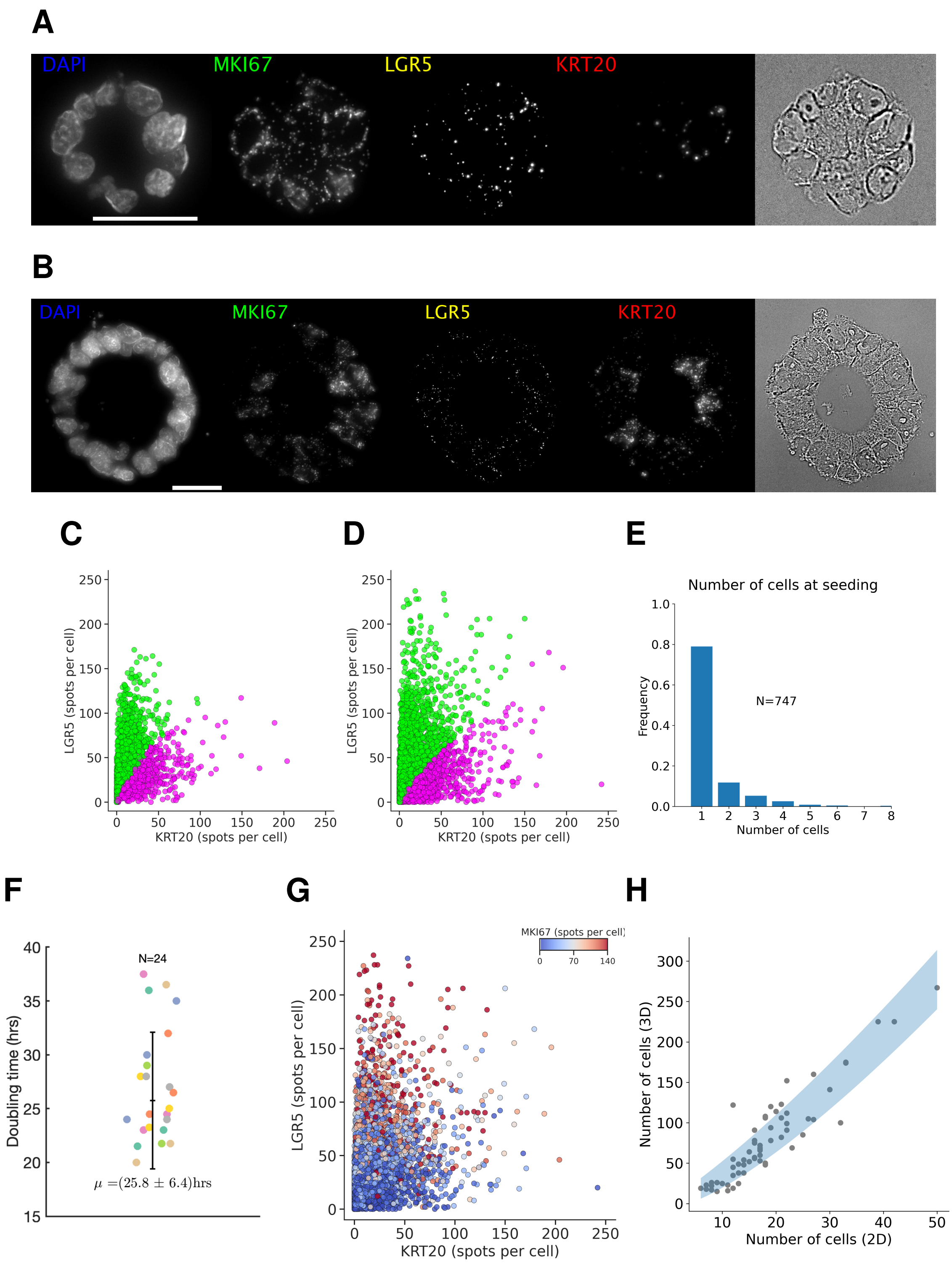

Figure S6: **Phenotypic characterization and growth dynamics of organoids, related to fig. 6.** **A,B** Representative images of smFISH performed on organoid sections with all separate channels indicated within each panel at 6dps and 10dps. Scale bar: 30 μm. **C-D** Clustering of cells as *LGR5*+ (green) or *KRT20*+ (magenta) in each separate time-point. **E** Histogram of the number of cells at seeding in each prospective organoid. **F** Doubling times of offspring of single cells in the patient-derived organoid CRC0322. **G** Scatter plot of all cells within organoids relative to figure 7, with color coding for MKI67 expression (in shades from blue to red). **H** Mapping of 2D slices into entire 3D organoids. Confocal 3D stacks of entire organoids are segmented and then corresponding 2D slices are randomly selected in order to create a correspondence between 2D and 3D objects.

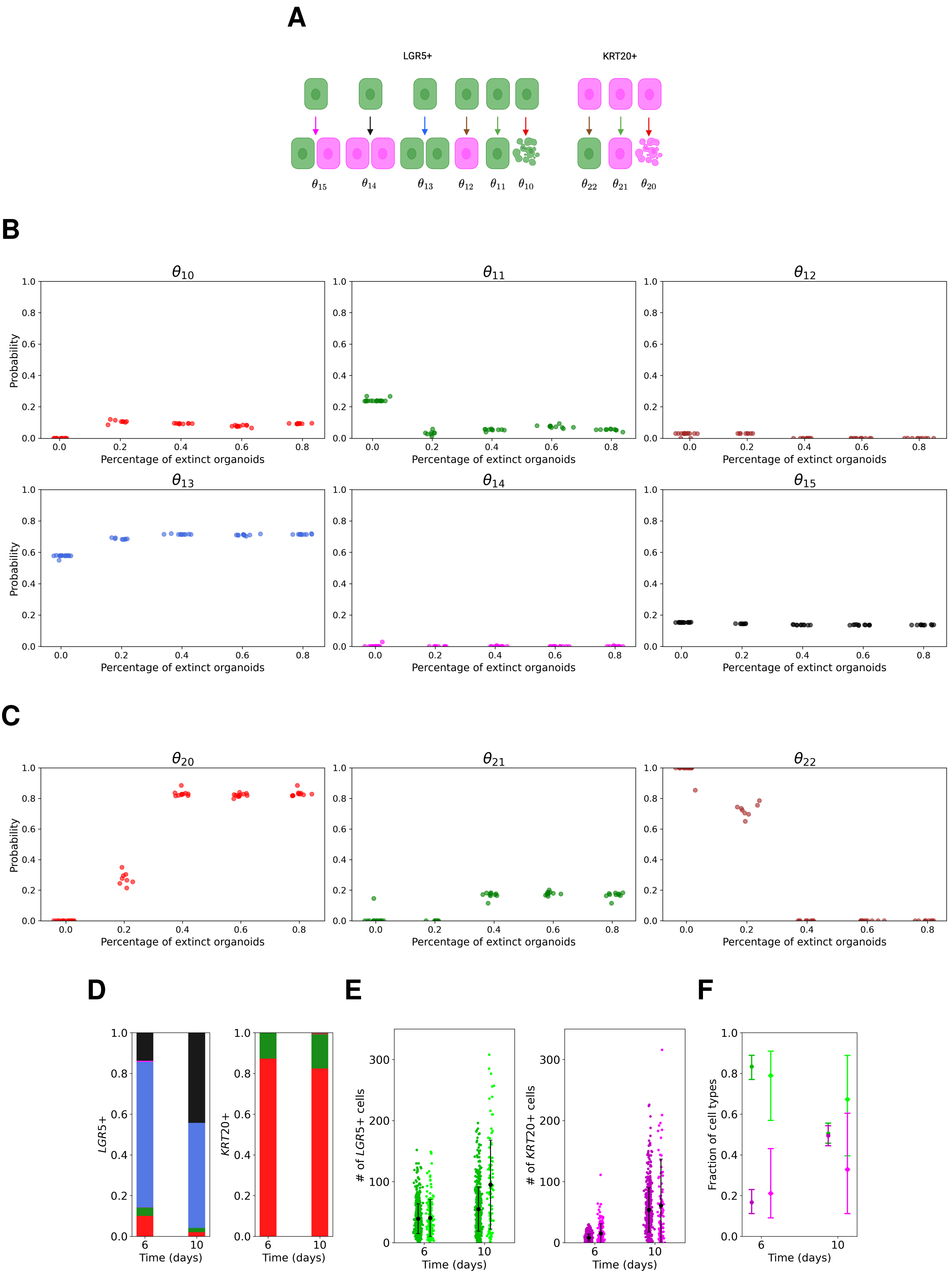

Figure S7: Caption on the next page.

Figure S7: **Impact of extinction probability and parameter estimation in organoids, related to fig. 6. A** Minimal model used for inferring data from colorectal cancer organoids as in fig. 6H. Each color in the arrows correspond to unique parameters in the following panels. Schematic created with BioRender.com. **B,C** Inference of parameters referring to minimal model as in panel (A) from organoids grown for 6 days post seeding imposing different percentage of extinct organoids. Panel **B** refers to *LGR5*+ cells, while panel **C** to *KRT20*+ cells. Note that within experimentally observed extinction probabilities (between 70% and 90%), inference of parameters is stable. **D-F** Results from inference corresponding to the alternative solution found for experimental data. Colors match those of arrows in panel A.

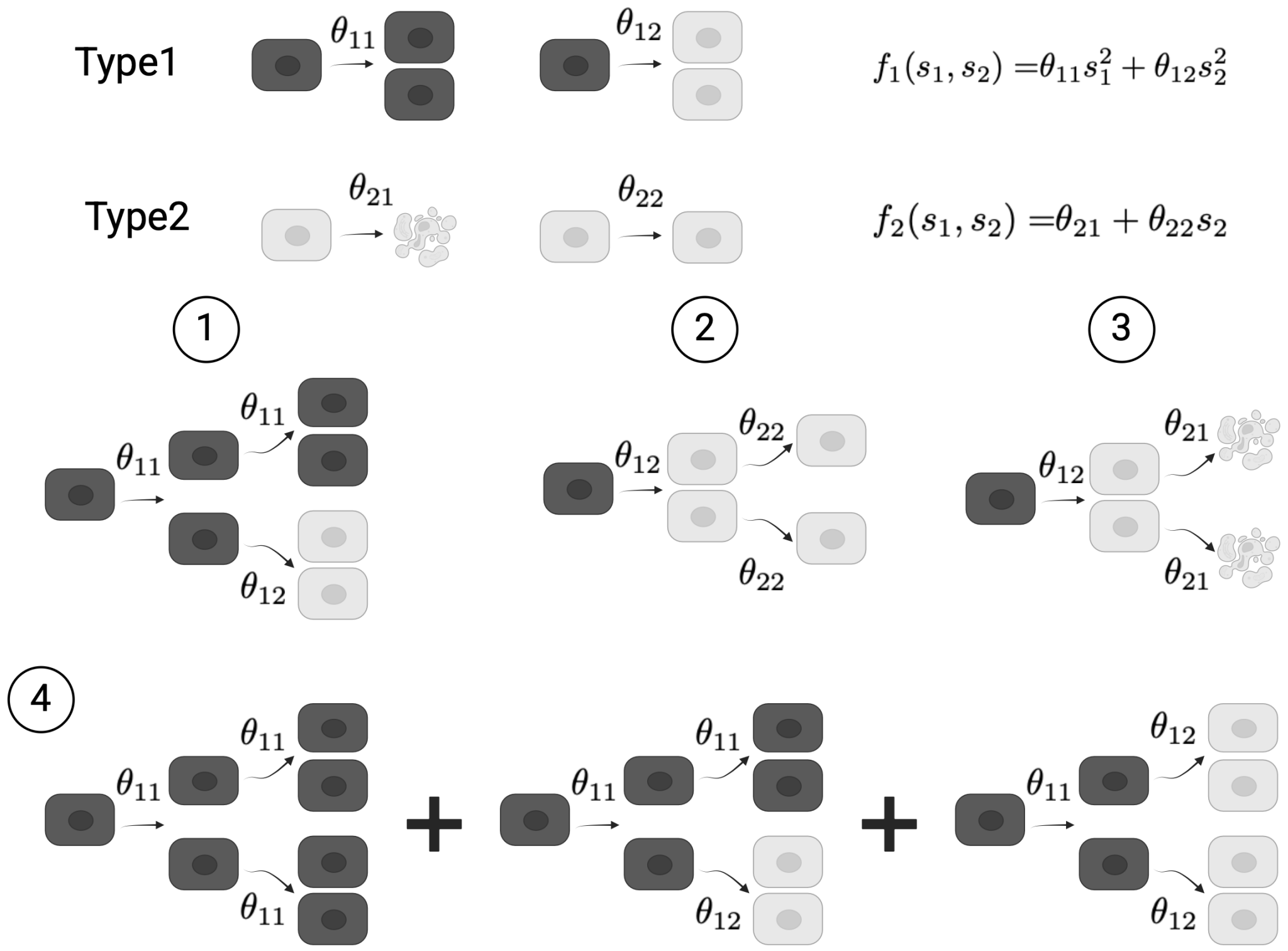

Figure S8: **Calculation of the likelihood for a 2-type BGW process, related to STAR METHODS.** In this example, admitted transitions for each phenotype (1 and 2) are drawn along with their respective PGFs (top). Circled numbers represent 4 different observations corresponding to different trees at the 2nd generation with a particular number of individuals for each phenotype. Schematic created with BioRender.com.

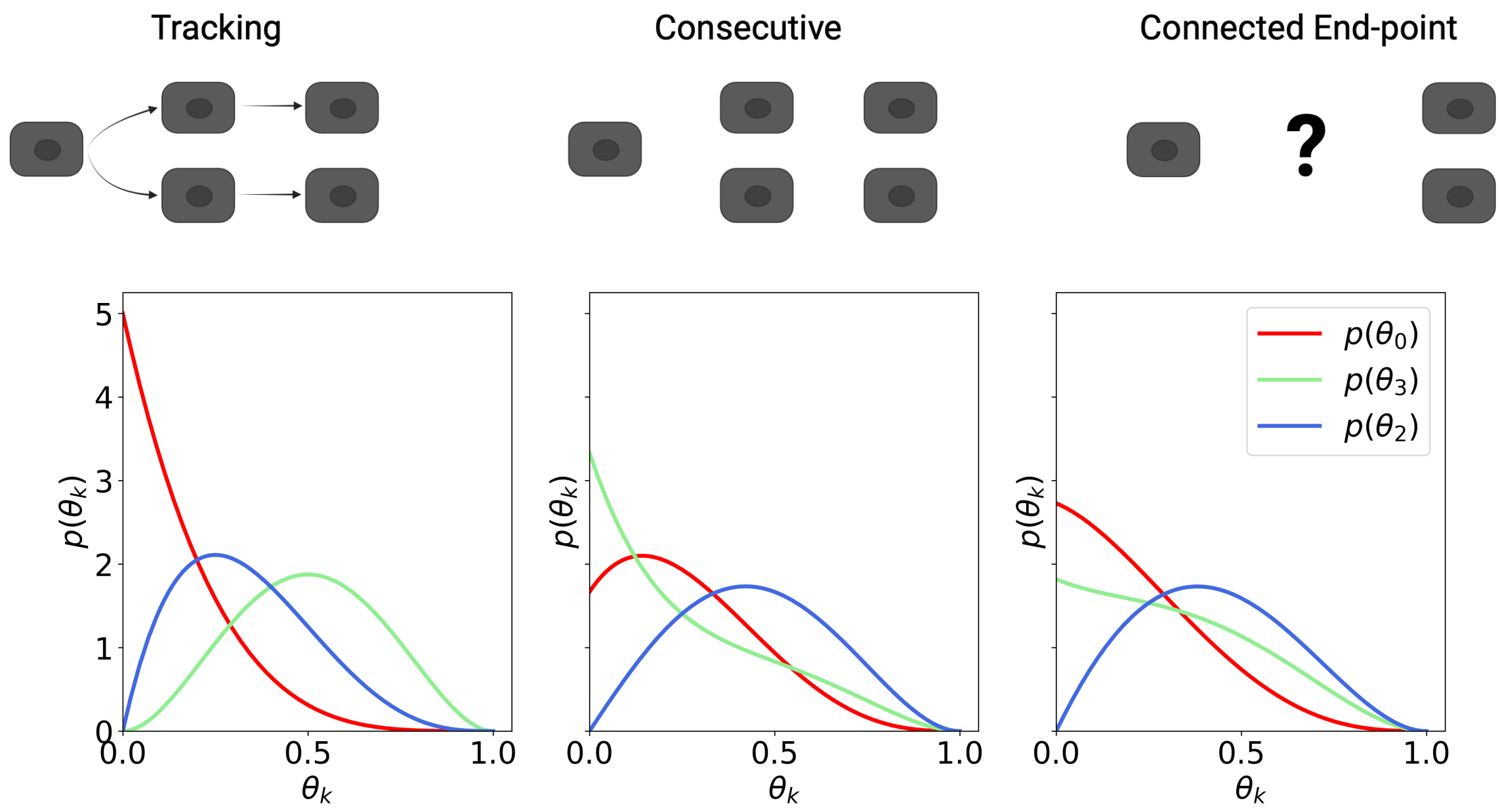

Figure S9: **Analytical inference for three different data-types, related to STAR METHODS.** Observations are indicated on top of the figure and the corresponding set of marginal posteriors is drawn below each corresponding sketch. Schematic created with BioRender.com.

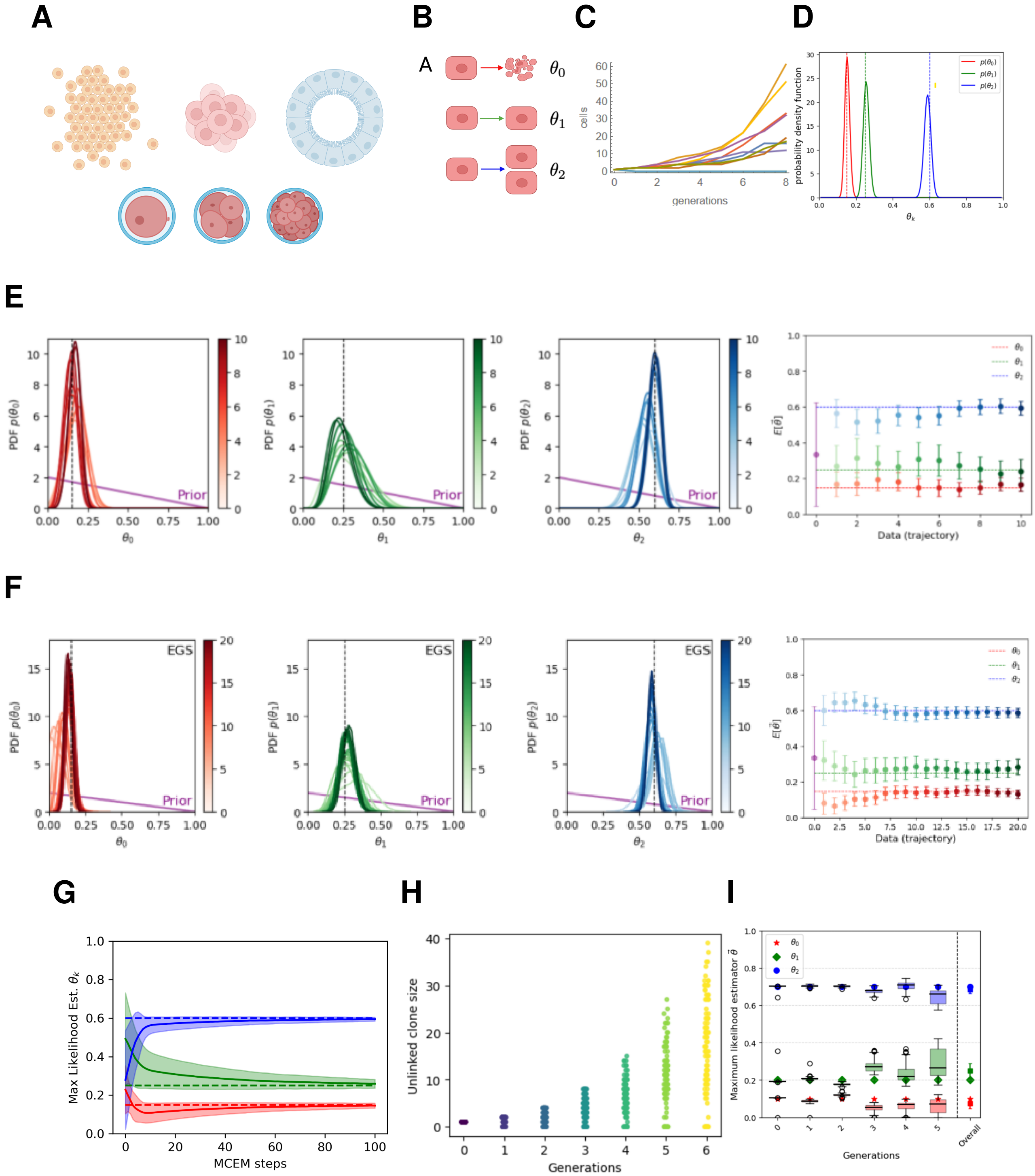

Figure S10: Caption is on the next page.

Figure S10: **Free expansion of a 1-type cell population, related to STAR METHODS. A** Illustration of biological applications of the 1-phenotype BGW process (top row from left to right: a cell colony, a spheroid or tumoroid, a cyst; bottom row: a developing embryo). Schematic created with BioRender.com. **B** Transitions in a 1-type BGW process representing situations as in panel A. True values used were $\vec{\theta}^{\,\text{true}} = (0.15, 0.25, 0.6)$. Schematic created with BioRender.com. **C** Plot of 10 different realizations of a 1-phenotype BGW branching process. All simulations start with one cell and stop after 8 generations. **D** Marginal distributions obtained with the growth curves shown in C, assuming single cell tracking has been performed. **E** *Left* Analytical marginal posteriors for each iteration are shown in different shades of color and peak more and more as the number of processed data increases. Data are the same as in panel C with no tracking information. *Right* Mean value of the inferred marginals for each parameter during the iterative process, compared to the respective true value (dashed lines). **F** *Left* Marginal posteriors computed with EGS by extending the dataset and considering 10000 drawing points. *Right* Comparison between the mean of the distributions and the respective true value. **G** Maximum-likelihood estimations of the three parameters performed with the MCEM algorithm. The dataset consists in 1000 endpoint measurements after 8 generations with no access to intermediate states and same true values. **H** Representation of data collection where each dot is one colony or tumoroid independent from all others. **I** Inference of parameters from data in panel H obtained by using MCEM algorithm. Note that inferred values are consistent with the true values $\vec{\theta}^{\,\text{true}} = (0.1, 0.2, 0.7)$.

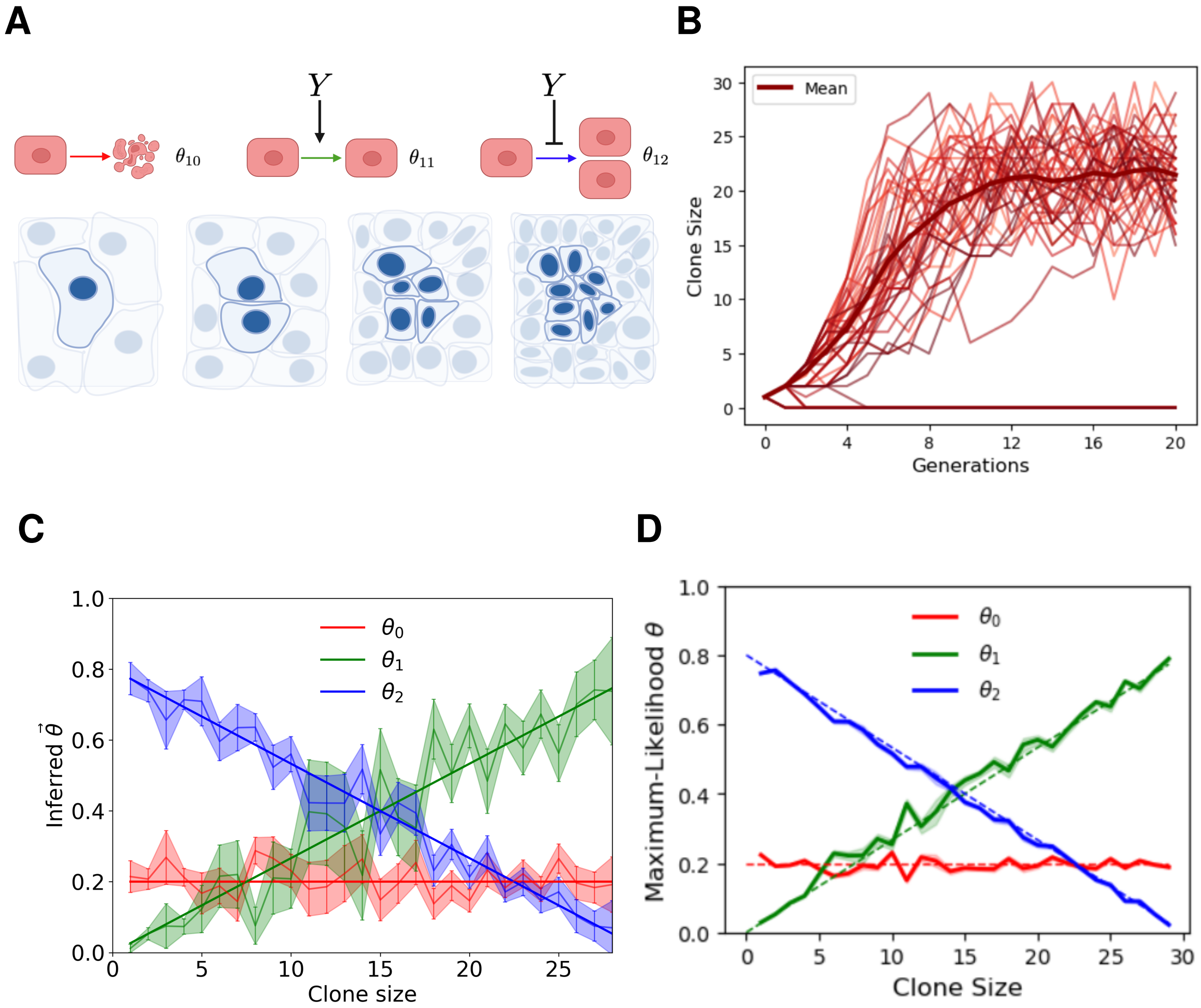

Figure S11: **Constrained expansion of cell clones, related to STAR METHODS. A** (Top) Sketch of the constrained BGW model, where the total number of cells impacts on the probability of inactivity and proliferation. (Bottom) Illustration of the clonal growth in a confluent tissue, which represents the experimental counterpart of the data-series generated by numerically simulations. Schematic created with BioRender.com. **B** Clone size as a function of the generation for the different realizations of the BGW process. Each clone starts from one cell, with parameters $\vec{\theta} = (0.2, 0, 0.8)$ and maximum clone size $K = 30$ cells. **C** Comparison between true and inferred parameter values obtained with the EGS: the true values are represented by dark solid lines, while the estimates are shown in lighter hues with colored regions indicating the corresponding standard deviation of the marginal distributions. **D** Comparison between true and inferred parameter values obtained with the MCEM method. True values are represented by dashed lines, while the estimates are shown in continuous lines.

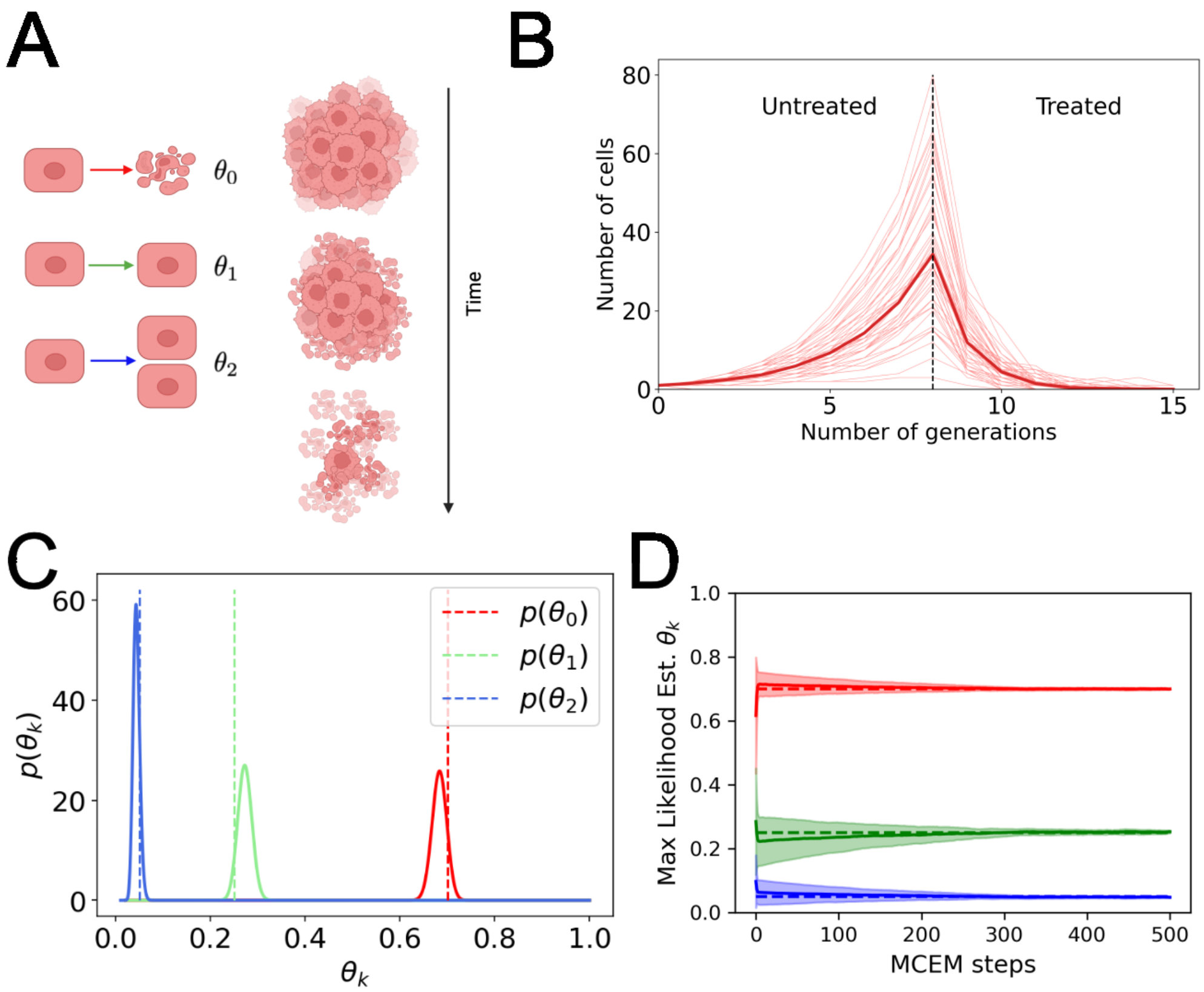

Figure S12: **Inferring the effect of a drug on a 1-type cell populations, related to STAR METHODS. A** Sketch of experimental settings for a 1-type population model for inferring transition probabilities when applying a drug or compound or growth factor. Schematic created with BioRender.com. **B** Numerical simulations showing the growth of a cell population with given parameters $\vec{\theta}^{\text{ true}} = (0.05, 0.35, 0.6)$ which after 7 generations is treated with a drug with $\vec{\theta}^{\text{ true}} = (0.7, 0.25, 0.05)$. **C** Bayesian inference of parameters in a 1-type setting, corresponding to 12 replicates of data series starting from 50 cells for 3 generations. The likelihood corresponds to $\mathcal{L} = \theta_0^{620}\theta_1^{247}\theta_2^{39}$. Vertical dashed lines represent the true values. **D** MCEM inference for a treated 1-type cell population. Dashed lines represent true values of the numerically simulated data.

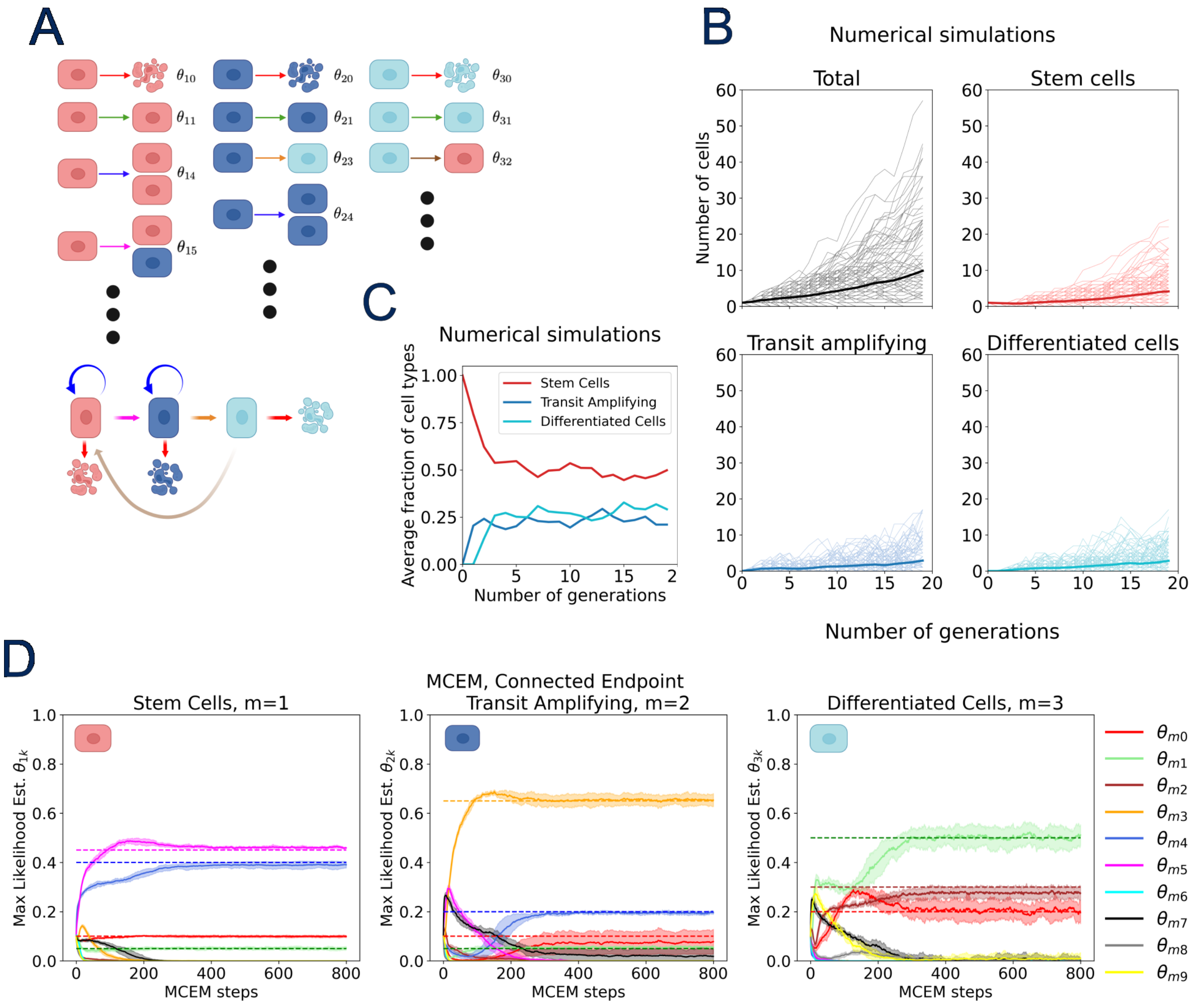

Figure S13: **The growth of a multi-type tumor, related to STAR METHODS. A** All possible transitions considered are here indicated with their corresponding probabilities. Schematic created with BioRender.com. **B** Numerical simulation of the process in panel A: (black) total population size; (red) stem cell compartment; (blue) transit amplifying compartment; (cyan) differentiated compartment. The parameters used for the numerical simulation are $\vec{\theta}_1^{\text{true}} = (\theta_{10} = 0.1, \theta_{11} = 0.05, \theta_{12} = 0.45, \theta_{14} = 0.4)$, $\vec{\theta}_2^{\text{true}} = (\theta_{20} = 0.1, \theta_{21} = 0.05, \theta_{23} = 0.65, \theta_{24} = 0.2)$ and $\vec{\theta}_3^{\text{true}} = (\theta_{30} = 0.2, \theta_{31} = 0.5, \theta_{32} = 0.3)$, with all other unspecified parameters being zero. **C** Average fraction of stem cells (red), transit amplifying (blue) and differentiated cells (cyan) over the course of each simulation. **D** Maximum likelihood estimates obtained with MCEM on data generated as in panel A for stem (left), TA (middle) and differentiated (right) cells. Colors code for different parameters (transitions) as shown in the legend and match the color of the arrows in panel A.

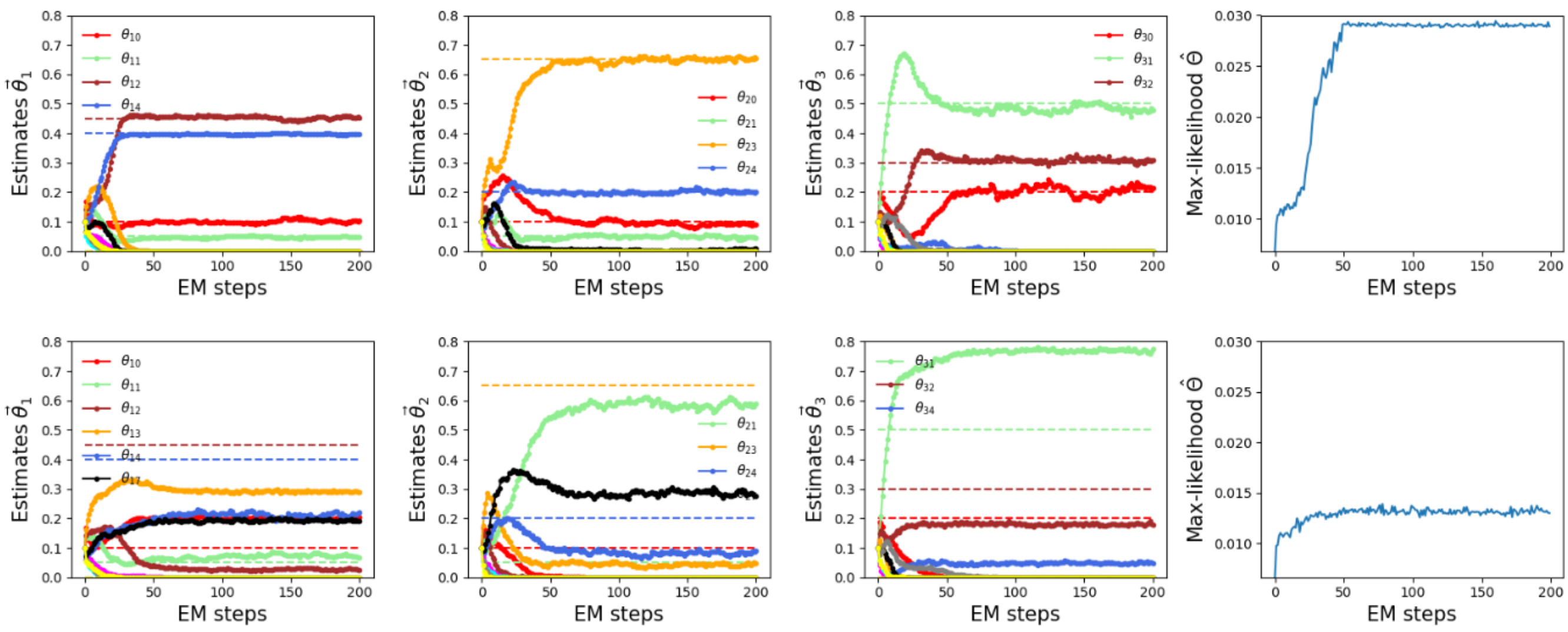

Figure S14: **Convergence of the MCEM, related to STAR METHODS.** A three-type BGW process is used to perform inference. The number of parameters to infer is thirty (see main text). (Top) The inference converges to values close to the true ones. The right plot shows the corresponding value of the likelihood. (Bottom) A case where the same initial parameters, but different data samples, lead to a different set of inferred parameters which however correspond to a lower value of the likelihood.

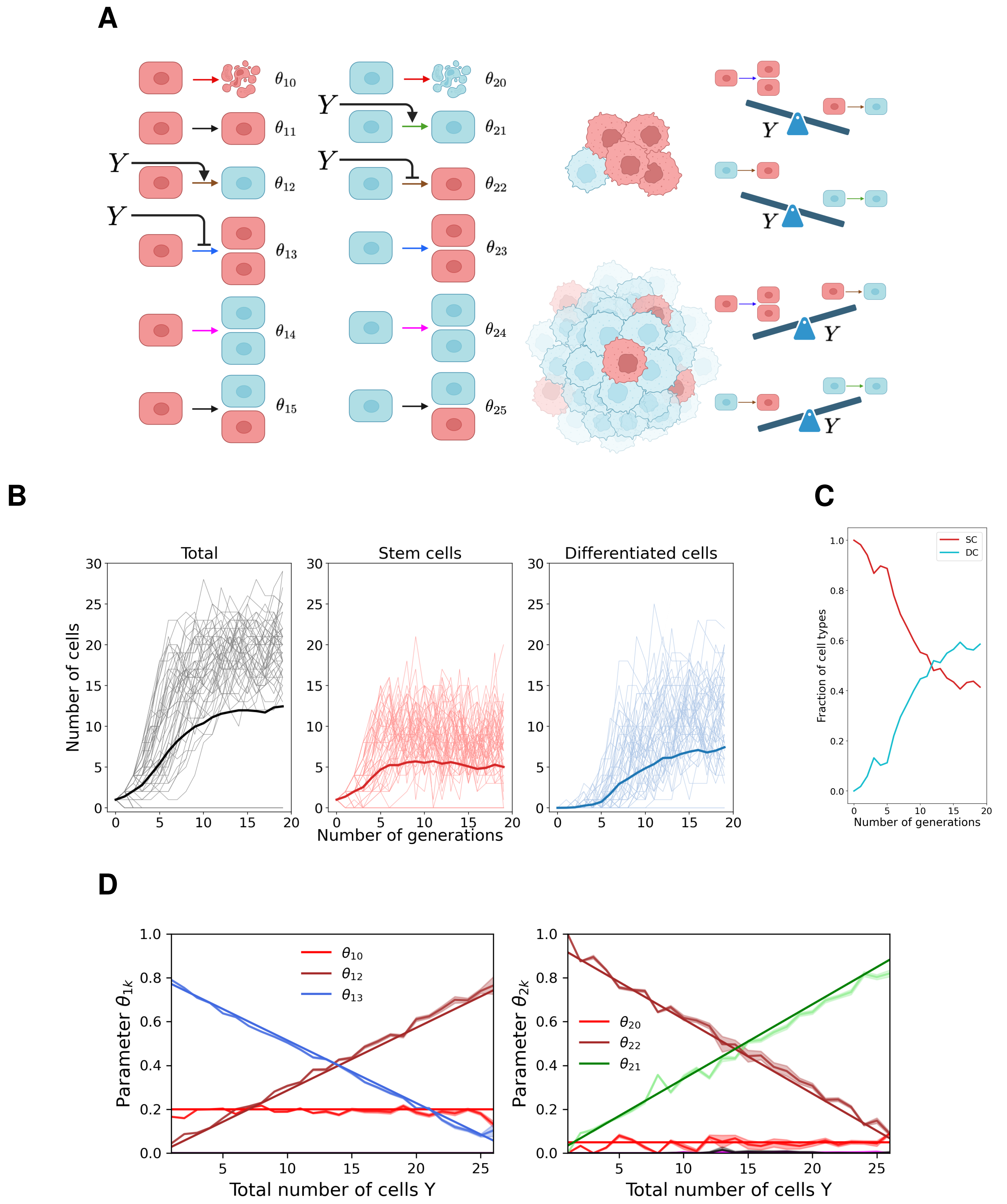

Figure S15: **Feedback on total population size, related to STAR METHODS. A** Sketch of the model used with interactions modeled with a feedback on the total number of cells Y. The feedback is such that higher number of cells favor some transitions and inhibit others, as sketched. Schematic created with BioRender.com. **B,C** Numerical simulations of the process shown in A, along with the fraction of stem vs differentiated cells which is changing over time. **D** Inference of parameters for stem cells (left) and differentiated cells (right) as a function of the total population size Y.

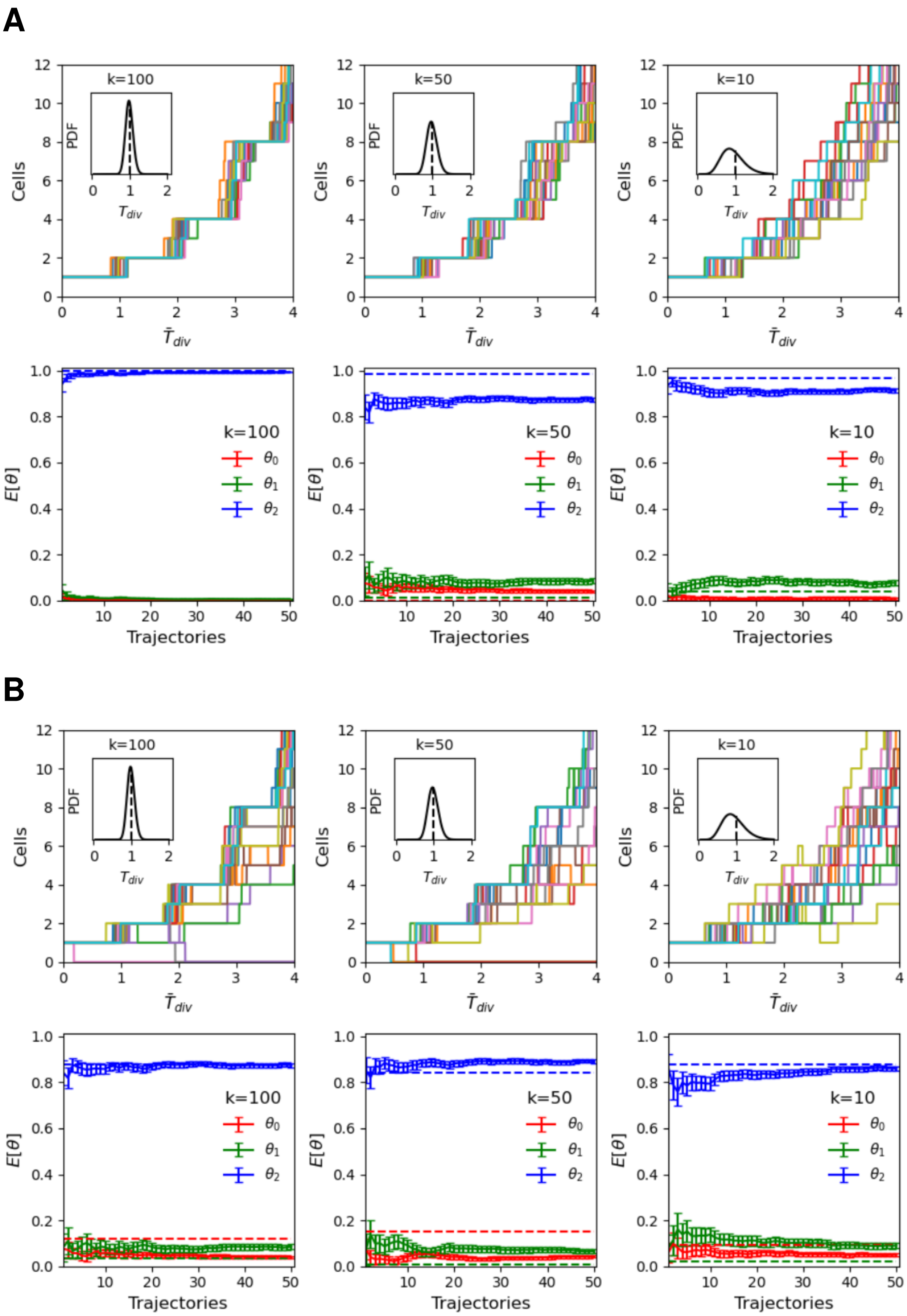

Figure S16: **Using BGW inference with asynchronous generation data, related to STAR METHODS. A** Numerical simulations of a birth process with cell doubling times distributed according to an Erlang distributions with shape parameters $k = 100, 50, 10$. Inference performed on 50 trajectories 6 generations long. **B** Numerical simulations of a birth-death process with cell doubling times distributed according to an Erlang distributions with shape parameters $k = 100, 50, 10$ and Poissonian deaths. Inference performed on 50 trajectories 6 generations long.

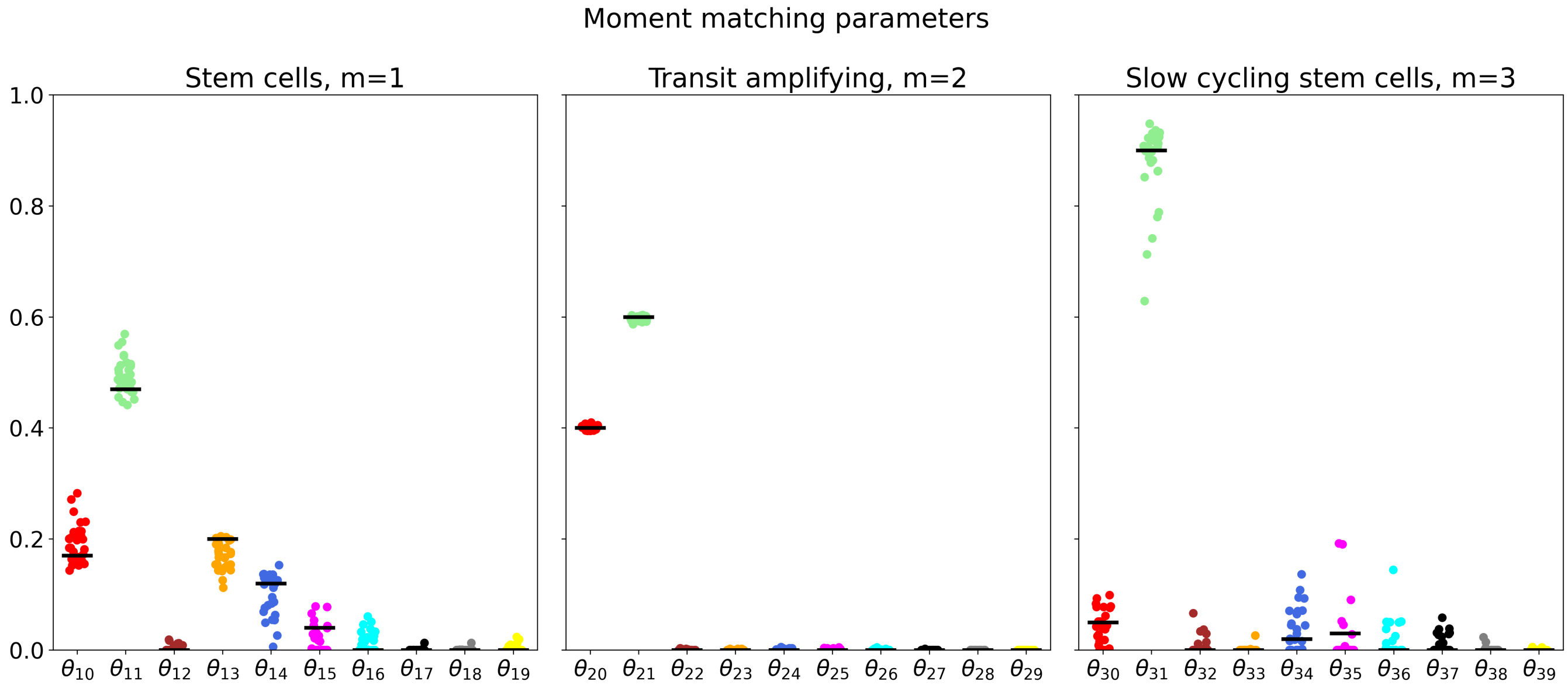

Figure S17: **Parameter estimation with moment matching technique, related to STAR METHODS.** Values of parameters obtained (colored points) for each parameter by means of moment matching explained in the text. The corresponding true value is plotted as a black dash. Each solution is found by performing sub-sampling on data generated with numerical simulations with different initial conditions.

Supplemental References

1. Zeng, H. (2022). What is a cell type and how to define it? Cell *185*, 2739–2755.
2. Trapnell, C. (2015). Defining cell types and states with single-cell genomics. Genome research *25*, 1491–1498.
3. Dance, A. (2024). What is a cell type, really? the quest to categorize life's myriad forms. Nature *633*, 754–756.
4. Hormoz, S., Desprat, N., and Shraiman, B.I. (2015). Inferring epigenetic dynamics from kin correlations. Proceedings of the National Academy of Sciences *112*, E2281–E2289.
5. Hormoz, S., Singer, Z.S., Linton, J.M., Antebi, Y.E., Shraiman, B.I., and Elowitz, M.B. (2016). Inferring cell-state transition dynamics from lineage trees and endpoint single-cell measurements. Cell systems *3*, 419–433.
